# Mapping free energy regimes in electrocatalytic reductions to screen transition metal-based catalysts†
†Electronic supplementary information (ESI) available: Supplemental thermochemical calculations, electrochemical data and simulations and Cartesian coordinates of DFT-optimized intermediates. See DOI: 10.1039/c9sc01766f


**DOI:** 10.1039/c9sc01766f

**Published:** 2019-06-27

**Authors:** Srinivasan Ramakrishnan, Ross A. Moretti, Christopher E. D. Chidsey

**Affiliations:** a Department of Chemistry , Stanford University , Stanford , CA 94305 , USA . Email: chidsey@stanford.edu

## Abstract

The free energy landscape of catalytic intermediates in the two-electron reduction of proton donors and/or CO_2_ to H_2_, CO and HCO_2_^–^ is mapped with density functional theory to screen catalyst candidates from a library of different transition metals and ligands. The goal is to minimize the free energy corrugations between reactants, catalytic intermediates and each desired product, simultaneously screening against intermediates with low free energy that would be traps, and against necessary intermediates with high free energy. Catalysts are initially screened for those with: (a) standard state free energy of the metal hydride intermediate ergoneutral with HCO_2_^–^, which is the lowest energy product with weak proton donors, and (b) standard free energy of the metal carbonyl intermediate sufficiently high to avoid trapping. The design method is tested on a diverse range of ligands including cyclopentadienyl, polypyridyl, amino, phosphino and carbonyl ligands, around three earth-abundant d^6^ transition metal ions, Mn(i), Fe(ii) and Co(iii), using the BP86 density functional, the double-zeta 6-31+G* basis, LANL2DZ effective core potential on the metals and SMD polarizable continuum model for acetonitrile as solvent, which have previously provided chemically accurate values of several redox potentials, p*K*_a_'s and ligand exchange equilibria for transition metal complexes. Among the 36 complexes screened, an Fe(ii) center ligated to two bipyridines and a pyridine with a solvent-bound sixth coordination site for hydride formation from phenol as the proton donor is identified as a promising candidate for ergoneutral hydride formation without trapping by CO. The redox-active bipyridine ligands are predicted to provide near ergoneutral sites for accumulating the two electrons needed to form the hydride. To test the predictions, an Fe(ii) complex was prepared with the desired ligand environment using a pentadentate ligand to prevent ligand exchange. The synthesized complex was indeed found to be active towards electrocatalytic proton reduction as well as CO_2_ reduction at the predicted redox potentials with no trapping by CO. However, contrary to the *in silico* predictions, we found electrochemical evidence of CO_2_ binding after the first reduction leading to CO production. Mapping the free energies of key catalytic intermediates such as the metal hydride and metal carbonyl species by using density functional theory (DFT) serves as a first step in catalyst screening spanning large libraries of metals and ligands. In order to screen against all the intermediates in the catalytic pathway, such as reduced metal-bound CO_2_ intermediates, further refinement and validation of the DFT methods are needed.

## Abstract 2

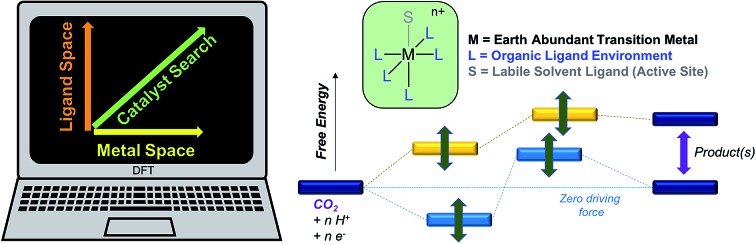
The free energy landscape of catalytic intermediates in the two-electron reduction of CO_2_ and proton donors is mapped with density functional theory to screen catalyst candidates from a library of transition metals and ligands.

## Introduction

The multi-electron reduction of abundant feedstocks such as protons and CO_2_ to fuels and value-added products^1^–^3^ requires catalysts that can rapidly mediate these reductions with high selectivity and energy efficiency. Several earth-abundant heterogeneous electrocatalysts such as metallic copper can catalyze CO_2_ reduction, but often suffer from poor selectivity and energy efficiency.^4^–^7^ Furthermore, the challenge of knowing the exact chemical nature of the catalytic active site on metal surfaces makes the rational optimization of such catalysts difficult. On the other hand, with transition metal complexes, one can in principle tune the relative energies of the catalytic intermediates by simply changing the metal and ligand combination, choosing from a vast library to favor one stoichiometric product over another. While heterogeneous catalysts may continue to be more practical, molecular complexes serve as excellent model systems to advance single-site catalyst design.

The multi-electron reduction of CO_2_ to methanol or C_2_ products is the ultimate goal, but currently no strategies exist to precisely design catalysts that can achieve these transformations rapidly with optimal selectivity. Therefore, as a first step towards this process, we choose the two-electron reduction of protons to H_2_ and of CO_2_ to HCO_2_^–^ and CO (eqn (1)–(3)) as the target reactions for catalyst design, given the availability of mechanistic information for these individual reactions. Furthermore, these transformations involve common intermediates, which provide useful handles to reduce the complexity of the design process. Interestingly, there are many more known single-site molecular electrocatalysts for H_2_ and CO production^8^–^11^ than for HCO_2_^–^ production,^12^–^17^ and even the few examples of the latter involve precious metals and require high overpotentials.

1 HA + CO_2_ + 2e^–^ → HCO_2_^–^ + A^–^

2 2HA + 2e^–^ → H_2_ + 2A^–^

3 HA + 2CO_2_ + 2e^–^ → CO + HCO_3_^–^ + A^–^

**Scheme 1:**
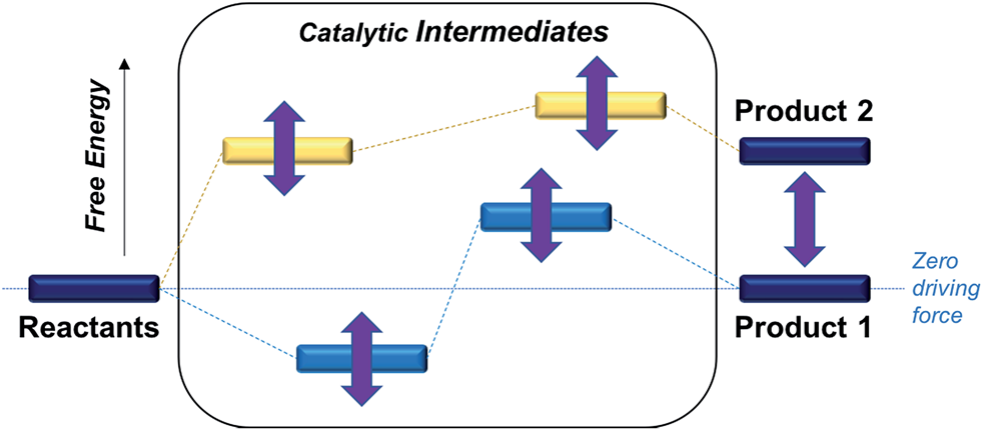
Catalyst design strategies.

In order to optimize catalytic activity, our two main strategies involve (a) establishing the relative free energies of the stoichiometric products for a given proton donor and (b) minimizing the free energy corrugation of the catalytic intermediates relative to zero driving force as illustrated in Scheme 1. The latter strategy is central to achieving the desired catalytic activity as intermediates that are high energy or deep trap states in the catalytic pathway will deactivate the catalysts. While the relative rates of different processes will be ultimately governed by the relative heights of transition states, the first screen of catalysts described in this work aims to understand how free energy corrugations of key catalytic intermediates are determined by the transition metal and the ligand environment.

A significant recent development in transition metal-mediated catalysis has been the application of density functional theory-based computations to model the reactivity of transition metal complexes.^18^–^20^ DFT-based methods have proven to predict with reasonable accuracy redox potentials, p*K*_a_'s and ligand-exchange equilibria of transition metal complexes in aprotic polar solvents that are well described by simple polarizable continuum models.^18^,^21^–^24^ These methods have been employed to model *post-facto* the catalytic landscape in a single family of catalysts with relatively small perturbations to the ligand field.^25^–^27^ While the resulting changes in reactivity are extremely useful for the system under study, there is little insight offered into the reactivity of a larger set of complexes. Accelerating the screening of metal and ligand choices towards predicting the optimal catalyst candidate with such methods requires the identification of suitable thermodynamic and kinetic descriptors.

The free energy of the transition metal hydride intermediate relative to reactants, products and other intermediates, an important thermodynamic quantity that determines the driving force towards hydride transfer to CO_2_ and protons to produce HCO_2_^–^ and H_2_ respectively, has been well studied as an important descriptor of catalytic activity.^28^–^31^ For instance, we previously showed, using experimentally calibrated DFT, how specific ligand environments change the free energy of CO_2_ insertion into a ruthenium hydride complex.^24^ The hydride complex we were studying despite being an active transfer hydrogenation catalyst for ketones^32^ was not found to be a catalyst for CO_2_ reduction due to product inhibition. In another case, a cyclopentadienyl Ru complex^33^ was predicted by using DFT to have near ergoneutral CO_2_ insertion into the corresponding Ru-H intermediate. However, CO_2_ coordinated to the singly reduced Ru-center prior to any hydride formation resulting in a Ru-bound CO intermediate that was a trap even after a third electrochemical reduction preventing turnover at reasonable potentials. This example highlighted the need to model the energetics of off-path intermediates during catalyst design. While in certain cases, the metal-bound CO can be labilized with a third reduction,^34^ the effect of the metal or the nature of reduction (ligand *vs.* metal-centered) on the dissociation is not well understood. Perhaps the most well-studied metal hydride complex for CO_2_ reduction is the [Ru(tpy)(bpy)H]^+^ complex (tpy = 2,2*′*,6,6*′*-terpyridine, bpy = 2,2*′*-bipyridine) by Creutz and others.^28^,^35^–^37^ While experimental as well as computational studies have unequivocally established that the hydride donating ability of this complex is well suited for ergoneutral CO_2_ insertion to produce HCO_2_^–^, under electrocatalytic conditions, CO is the major product.^38^ Therefore, the intermediates leading to CO production thermodynamically and kinetically compete with metal hydride chemistry, motivating the need for computational catalyst design approaches to go beyond hydricity as a single descriptor.

In this work, we explore a diverse library of transition metals and ligands and show the broader utilization of DFT in the catalyst design process. We compute the standard state free energies of the metal hydride and metal-bound CO intermediates relative to the stoichiometric reactants and products for metal complexes with diverse ligand environments around three earth-abundant d^6^ transition metal ions (Mn(i), Fe(ii) and Co(iii)), to screen for optimal metal–ligand combinations. This coarse level of screening based on just two standard state thermodynamic descriptors, using the BP86 density functional,^33^,^39^ weeds out bad candidates effectively and identifies two bipyridines (bpy) and a pyridine (py) ligand around Fe(ii) as promising candidates among the 36 complexes studied. Fortuitously the two redox-active bipyridyl units are predicted to provide optimal reduction potentials for accumulating the two electrons needed to form the hydride. Because the simple [Fe^II^(bpy)_2_(py)(CH_3_CN)]^2+^ complex would not be experimentally stable due to ligand scrambling at ambient temperatures, a pentadentate iron complex, [Fe(bpy2PYMe)S]^2+^ (bpy2PYMe = 1-(2-pyridyl)-1-bis-(6-2,2′-bipyridyl)ethane, S = CH_3_CN), reported previously by Long *et al.*^40^ was synthesized. [Fe(bpy2PYMe)S]^2+^ was indeed found to electrocatalytically reduce protons as well as CO_2_ at modest overpotentials with no catalyst trapping by CO. While the calculated reduction potentials and the free energies of the metal hydride and carbonyl intermediates are validated by the experimental results, the binding energy of CO_2_ to the singly reduced Fe complex was found to be energetically favorable in contrast to the predictions, resulting in the uphill formation of CO instead of the thermodynamically favored HCO_2_^–^. This work, therefore, provides an efficient first step of catalyst design, by computationally screening against bad candidates *via* the mapping of free energies of key intermediates in the catalytic pathway. More work is needed to further narrow the candidate space through better modelling of transition metal–CO_2_ interactions as a function of the redox state of the transition metal complex. To the best of our knowledge, this is the first reported application of DFT for the *a priori* design of molecular electrocatalysts.

## Results and discussion

### Thermodynamics of the stoichiometric reactions

For the two-electron reduction of CO_2_ to HCO_2_^–^ and of protons to H_2_ in the standard states of reactants and products, the relative driving force in acetonitrile as a function of the p*K*_a_ of the stoichiometric proton donor, HA, is shown in eqn (4). They are based on the thermochemical analyses shown in Table S1.†
4
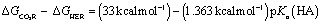



Given the difference in proton stoichiometry between eqn (1) and (2), plotting 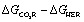 *versus* the p*K*_a_ of the proton donor HA in acetonitrile yields a straight line that crosses zero at a p*K*_a_ of *ca.* 24 (Fig. S1†). With weaker acids in acetonitrile (p*K*_a_ > 24) there will therefore be a thermodynamic bias for CO_2_ reduction to HCO_2_^–^*versus* proton reduction.^41^ For example, phenol (p*K*_a_ = 29.14)^42^ should disfavor H_2_ while acetic acid (p*K*_a_ = 23)^42^ should favor H_2_.

Under weakly acidic conditions, the two-electron, one-proton reduction of CO_2_ to CO produces bicarbonate (eqn (3)). Due to the lack of experimental free energies for this reaction in acetonitrile, we estimate an upper bound based on the two-proton reduction of CO_2_ to CO and water (eqn S1†). We estimate this latter process (eqn S1†) to be roughly uphill by *ca.* 4.8 kcal mol^–1^ relative to H_2_ production under water levels of ∼1 mM in acetonitrile (eqn S2†), in agreement with reported values.^43^

### Catalytic intermediates in the two-electron reductions

**Scheme 2:**
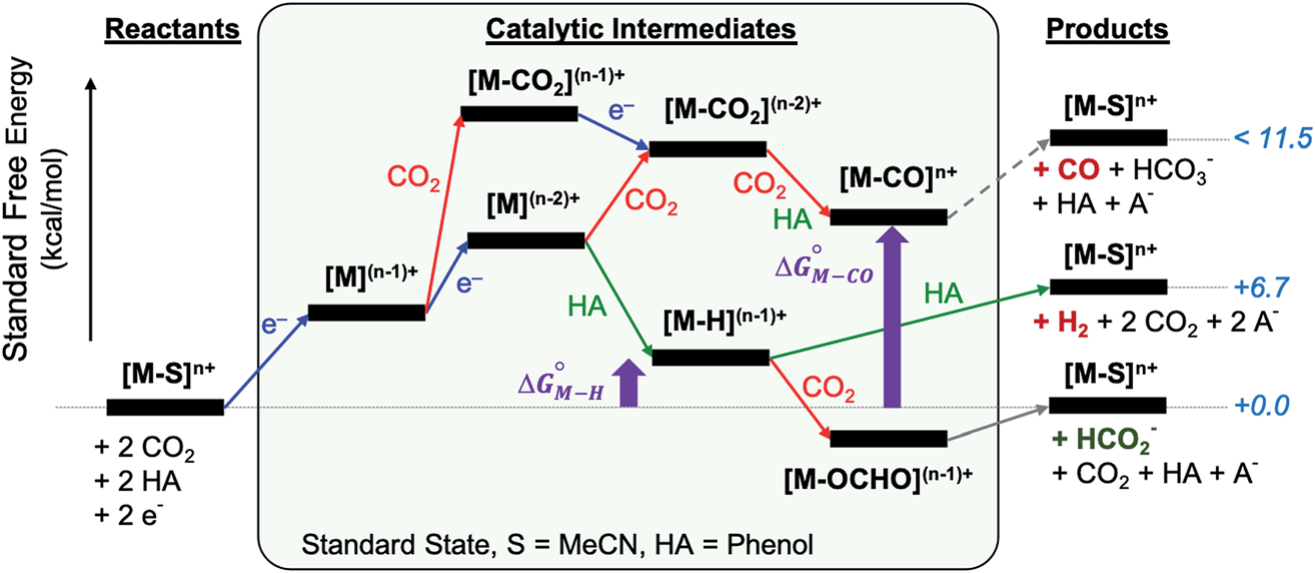
Two-electron reduction pathways for a generic transition metal electrocatalyst **[M–S]*^n^*^+^**, HA = proton donor. The free energies of catalytic intermediates are chosen arbitrarily.

The reactants, products and catalytic intermediates involved in the two-electron reduction of CO_2_ and protons as expected from chemical precedent are shown in Scheme 2, mediated by a generic transition metal complex **[M–S]*^n^*^+^**, where M is a transition metal in a six-coordinate ligand environment in which S is a labile solvent ligand (CH_3_CN in this case). The chemical potential of electrons is set to a value of –1.6 V *vs.* Fc^+^/Fc with phenol as the proton donor that makes CO_2_ and HCO_2_^–^ ergoneutral in the standard state, H_2_ at a relative free energy of 6.7 kcal mol^–1^ (eqn (4)), and CO with an upper bound of *ca.* 11.5 kcal mol^–1^ (see the ESI†).^44^

Two-electron reduction of the metal complex **[M–S]*^n^*^+^** with the dissociation of S is followed by protonation at the metal center to form the metal hydride intermediate. CO_2_ insertion into the metal hydride bond leads to HCO_2_^–^ while direct protonation of the metal hydride yields H_2_. Additionally, the singly or doubly reduced metal complex, **[M]^(^*^n^*^–1)+^** and **[M]^(^*^n^*^–2)+^** can directly bind CO_2_ at the metal center^33^ subsequently leading to the potential release of CO *via* an **[M–CO]*^n^*^+^** intermediate.

### *In silico* screening of catalyst candidates

**Scheme 3:**
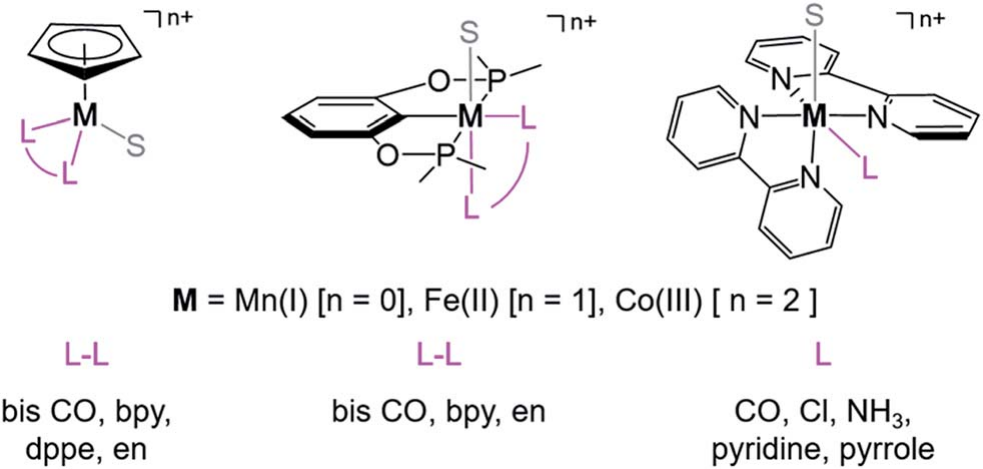
Families of d^6^ transition metal complexes screened (S = CH_3_CN, bpy = 2,2′-bipyridine, dppe = bis(diphenylphosphino) ethane, and en = 1,2-ethylene diamine).

Within this class of reactivity, we first calculate two key thermodynamic quantities, *viz.* the free energy of the metal hydride 
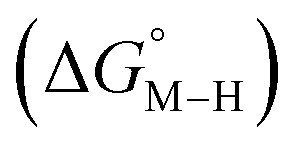
 and the metal carbonyl intermediates 
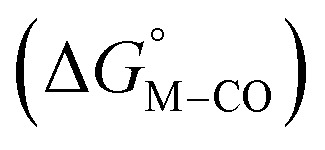
 relative to the thermodynamically favorably product with weak proton donors, HCO_2_^–^. Given the same proton stoichiometry in eqn (1) and (3), the comparison of the two thermodynamic quantities 
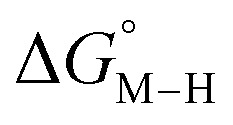
 and 
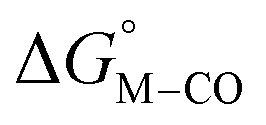
 relative to HCO_2_^–^ is independent of the choice of the proton donor, which will merely shift the relative energies of the stoichiometric products (Table S3†). Scheme 3 lists the families of complexes we chose for a comparison of their reactivity within our design framework. They encompass d^6^ earth-abundant metal ions (Mn(i), Fe(ii) and Co(iii)) and a diverse set of strong field ligands including polypyridines, phosphines, amines and carbonyls to enhance metal–ligand binding.

**Fig. 1:**
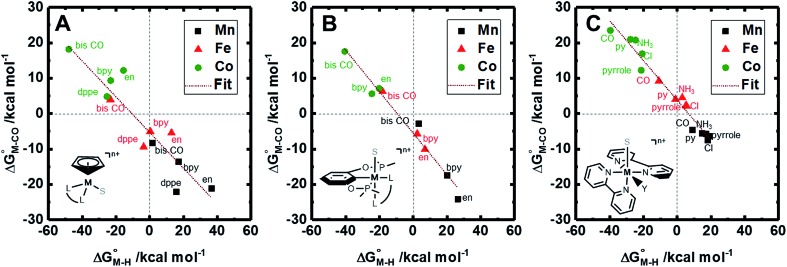
2D plot of the calculated values of 
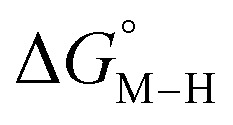
 and 
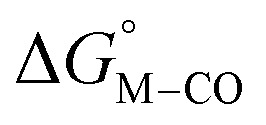
 (in kcal mol^–1^ units) for the d^6^ transition metal complexes listed in Scheme 3. Level of theory: BP86/LANL2DZ(M)/6-31+G*(C,H,N,O,P,Cl)/SMD(MeCN).

The two thermodynamic quantities 
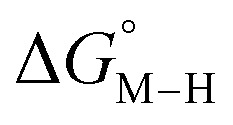
 and 
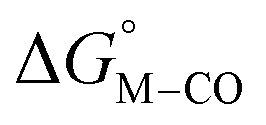
 relative to HCO_2_^–^ are plotted *versus* each other in Fig. 1 for all the complexes studied. These values are independent of the choice of the proton donor due to the equal proton stoichiometry in eqn (1) and (3). For selective and ergoneutral HCO_2_^–^ production, the optimal value of 
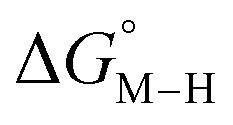
 is zero and 
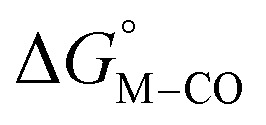
 is greater than zero, and *vice versa* for CO production. 
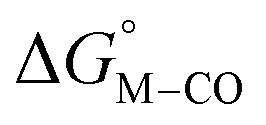
 values greater than 10 kcal mol^–1^, for example, would inhibit the formation of **[M–CO]*^n^*^+^** at room temperature.^45^

First, independent of the choice of metal, it is evident that all the data points fall roughly on a straight line for the cyclopentadienyl, pincer and bis-bipyridine frameworks. The slopes of the linear fits are all about –0.5, meaning that as the free energy of **[M–CO]*^n^*^+^** decreases and the free energy of **[M–H]^(^*^n^*^–1)+^** goes up. This is reasonable as an increase in electron density at the metal center is expected to increase the **[M–H]^(^*^n^*^–1)+^** free energy but lower the **[M–CO]*^n^*^+^** free energy due to increased back-bonding into the π* orbital of the carbonyl ligand. For the same metal, however, changing the ligands causes minor deviations from the best fit line, presumably due to different extents of sigma and pi interactions in these complexes. Second, all the Co complexes are clustered in the upper-left quadrant of the plot while all the Mn complexes are clustered in the lower-right quadrant across all the families of ligands. This suggests that while CO does not thermodynamically trap the Co(iii) complexes, the corresponding hydrides are all very stable relative to HCO_2_^–^ and therefore unreactive towards CO_2_. Conversely, in the Mn(i) systems, the hydrides lie higher than HCO_2_^–^ in relative energy, and therefore will react with CO_2_, but the corresponding carbonyl intermediates are very stable potentially leading to catalytic trap states. It is evident that of the three metals, the complexes of Fe are the best potential catalyst candidates as their 
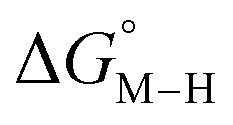
 and 
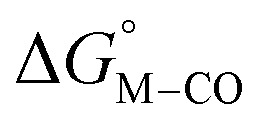
 values lie closest to the origin.

The slopes of the fits in all three plots are roughly the same but the *y*-intercepts vary. Notice that changing the overall charge only moves you along the best fit line. In the cyclopentadienyl and pincer complexes (Fig. 1A and B), for example, the *y*-intercept is negative (*ca.* –5 kcal mol^–1^) suggesting that **[M–CO]*^n^*^+^** will be dominant irrespective of the other ligands. The bis-bipyridine family of complexes, on the other hand, have a positive *y*-intercept of *ca.* 5 kcal mol^–1^ (Fig. 1C), resulting in a *ca.* 10 kcal mol^–1^ spread, which is greater than the *ca.* 5 kcal mol^–1^ error associated with such calculations. The bis-bipyridine iron complex with L = pyridine (
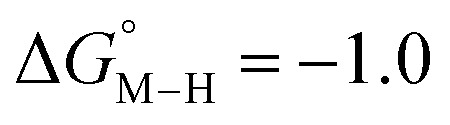
, 
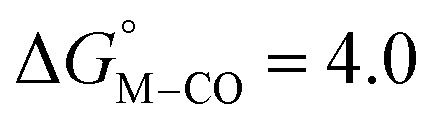
) is therefore an interesting candidate to test the predictions for ergoneutral metal hydride formation and the lack of catalyst trapping by CO, within an error of ±5 kcal mol^–1^ in the calculations (*vide infra*).

While the exact origin of the different intercept in the case of the bis-bipyridine family compared to the cyclopentadienyl and pincer families is unclear, we hypothesize that having a high degree of pyridyl ligation could play a role, presumably from a good balance between the σ-donating and π-accepting ability of pyridyl ligands. The former is responsible for conferring nucleophilicity on the metal center leading to a higher 
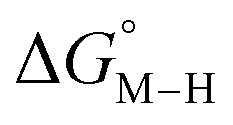
, and the latter for destabilizing **[M–CO]*^n^*^+^** through competition for π-back donation from the metal center.

**Fig. 2:**
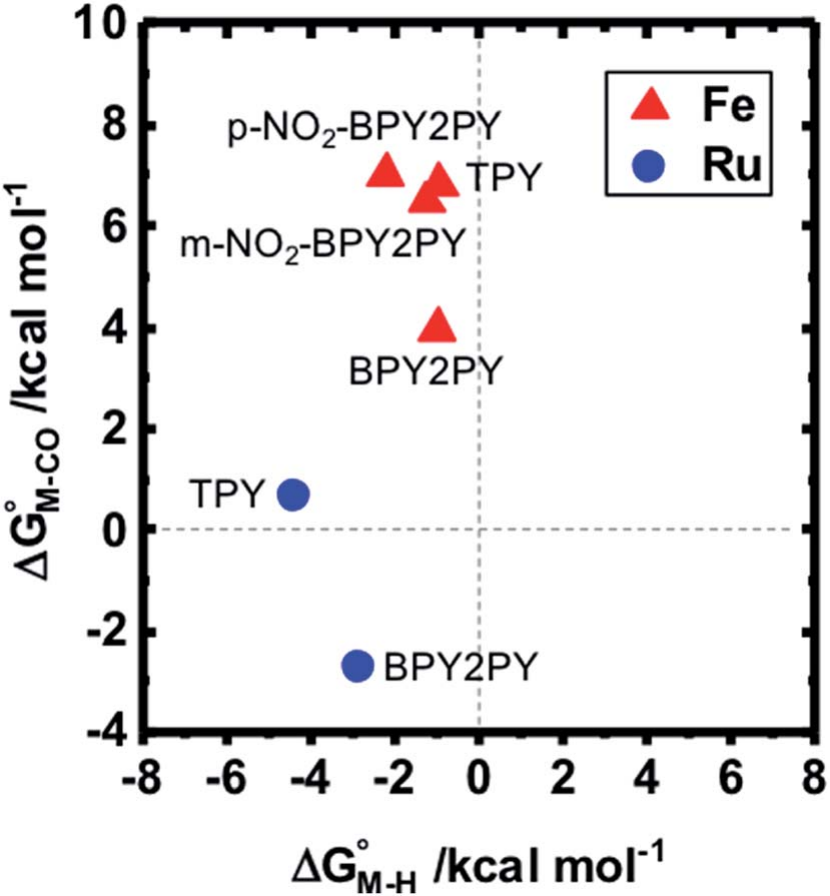
Effect of conjugation and substitution of the pyridine ring on 
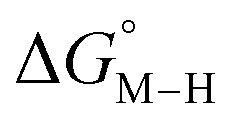
 and 
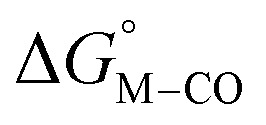
 in the [M(bpy)_2_(py)]^2+^ system (M = Fe, Ru).

To further probe this hypothesis, we modeled the effect of increasing the π-accepting ability of the pyridyl ring on 
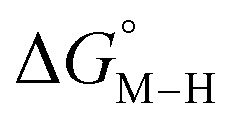
 and 
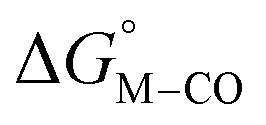
 for the [Fe(bpy)_2_(py)S]^2+^ system (Fig. 2). Firstly, conjugating the pyridine with one of the bipyridine ligands to make a terpyridine (tpy) ligand reduces the pyridine to Fe-CO dihedral angle (C-N-Fe-C) from 30° to zero, and aligns the π* orbital of the pyridine with the Fe-CO axis, which destabilizes the carbonyl ligand through competition for back-bonding. Consistent with this prediction, 
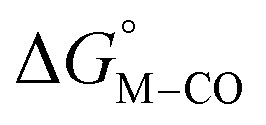
 for the [Fe(bpy)(tpy)]^2+^ system increases by *ca.* 3 kcal mol^–1^ with almost no effect on 
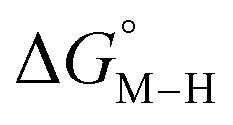
. Secondly, substitution at the para-position of the pyridine ring with a nitro group has a similar effect on 
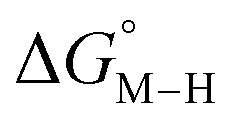
 and 
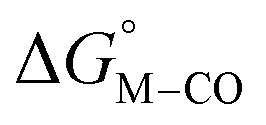
. Consistent with the known trapping of the [Ru(bpy)(tpy)]^2+^ system by CO,^38^ the Ru analogs have much lower values of 
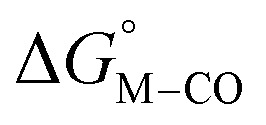
 owing to the greater back-bonding ability of Ru compared to Fe. While the π-accepting nature of the ligand trans to CO in a metal complex is regularly invoked as a determinant for thermodynamic stability (the trans influence), our results highlight a substantial cis influence on the energy of the carbonyl (but not of the hydride), similar to effects we have seen previously.^24^

### Electrocatalytic activity of [Fe(bpy2PYMe)(CH_3_CN)]^2+^

**Scheme 4:**
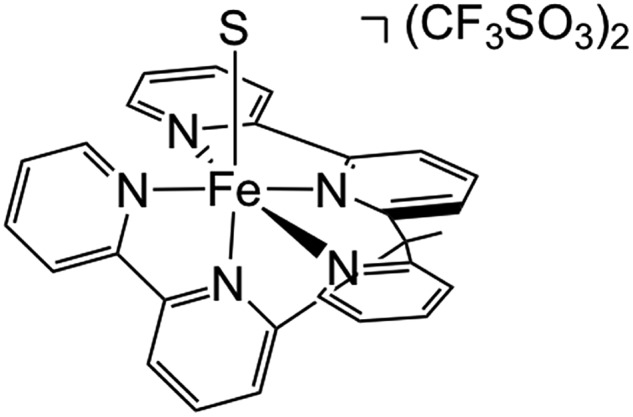
Structure of [Fe(bpy2PYMe)S](CF_3_SO_3_)_2_, where bpy2PYMe = 1-(2-pyridyl)-1-(6-2,2′- bipyridyl)ethane and S = CH_3_CN.

In order to experimentally test these predictions, we require a pentadentate ligand to prevent ligand scrambling. Long *et al.* recently reported the synthesis and characterization of the pentadentate ligand ‘bpy2PYMe’ (Scheme 4).^40^ This ligand closely matches the desired ligand environment in Fig. 1C for iron (L = pyridine), and therefore we synthesized and studied the electrocatalytic activity of the complex [Fe(bpy2PYMe)(CH_3_CN)]^2+^.

**Fig. 3:**
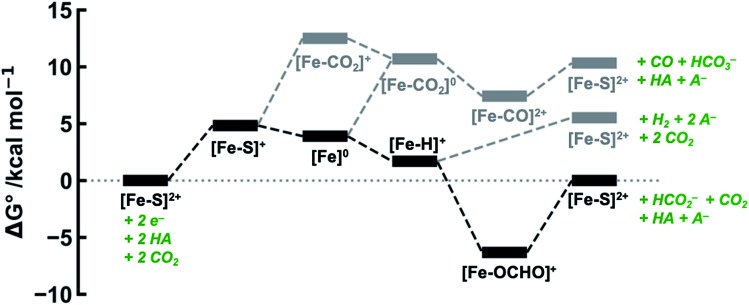
DFT-calculated energy level diagram (standard state) for CO_2_ reduction to HCO_2_^–^ (in black) and CO (in grey), and proton reduction to H_2_ (in grey), mediated by [Fe(bpy2PYMe)(CH_3_CN)]^2+^, with phenol as the proton donor.

The calculated free energies of the catalytic intermediates for the different two-electron reduction processes (eqn (1)–(3)) mediated by the complex [Fe(bpy2PYMe)(CH_3_CN)]^2+^ are shown in Fig. 3, with phenol as the stoichiometric proton donor. In addition to possessing low corrugations with respect to 
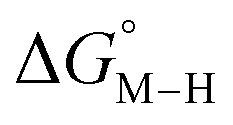
 and 
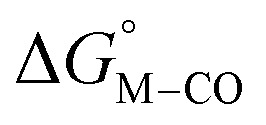
, *viz.* 1.7 kcal mol^–1^ and 7.4 kcal mol^–1^ respectively, the most endergonic on-path intermediates for this system are within 5 kcal mol^–1^. This complex is therefore likely to avoid high thermodynamic barriers and operate close to the standard potential for the reduction reactions. We note that in Fig. 3, the metal formate intermediate is predicted to be a product inhibitor. Previous work suggested that formate can be labilized with the modification of the solvent composition to provide H-bond stabilization.^12^,^24^

**Fig. 4:**
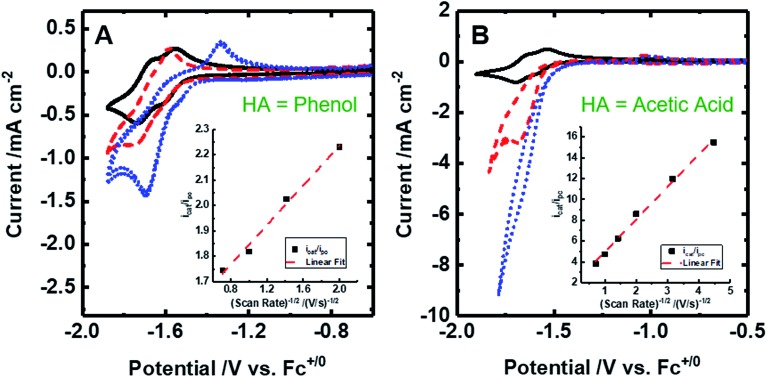
Cyclic voltammograms of 2 mM [Fe(bpy2PYMe)(CH_3_CN)]^2+^ at 100 mV s. (A) [Fe(bpy2PYMe)(CH_3_CN)]^2+^ only (black solid), with the addition of 0.3 M phenol (red dashed) followed by satd. CO_2_ (*ca.* 0.28 M, blue dotted). (B) [Fe(bpy2PYMe)(CH_3_CN)]^2+^ only (black solid), with the addition of 0.3 M acetic acid (red dashed) or 0.5 M acetic acid (blue dotted). Insets show the linear fit of the ratio of the current with the substrate (*i*_cat_) to the peak current in the absence of any substrate (*i*_pc_) at *ca.* –1.75 V as a function of the inverse square root of the scan rate (eqn (5)).

The cyclic voltammogram of [Fe(bpy2PYMe)(CH_3_CN)]^2+^ in acetonitrile is shown in Fig. 4. The complex undergoes two one-electron reductions at –1.55 V and –1.66 V *vs.* Cp_2_Fe^+/0^ (Cp_2_Fe = ferrocene). The experimental redox potentials match up perfectly with those reported by Long and coworkers.^40^,^46^ Moreover, the calculated redox potentials (Table 1) are within about 100 mV of these measured values. For further validation, CO gas was passed through a CD_3_CN solution of [Fe(bpy2PYMe)(CH_3_CN)]^2+^ in a J-Young NMR tube for *ca.* 30 minutes. No changes in the ^1^H NMR spectrum of [Fe(bpy2PYMe)(CH_3_CN)]^2+^ were observed, suggesting that the corresponding [Fe(bpy2PYMe)(CO)]^2+^ complex is in negligible concentration, which is in agreement with our calculations (Fig. 3).

**Table 1:** Calculated and measured redox potentials of [Fe(bpy2PYMe)(CH_3_CN)]^2+^

	E^°^/V *vs.* Cp_2_Fe^+/0^	
	Calculated	Experimental
1st reduction	–1.46	–1.55
2nd reduction	–1.65	–1.66

Upon the addition of 0.3 M phenol (p*K*_a_ = 29.14 in acetonitrile^42^) to the CV solution containing 2 mM [Fe(bpy2PYMe)(CH_3_CN)]^2+^, there is a slight enhancement in the current at the second reduction potential suggesting that H_2_ evolution is taking place through the formation of a metal hydride and subsequent protonation. Saturating this solution with CO_2_ (*ca.* 0.28 M)^47^ results in a higher (*ca.* 2.5-fold) current enhancement near the second reduction potential (*ca.* –1.66 V *vs.* Cp_2_Fe^+/0^), indicating that CO_2_ is a substrate for an electrocatalytic process mediated by [Fe(bpy2PYMe)(CH_3_CN)]^2+^. Assuming a 2 mM concentration of phenoxide and a 300 mM concentration of phenol^48^ we calculate that this electrocatalytic process is occurring at an overpotential of *ca.* 200 mV, from eqn (4) with p*K*_a_ = 29.14 for phenol. Scan rate dependent ratios of the current in the presence of CO_2_ to the peak current in its absence (eqn (5), Fig. 4A (inset) and S2†) yield a pseudo-first order rate constant of *ca.* 2.4 s^–1^ for the two-electron electrocatalytic process involving the reduction of CO_2_. Unexpectedly, there is also a positive shift of the onset potential of the first peak in the CV with no corresponding current increase at this potential. Upon the addition of CO_2_ in the absence of phenol (Fig. S3†) a similar positive shift in the onset potential is observed in the CV. Both these results suggest that there is energetically favorable CO_2_ binding accompanying the first electrochemical reduction of [Fe(bpy2PYMe)(CH_3_CN)]^2+^, giving rise to the positive shift in the onset in the presence of CO_2_. On this basis, we assign the oxidative feature around *ca.* –1.35 V *vs.* Cp_2_Fe^+/0^ to the [Fe(bpy2PYMe)(CO_2_)]^2+/+^ couple. Fig. S4† shows that the experimental data can be approximately simulated by CO_2_ binding and a small amount of reductive deoxygenation at extremely negative potentials, presumably due to carbonate formation.^33^ This unexpected result of favorable CO_2_ binding to the singly reduced intermediate [Fe(bpy2PYMe)]^+^, contrary to the DFT-based predictions in Fig. 3, is discussed below.

The effect of proton strength on the electrocatalytic behavior of [Fe(bpy2PYMe)(CH_3_CN)]^2+^ was explored with acetic acid (p*K*_a_ = 23.51 in acetonitrile)^42^ as the proton donor instead of phenol. In the cyclic voltammograms (Fig. 4B), the electrocatalytic currents near the second reduction potential of [Fe(bpy2PYMe)(CH_3_CN)]^2+^ are over three times greater than with phenol. However, the overpotential, as compared to the phenol case, is about 200 mV higher, as 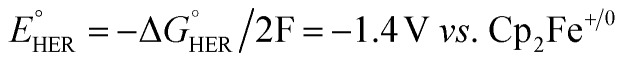 in the standard state (Table S1†). With 0.5 M acetic acid, a pseudo-first order rate constant greater than 150 s^–1^ (the actual rate is limited by the cell resistance) was estimated from scan-rate dependent CVs (Fig. S2†). Upon the addition of CO_2_ (*ca.* 0.28 M) there is a greater current enhancement and positive shift of the onset potential (Fig. S5†), qualitatively similar to what was observed with phenol. Bulk electrolysis of a 2 mM solution of [Fe(bpy2PYMe)(CH_3_CN)]^2+^ was first performed with 0.3 M phenol and 0.1 M KPF_6_ as the supporting electrolyte in CO_2_-saturated CD_3_CN (3 mL solution) at a potential of –1.65 V *vs.* Cp_2_Fe^+/0^ near the onset of the second reduction wave (Fig. 4A, see the Experimental details). Decomposition of the Fe species in solution preceded any appreciable turnover, and therefore no products were detected. The electrolysis experiment was then repeated with 0.3 M acetic acid as the proton donor instead of phenol. A 1 mL aliquot of the headspace was injected into a gas chromatograph to detect and quantify gaseous products (Fig. S6†). Based on the calibration of the GC peak areas with 1% gas standards, the amounts of gaseous products were estimated to be 8.1 × 10^–6^ moles of CO and 2.6 × 10^–5^ moles of H_2_. They correspond to 9% and 30% of the total charge passed in the electrolysis experiment respectively for two-electron stoichiometry, suggesting that other reduced products are present. In the ^1^H NMR spectrum of the electrolyte solution, the peak corresponding to HCO_2_^–^ at *ca.* 8.2 ppm^49^ was not observed. Decomposition of the Fe species in solution prevented comprehensive analyses of the liquid phase products. With acetic acid as the proton donor, H_2_ and HCO_2_^–^ are predicted to be roughly ergoneutral in the standard state (Fig. S1†). However, under the electrolysis conditions, there is a *ca.* 200 mV extra driving force towards H_2_ from the higher proton activity due to the absence of a stoichiometric conjugate base, as well as from homoconjugation effects.^42^,^48^ These effects along with the second reduction potential of [Fe(bpy2PYMe)(CH_3_CN)]^2+^, –1.66 V *vs.* Cp_2_Fe^+/0^ (and the electrolysis potential), being *ca.* 400 mV more negative than the potential for H_2_ production under the electrolysis conditions (*vide supra*), together explain the observed H_2_ in the electrolysis.

We hypothesize that the lack of the thermodynamically favored product, HCO_2_^–^, under the electrolysis conditions, is due to the rate of CO_2_ insertion into the metal hydride being significantly slower than the rate of protonation of the metal hydride. CO_2_ insertion into hydrides is typically a slow process (*ca.* 1.82 × 10^–2^ M^–1^ s^–1^ for [Ru(tpy)(bpy)H]^+^ at 298 K in acetonitrile^50^). The incorporation of proton-directing groups into the ligand backbone to accelerate the rate of CO_2_ insertion into the metal hydride, as we have explored in previous work,^24^ would favor the HCO_2_^–^ pathway.

The production of CO as a minor product indicates direct binding of CO_2_ during the electrochemical reduction of [Fe(bpy2PYMe)(CH_3_CN)]^2+^. The direct CO_2_ binding pathway, evidenced by the positive shift of the first reduction wave in the CV in Fig. S3,† competes with hydride formation (Scheme 2). However, DFT calculations at the current level of theory suggest that the binding of CO_2_ after the first reduction is uphill by *ca.* 7 kcal mol^–1^ (Fig. 3). The same calculation using the hybrid functional B3LYP instead of BP86 suggests that the CO_2_ binding step after the first reduction is downhill by about 4 kcal mol^–1^. It is, therefore, possible that while BP86 captures redox potentials and the free energy of key intermediates such as the metal hydrides and metal carbonyls with reasonable accuracy, it underestimates the free energy of CO_2_ binding to the reduced Fe-complexes. Therefore, a catalyst search algorithm that employs the free energies of the metal hydride and carbonyl intermediates alone (Fig. 1) will effectively weed out bad candidates based on these two descriptors. However, within this narrowed space of candidates, favorable CO_2_ binding to the metal center could lead to CO production, as evidenced in the case of [Fe(bpy2PYMe)(CH_3_CN)]^2+^. This motivates further refinement and experimental validation of DFT methods to adequately model the thermodynamics of CO_2_ binding to reduced metal complexes, in order to map the free energies of the whole range of intermediates in a catalytic pathway.

## Conclusions

In this work we have shown how the calculation of two thermodynamic parameters, *viz.* the relative free energies of the metal hydride and the CO-bound intermediates by using DFT streamlines the search for an appropriate transition metal and ligand environment for catalyzing the multi-electron electrochemical reduction of CO_2_ or protons at low driving forces. We applied this catalyst screening approach on a library of common ligands around three earth-abundant d^6^ metal ions and tested the *in silico* predictions *via* the synthesis and electrochemical studies of an Fe-based complex, predicted to avoid trapping by CO, form a metal hydride of appropriate free energy, and possess optimal redox potentials. These predictions were validated, and we found the iron-based electrocatalyst to be active towards CO_2_ reduction as well as H_2_ production at the predicted potentials. The model, however, underestimated the free energy of CO_2_ binding to the reduced metal complex. We highlight the need for refinements to the DFT-methods to adequately capture the free energy of this latter step that leads to CO production, in order to further narrow the candidate space. This work is, to the best of our knowledge, the first application of DFT as a screen for molecular electrocatalysts across diverse molecular environments and paves the way forward for computations to take the lead in identifying promising catalysts for electrocatalytic transformations.

## Experimental details

The ligand bpy2PYMe and its Fe(ii) complex were prepared as per reported protocols.^40^^1^H NMR of bpy2PYMe (CDCl_3_, 400 MHz, ppm): *δ* 8.62 (3H, d), 8.29 (2H, d), 8.22 (2H, d), 7.73 (2H, t), 7.68 (2H, d), 7.57 (1H, t), 7.24–7.20 (4H, m), 7.13 (2H, m), 2.53 (3H, s). bpy2PYMe was then stirred with Fe(CF_3_SO_3_)_2_ for 24 hours at room temperature in acetonitrile, followed by filtration through a Celite plug. The solvent was removed, and the crude residue was recrystallized with CH_3_CN–diethyl ether. The resulting dark-red/black crystals were filtered and dried in a vacuum oven at 50 °C for three days. ^1^H NMR (CD_3_CN, 500 MHz, ppm): *δ* 9.24 (1H), 8.53 (3H), 8.32 (2H, d), 8.13 (2H, d), 8.03 (2H), 7.97 (2H, d), 7.87 (2H, t), 7.38 (1H), 7.30 (1H, t), 7.21 (1H, d), 6.89 (1H, d), 2.80 (3H, s).

All electrochemical experiments were performed inside a N_2_ glovebox fitted with a CO_2_ feedthrough using a SP-200 potentiostat from Bio-Logic Co. For the cyclic voltammetry experiments, a glassy carbon electrode (3 mm diameter) from BASi Inc. was used in combination with a Ag/AgNO_3_ (10 mM)/TBAPF_6_ (100 mM) reference electrode and a Pt wire counter electrode in a sealed cell. The pseudo-first-order rate constant *k*_cat_ for the catalytic waves in Fig. 4 was determined using eqn (5)^12^ (ν is the scan rate).5
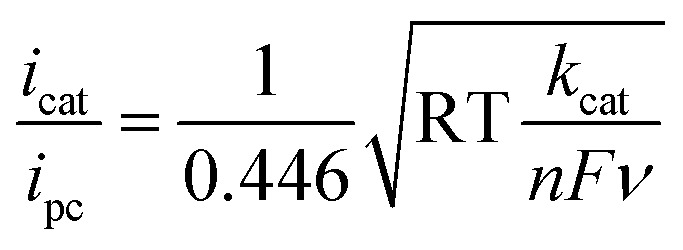



Bulk-electrolysis was performed in an ‘H-cell’ with two compartments, one for the working and reference electrodes and the other for the counter electrode, separated by a porous glass frit. The working electrode was a glassy carbon rod (BASi Inc.), and the reference and counter electrodes were the same as used in the cyclic voltammetry experiments. Both compartments were sealed with rubber septa and stirred vigorously during electrolysis. The entire process was carried out in a N_2_-filled glove box. Headspace product analyses were performed using a Shimadzu gas chromatograph fitted with a 100 μL sample injection loop, an FID, a TCD and a CarbonPLOT column with N_2_ carrier gas. Percent H_2_ and CO were determined with one-point calibration with a 1% gas standard purchased from Sigma-Aldrich Co.

## Computational details

All calculations based on Kohn–Sham density functional theory were performed using the Gaussian 09^51^ (Rev D. 01) software package. The BP86 (ref. 52 and 53) functional was used for all the calculations in combination with a double-zeta 6-31+G*^54^–^56^ basis set on all the p-block elements, and the LANL2DZ^57^ effective core potential on the transition metals. The relevant intermediates were first optimized in the gas phase followed by a harmonic analysis on the stationary point to obtain enthalpic, entropic and zero-point energy corrections to the electronic energy in the standard state, as implemented in Gaussian 09. The solvation energy was then determined with a single point calculation with a polarizable continuum model (SMD) for acetonitrile.^58^ Standard state corrections were made to the free energies to account for the change in going from 1 mol per 24.46 L (gas phase) to 1 M (solution phase). This level of theory provided excellent agreement with experimental values of the reduction potentials *versus* the ferrocene/ferrocenium couple in acetonitrile (eqn (6)–(8)), as well as hydricities for representative complexes in each family of complexes listed in Scheme 3.^37^,^59^,^60^

6 Ox^*n*+^ + Cp_2_Fe ⇌ Red^(*n*–1)+^ + Cp_2_Fe^+^

7
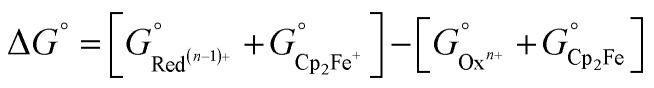

8
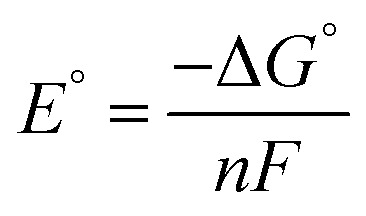



For all the complexes reported in this work, the lowest spin states (singlets for the even-electron intermediates and doublets for the odd-electron intermediates) were found to be the thermodynamically favorable states. This is expected for the local density functional BP86 given the choice of strong-field ligands.^20^ The B3LYP^52^,^61^–^63^ functional was employed in one instance for comparison of the CO_2_ binding energies to the singly reduced Fe complex. Simulations of the electrochemical data were performed with Digielch^64^ using a planar semi-infinite 1D diffusion model (see the ESI†).

## Conflicts of interest

The authors declare no competing financial interests.

## Supplementary Material

Supplementary information
