# Supplementary material for: Mapping free energy regimes in electrocatalytic reductions to screen transition metal-based catalysts
Source: Chem Sci. 2019 Jun 27;10(32):7649–58. doi: 10.1039/c9sc01766f (PMC6761863; doi:10.1039/c9sc01766f)
Supplement: Supplementary file 1 [file SC-010-C9SC01766F-s001.pdf]

Electronic Supplementary Material (ESI) for Chemical Science.
This journal is © The Royal Society of Chemistry 2019

Supporting Information for

MAPPING FREE ENERGY REGIMES IN ELECTROCATALYTIC REDUCTIONS TO SCREEN TRANSITION METAL-BASED CATALYSTS

Srinivasan Ramakrishnan, Ross Moretti, Christopher E. D. Chidsey*
Department of Chemistry, Stanford University, Stanford CA 94305

*chidsey@stanford.edu

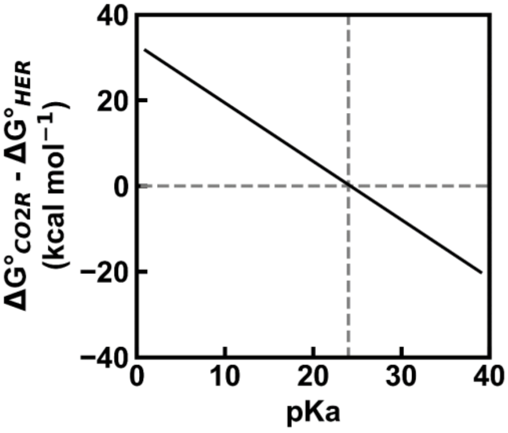

Figure S1. Relative driving forces for CO_2 reduction to formate versus proton reduction to H_2 in acetonitrile at standard state as a function of the pKa of the proton source (equations 5 and 6). Ferrocene is the stoichiometric electron donor. Dashed lines show the crossing point ($\Delta G^{\circ}_{CO2R} = \Delta G^{\circ}_{HER}$) at a pKa of ca. 24.

Table S1. Thermochemical cycle for the redox-potential calculation of the two-electron reduction of CO_2 to formate in acetonitrile with a generic proton source HA and ferrocene as the electron donor at standard state.

**Equation**	**Std. State Free Energy** (kcal/mol)
$H^- + CO_2 \rightleftharpoons HCO_2^-$	$-\Delta G^{\circ}_{H^-(HCO_2^-)} = -43$ [1]
$H_2 \rightleftharpoons H^+ + H^-$	$\Delta G^{\circ}_{H^-(H_2)} = 76$ [2]
$2H^+ + 2e^- \rightleftharpoons H_2$	$\Delta G^{\circ}_{H^+/H_2} = -2FE^{\circ}_{H^+/H_2} = 1.3$ [3]
$HA \rightleftharpoons H^+ + A^-$	$2.303\ RT\ pKa_{(HA)}$
$HA + CO_2 + 2e^- \rightleftharpoons HCO_2^- + A^-$	$34.3 + 2.303\ RT\ pKa_{(HA)}$

Table S2. Thermochemical cycle for CO production.

$$2\,HA + CO_2 + 2e^- \rightarrow CO + H_2O + 2\,A^- \quad \text{(S1)}$$

The two-proton reduction of CO_2 to CO and water (equation S1), can be deconvoluted into H_2 evolution and the reverse water-gas shift reaction:

$CO_2 + H_2 \rightleftharpoons CO + H_2O$	$\Delta G^\circ_{\text{RWGS}}$
$2\,HA + 2e^- \rightleftharpoons H_2 + 2\,A^-$	$\Delta G^\circ_{\text{HER}}$
$2\,HA + CO_2 + 2e^- \rightleftharpoons CO + H_2O\ (1\text{ mM}) + 2\,A^-$	$\Delta G^{\circ\prime}_{\text{CO}}$

At 298 K and 1 atm of H_2O, the $\Delta G^\circ_{\text{RWGS}}$ (reverse water-gas shift reaction) is ca. 6.8 kcal/mol.[4] However, note that unlike HER, CO production (equation S1) is favored by producing an additional species, water, that is present at very low concentration in the nominally dry acetonitrile used in this work. Assuming approximately 1 mM water, the modified free energy of the reaction with all other species still at standard state, $\Delta G^{\circ *}_{\text{RWGS}}$ is reduced to $6.8\text{ kcal/mol} + 0.593\text{ kcal/mol} * \ln[\frac{(p_{CO}/1\,atm)}{(p_{H2}/1\,atm)} * \frac{(1\,mM/40\,mM)}{(p_{CO2}/1\,atm)}]$ = 4.8 kcal/mol.

Therefore, from table S2: $\Delta G^{\circ *}_{\text{CO}} = \Delta G^\circ_{\text{HER}} + 4.8$ (in kcal/mol) (S2)

With phenol as the proton donor, $\Delta G^{\circ *}_{\text{CO}} = 6.7 + 4.8 = 11.5\text{ kcal/mol}$

Subtracting equation 3 from equation S1 yields the proton-transfer equilibrium for converting the proton donor HA and HCO_3^- to CO_2, H_2O and the conjugate base, A^-. Under weakly acidic conditions, it is probable that the one-proton CO production pathway (equation 3) might be thermodynamically more favorable than equation S1. We use the free energy of the dehydration pathway (equation S1) as an upper bound for the free energy of CO production.

Table S3. Thermochemical cycles for the calculation of ΔG°_{M-H} and ΔG°_{M-CO} relative to HCO_2^- for a proton donor HA.

$\textbf{[M-S]}^{n+} + \frac{1}{2}(HCO_2^- + A^-) \rightleftharpoons \textbf{[M]}^{(n-1)+} + \frac{1}{2}(CO_2 + HA) + S$
$\textbf{[M]}^{(n-1)+} + \frac{1}{2}(HCO_2^- + A^-) \rightleftharpoons \textbf{[M]}^{(n-2)+} + \frac{1}{2}(CO_2 + HA)$
$\textbf{[M]}^{(n-2)+} + HA \rightleftharpoons \textbf{[M-H]}^{(n-1)+} + A^-$
$\textbf{[M-S]}^{n+} + HCO_2^- \rightleftharpoons \textbf{[M-H]}^{(n-1)+} + CO_2 + S$

$\textbf{[M-S]}^{n+} + \frac{1}{2}(HCO_2^- + A^-) \rightleftharpoons \textbf{[M]}^{(n-1)+} + \frac{1}{2}(CO_2 + HA) + S$
$\textbf{[M]}^{(n-1)+} + \frac{1}{2}(HCO_2^- + A^-) \rightleftharpoons \textbf{[M]}^{(n-2)+} + \frac{1}{2}(CO_2 + HA)$
$\textbf{[M]}^{(n-2)+} + HA + 2\,CO_2 \rightleftharpoons \textbf{[M-CO]}^{n+} + HCO_3^- + A^-$
$\textbf{[M-S]}^{n+} + HCO_2^- + CO_2 \rightleftharpoons \textbf{[M-CO]}^{n+} + HCO_3^- + S$

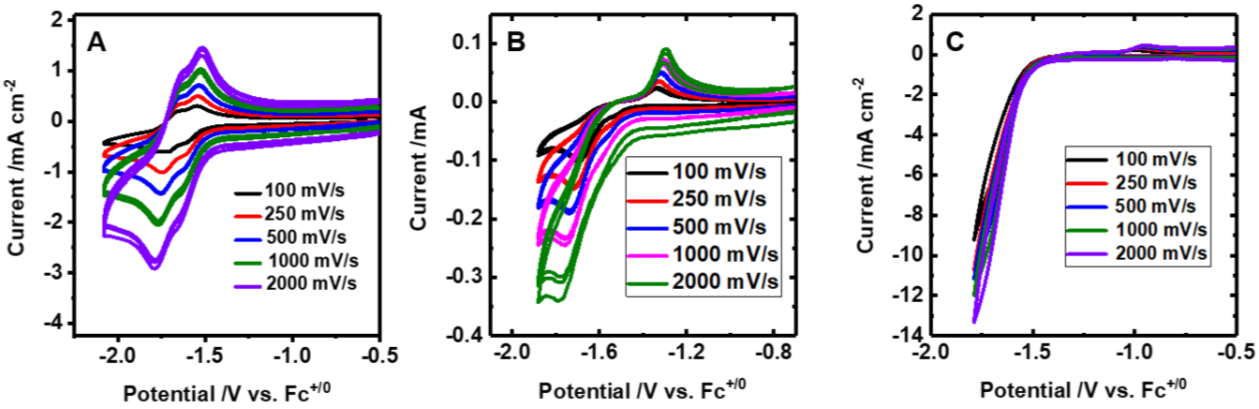

Figure S2. Scan-rate dependence of the CV of (A) 2 mM [Fe(bpy2PYMe)(CH_3CN)]$^{2+}$, (B) 2 mM [Fe(bpy2PYMe)(CH_3CN)]$^{2+}$ in the presence of ca. 0.28 M CO_2 and 0.3 M phenol, and (C) 2 mM [Fe(bpy2PYMe)(CH_3CN)]$^{2+}$ in the presence of ca. 0.28 M CO_2 and 0.5 M acetic acid, all in acetonitrile.

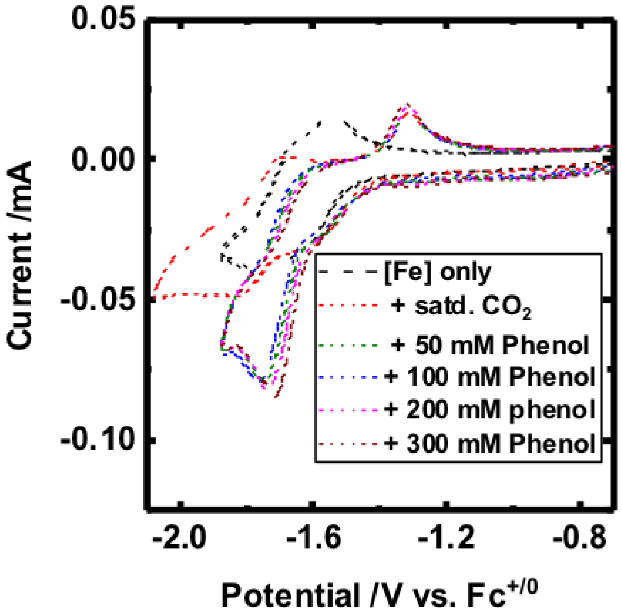

Figure S3. CV of (A) 2 mM [Fe(bpy2PYMe)(CH_3CN)]$^{2+}$, with the addition of ca. 0.28 M CO_2 and then incremental addition of phenol; scan rate = 100 mV/s.

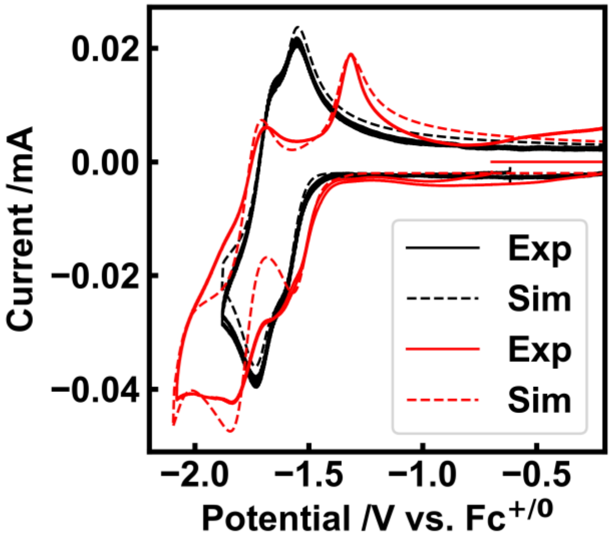

Figure S4. Experimental and simulated cyclic voltammograms (scan rate = 100 mV/s) of 2 mM $[Fe(bpy2PYMe)(CH_3CN)]^{2+}$ (in black), with the addition of ca. 0.28 M CO_2 (in red).

Simulation Parameters

All voltammograms were simulated with a planar semi-infinite 1D diffusion model in Digielch™, with the following input parameters: area of the electrode = 0.07 cm^2; R_u = 100 Ω; C_{dl} = 20 µF (to model large background charging current); temperature = 298 K; α (transfer coefficient) = 0.5; k_0 = 0.01 cm/s; $[Fe(bpy2PYMe)(CH_3CN)]$ abbr. as [Fe]. $c_{[Fe]}$ = 2 mM; $c_{[CO2]}$ = 0.28 M. Diffusion coefficients for the Fe-species: D = $4x10^{-6}$ cm^2s^{-1}, and for all other species D = $1x10^{-5}$ cm^2s^{-1}.

The experimental current was corrected by subtracting a background of 7 µA/V.

Table S4. Redox potentials employed in the Digielch™ simulations.

	$E^\circ([Fe]^{2+/+})$ V vs. $Fc^{+/0}$	$E^\circ([Fe]^{+/0})$ V vs. $Fc^{+/0}$	$E^\circ([Fe]^{0/-1})$ V vs. $Fc^{+/0}$
$[Fe(bpy2PYMe)(CH_3CN)]$	-1.58	-1.70	-
$[Fe(bpy2PYMe)(CO_2)]$	-1.35	-1.78	-2.10
[Fe(bpy2PYMe)(CO)]	-1.35	-1.75	-

Table S5. Equilibrium constants and rate constants for CO_2 binding and deoxygenation to CO, employed in the Digielch™ simulations.

	$[Fe]^{n+} + CO_2 \rightleftharpoons [Fe\text{-}CO_2]^{n+}$		$[Fe\text{-}CO_2]^{(n-2)+} + CO_2 \rightleftharpoons [Fe\text{-}CO]^{n+} + CO_3^{2-}$		$[Fe]^{n+} + CO \rightleftharpoons [Fe\text{-}CO]^{n+}$	
	K_{eq} /M^{-1}	k_f /$M^{-1}s^{-1}$	K_{eq}	k_f /$M^{-1}s^{-1}$	K_{eq} /M^{-1}	k_f /$M^{-1}s^{-1}$
n = 2	0.1	0.1	10.0	3.0	0.1	0.01
n = 1	1000	3000	4.7	30.0	771	100
n = 0	44.5	3000	-	-	-	-

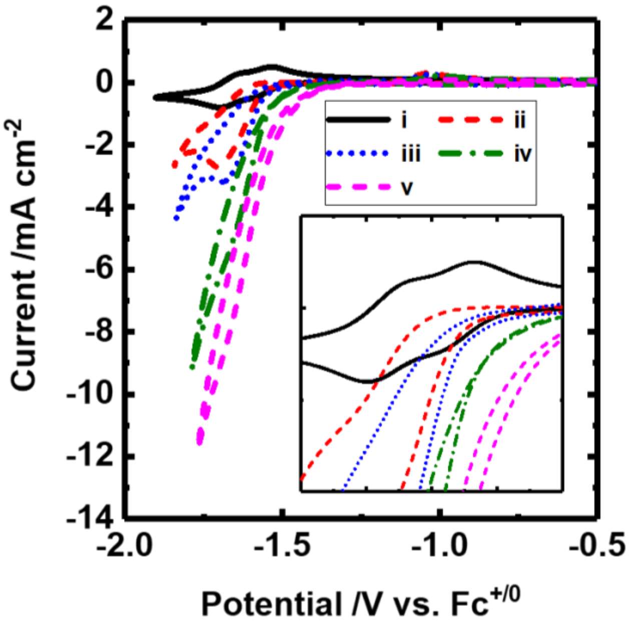

Figure S5. CV of (i) 2 mM $[Fe(bpy2PYMe)(CH_3CN)]^{2+}$ in acetonitrile at a scan rate of 100 mV/s, with the incremental addition of (ii) 0.1 M, (iii) 0.3 M, (iv) 0.5 M acetic acid, and (v) ca. 0.28 M CO_2. Inset is a magnification of the region where the positive shift of the onset potentials is clearly seen.

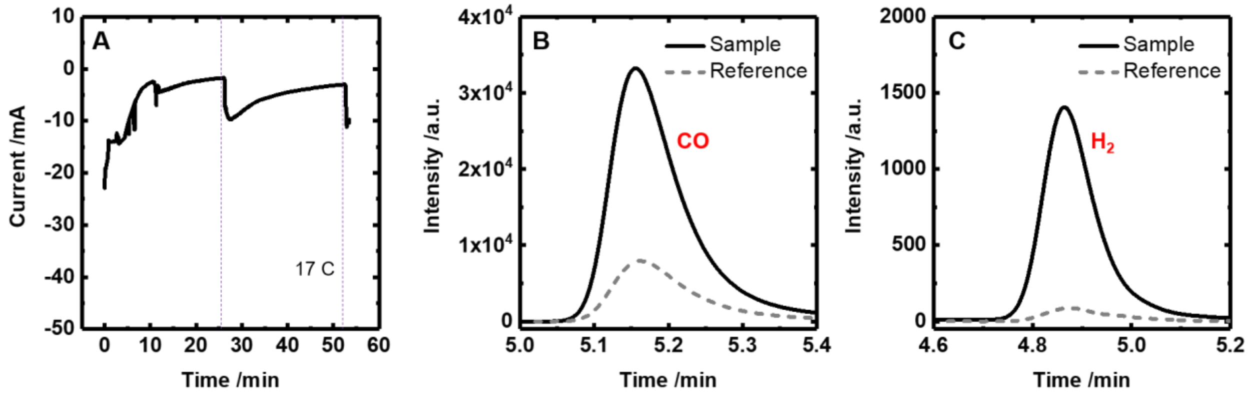

Figure S6. (A) Current vs. time plot of the bulk electrolysis experiment. Gas chromatographs of the headspace after 52 min of electrolysis for (B) CO and (C) H_2 production, based on comparison with a 1% reference gas standard.

References

1 J. T. Muckerman, P. Achord, C. Creutz, D. E. Polyansky and E. Fujita, *Proc. Natl. Acad. Sci.*, 2012, **109**, 15657–15662.

2 A. D. Wilson, A. J. M. Miller, D. L. Dubois, J. A. Labinger and J. E. Bercaw, *Inorg. Chem.*, 2010, **49**, 3918–3926.

3 J. A. S. Roberts and R. M. Bullock, *Inorg. Chem.*, 2013, **52**, 3823–3835.

4 E. Demirel and N. Azcan, *Proc. World Congr. Eng. Comput. Sci.*, 2012, **II**, 24–27.

Cartesian Coordinates of Intermediates

1. Cyclopentadienyl Systems: $[CpM(L\text{-}L)]^{n+}$

L-L = bis CO
M = Mn

$[Mn\text{-}S]^0$

	x	y	z
C	-1.25365200	-1.48287500	-1.16537200
C	-0.66259500	-2.06420700	0.00031100
C	-1.25424000	-1.48246800	1.16547200
C	-2.22303400	-0.51095100	0.71570800
C	-2.22267200	-0.51121400	-0.71642900
H	-1.01172600	-1.72228000	-2.20155300
H	0.14759900	-2.79596900	0.00065100
H	-1.01281400	-1.72148800	2.20185900
H	-2.85148100	0.11170500	1.35435400
H	-2.85079100	0.11122500	-1.35561200
Mn	-0.30060100	0.10541800	0.00000000
C	-0.19079400	1.33358700	-1.26507000
C	-0.19081200	1.33342500	1.26523400
O	-0.16627100	2.14731900	-2.11854200
O	-0.16632100	2.14704300	2.11881500
N	1.55865800	-0.22458500	-0.00002800
C	2.71609600	-0.44092500	-0.00005000
C	4.15328000	-0.69494600	-0.00005500
H	4.45212000	-1.26861900	0.89626600
H	4.45208200	-1.26875000	-0.89630500
H	4.71071500	0.25936000	-0.00013700

$[Mn\text{-}H]^{-1}$

	x	y	z
C	-1.68348400	1.16339500	-0.18061800
C	-1.88462900	-0.00010100	-0.99312800
C	-1.68345200	-1.16344400	-0.18041400
C	-1.31614900	-0.71658200	1.14423700
C	-1.31616600	0.71677100	1.14410900
H	-1.74172200	2.19993400	-0.51792600
H	-2.12669600	-0.00019900	-2.05719500
H	-1.74165100	-2.20004500	-0.51754100
H	-1.08008500	-1.35911800	1.99494600
H	-1.08011300	1.35946200	1.99470400
Mn	0.15422500	0.00000200	-0.24148600
H	0.51690200	0.00000100	-1.75713900
C	1.34887800	1.25100700	-0.03884500
C	1.34886900	-1.25101300	-0.03884800
O	2.15717900	2.12363900	0.10989900
O	2.15713900	-2.12367500	0.10989500

$[Mn\text{-}CO]^0$

	x	y	z
C	-1.72663000	1.16193800	-0.39535600
C	-1.72464700	-0.00726200	-1.22544400
C	-1.72657400	-1.16631000	-0.38129100
C	-1.73589500	-0.70856600	0.98544200
C	-1.73582000	0.72072300	0.97683400
H	-1.72301500	2.19657900	-0.73981700
H	-1.70459300	-0.01385700	-2.31646800
H	-1.72301300	-2.20507200	-0.71310500
H	-1.72892900	-1.34259400	1.87324300
H	-1.72874000	1.36535100	1.85696500
Mn	0.06316900	0.00018400	0.00142900
C	1.04274200	1.28989200	0.73553200
C	1.04278500	-1.28020600	0.75173600
O	1.66162700	2.15671900	1.22758900
O	1.66005300	-2.14226100	1.25409800
C	1.05774100	-0.00906700	-1.47231100
O	1.68667800	-0.01594100	-2.46261200

L-L = bis CO
M = Fe

$[Fe\text{-}S]^+$

	x	y	z
C	1.27065000	-1.43230800	1.16582000
C	0.70112100	-2.03684800	-0.00046300
C	1.27128100	-1.43173500	-1.16611400
C	2.22744400	-0.44380400	-0.71613200
C	2.22708000	-0.44419600	0.71684500
H	1.03826800	-1.68061600	2.20236600
H	-0.08730400	-2.79155300	-0.00086900
H	1.03944500	-1.67952400	-2.20290600
H	2.84583100	0.19095900	-1.35369700
H	2.84512800	0.19024600	1.35505800
Fe	0.31078700	0.09403500	-0.00001000
C	0.16944200	1.30346500	1.29212200
C	0.16955400	1.30361800	-1.29202200
O	0.09915100	2.09187300	2.13901700
O	0.09933500	2.09213700	-2.13881700
N	-1.55430800	-0.26411100	-0.00007700
C	-2.70927700	-0.45617600	-0.00008800
C	-4.14541800	-0.69868800	-0.00006900
H	-4.43621900	-1.27047000	-0.89888600
H	-4.43619500	-1.27034200	0.89884400
H	-4.68839400	0.26311800	-0.00009300

$[Fe\text{-}H]^0$

	x	y	z
C	-1.61824200	1.16030800	-0.21747100
C	-1.80008500	-0.00019100	-1.03876500
C	-1.61818500	-1.16042400	-0.21711300
C	-1.34297300	-0.71260600	1.13195200
C	-1.34301000	0.71291000	1.13173100
H	-1.67320000	2.19707000	-0.55140000
H	-1.99802500	-0.00036200	-2.11083500
H	-1.67308400	-2.19729200	-0.55072500
H	-1.15057700	-1.35475800	1.99249000
H	-1.15063300	1.35533500	1.99206900
Fe	0.15363900	-0.00000300	-0.20317600
H	0.57953600	-0.00002100	-1.64393900
C	1.31973900	1.27458000	-0.01338900
C	1.31975300	-1.27457400	-0.01338200
O	2.09807600	2.14384300	0.09834700

O	2.09809800	-2.14383200	0.09834400

[Fe-CO]$^{+}$

	x	y	z
C	1.68690400	1.16511600	0.38960800
C	1.69938200	0.00165000	1.23089500
C	1.68407800	-1.16609300	0.39357100
C	1.70725500	-0.71833500	-0.98061300
C	1.70649700	0.71235400	-0.98383700
H	1.69150600	2.20330000	0.72653300
H	1.68972400	0.00386300	2.32312400
H	1.68814200	-2.20300100	0.73421600
H	1.71170700	-1.35954200	-1.86452100
H	1.71225300	1.35003600	-1.87022500
Fe	-0.06010200	0.00003900	-0.00203100
C	-1.02144900	1.32169900	-0.72635300
C	-1.02335800	-1.28723900	-0.78332800
O	-1.62654700	2.18543700	-1.19772300
O	-1.62997200	-2.12845100	-1.29199000
C	-1.03986200	-0.03253600	1.49172900
O	-1.65940000	-0.05390600	2.46642000

L-L = bis CO
M = Co

[Co-S]$^{2+}$

	x	y	z
C	1.32398600	-1.39283500	1.16800300
C	0.77915000	-2.02827800	-0.00042000
C	1.32465900	-1.39221000	-1.16815900
C	2.26441000	-0.38075100	-0.71832600
C	2.26403200	-0.38119100	0.71927900
H	1.10085400	-1.65032300	2.20715500
H	0.02432400	-2.81919100	-0.00085500
H	1.10211800	-1.64911900	-2.20758200
H	2.87242500	0.26735200	-1.35724700
H	2.87168900	0.26654800	1.35891200
Co	0.31848300	0.08889400	0.00004900
C	0.12149000	1.31850200	1.34668400
C	0.12177800	1.31839400	-1.34674300
O	-0.02934600	2.08945500	2.17806800
O	-0.02883300	2.08924700	-2.17826100
N	-1.54637800	-0.32390300	-0.00003800
C	-2.70121800	-0.52211000	-0.00012100
C	-4.12851800	-0.78003700	-0.00011900
H	-4.40865100	-1.35621000	-0.90149100
H	-4.40863200	-1.35635800	0.90116200
H	-4.68170000	0.17795500	-0.00003500

[Co-H]$^{+}$

	x	y	z
C	-1.58471500	1.15788700	-0.24451900
C	-1.75509500	-0.00017400	-1.07688300
C	-1.58466000	-1.15798200	-0.24416700
C	-1.39136800	-0.70905800	1.12913900
C	-1.39139200	0.70937400	1.12892400
H	-1.63479200	2.19696100	-0.57651100
H	-1.93722000	-0.00034000	-2.15269500
H	-1.63469300	-2.19715500	-0.57585500
H	-1.25018300	-1.35485600	1.99816700
H	-1.25024800	1.35543600	1.99776300
Co	0.15646500	0.00000300	-0.18425900
H	0.58605600	-0.00011000	-1.60775400
C	1.32139800	1.33275200	0.00948500
C	1.32138200	-1.33275100	0.00951300
O	2.08023000	2.19429100	0.10143300
O	2.08017300	-2.19433000	0.10143100

[Co-CO]$^{2+}$

	x	y	z
C	1.69057700	1.17007300	0.38608100
C	1.72423200	0.00195800	1.23240300
C	1.69063100	-1.16878000	0.38972600
C	1.72054800	-0.71973600	-0.99166900
C	1.72053000	0.71671500	-0.99390200
H	1.70322500	2.21133000	0.72452800
H	1.74064000	0.00366300	2.32793500
H	1.70332200	-2.20897700	0.73141600
H	1.74152700	-1.36244500	-1.87842100
H	1.74148400	1.35665400	-1.88265500
Co	-0.05444700	-0.00000200	-0.00270400
C	-1.03600700	1.35341200	-0.78323500
C	-1.03595800	-1.35613400	-0.77857300
O	-1.65412500	2.18603800	-1.26161100
O	-1.65393400	-2.19047900	-1.25412900
C	-1.03862800	0.00266800	1.55467100
O	-1.66389800	0.00428800	2.51038900

L-L = bpy
M = Mn

[Mn-S]^{0}

	x	y	z
C	-2.81499800	-1.17270200	-0.58109200
C	-3.16492400	0.00047500	0.16495300
C	-2.81465900	1.17297200	-0.58195000
C	-2.19891900	0.71901800	-1.80189700
C	-2.19911600	-0.71980800	-1.80135300
H	-2.99923700	-2.20607400	-0.28010100
H	-3.59253800	0.00091500	1.17060300
H	-2.99859400	2.20661500	-0.28170300
H	-1.78607800	1.35304300	-2.59057800
H	-1.78645900	-1.35452600	-2.58957500
Mn	-1.02686100	-0.00000800	-0.18701100
N	0.42266900	1.26162400	-0.38401200
C	0.32501800	2.62005000	-0.51423400
C	1.70321400	0.72547500	-0.27530000
C	1.42485900	3.47424200	-0.51375900
H	-0.69473700	3.00648100	-0.59272700
C	2.84952300	1.54955200	-0.22905300
C	2.72337500	2.93423800	-0.34947200
H	1.26630200	4.55279800	-0.61851100
H	3.83762700	1.09371700	-0.10331100
H	3.60511300	3.58282300	-0.31655100
N	0.42264600	-1.26165000	-0.38397200
C	1.70320000	-0.72552400	-0.27528200

C	0.32495400	-2.62008600	-0.51408200
C	2.84949100	-1.54962600	-0.22900900
C	1.42477300	-3.47430400	-0.51355600
H	-0.69481400	-3.00649900	-0.59250500
C	2.72330700	-2.93431600	-0.34934200
H	3.83760600	-1.09380900	-0.10330500
H	1.26618900	-4.55286600	-0.61821300
H	3.60503200	-3.58292000	-0.31640000
N	-0.76931500	-0.00002800	1.65055000
C	-0.58030800	0.00005600	2.82031200
C	-0.39564000	0.00027000	4.26805200
H	-0.85349500	0.89534900	4.73185900
H	-0.85172000	-0.89570000	4.73189500
H	0.68041500	0.00134000	4.52282000

$[Mn\text{-}H]^{-1}$

	x	y	z
C	-2.88603700	1.17401500	-0.20127300
C	-3.07236300	-0.00003100	-1.00710700
C	-2.88603100	-1.17403900	-0.20122200
C	-2.51208900	-0.72449100	1.11634100
C	-2.51209400	0.72452600	1.11631000
H	-2.98964600	2.20867300	-0.53920700
H	-3.30490500	-0.00005600	-2.07467700
H	-2.98963100	-2.20871300	-0.53911000
H	-2.26778000	-1.36662500	1.96864700
H	-2.26779000	1.36669800	1.96858800
Mn	-1.06458100	-0.00000200	-0.20294800
H	-0.85855600	-0.00000500	-1.76115800
N	0.34116100	-1.24331700	-0.03608100
C	0.24724300	-2.61770700	0.06125100
C	1.65674000	-0.71654600	-0.04944800
C	1.33617900	-3.47693400	0.10211600
H	-0.77735200	-3.00283300	0.07840600
C	2.79230700	-1.55850300	-0.04277100
C	2.65972900	-2.94629100	0.03246200
H	1.16042100	-4.55794100	0.17078900
H	3.78934600	-1.10225200	-0.09846800
H	3.53871400	-3.60164400	0.03633100
N	0.34115500	1.24331600	-0.03608000
C	1.65673700	0.71655200	-0.04944800
C	0.24723100	2.61770600	0.06125500
C	2.79230000	1.55851400	-0.04277300
C	1.33616300	3.47693800	0.10211700
H	-0.77736500	3.00282700	0.07841200
C	2.65971600	2.94630100	0.03246100
H	3.78934000	1.10226700	-0.09847100
H	1.16040100	4.55794400	0.17079000
H	3.53869700	3.60165800	0.03632700

$[Mn\text{-}CO]^{0}$

	x	y	z
C	2.79541600	1.17233700	-0.39589300
C	3.15832000	-0.00051300	0.35449000
C	2.79497500	-1.17281700	-0.39649800
C	2.17902500	-0.71658700	-1.61033500
C	2.17928400	0.71696200	-1.60994300
H	2.98845200	2.20604700	-0.10339500
H	3.64486900	-0.00086500	1.33204300
H	2.98767000	-2.20674600	-0.10455000
H	1.74475000	-1.34867600	-2.38844100
H	1.74522700	1.34962200	-2.38770900
Mn	1.04276400	-0.00006000	0.10073500
N	-0.42835600	-1.26564300	-0.09775900
C	-0.32079500	-2.62318100	-0.19040200
C	-1.70541400	-0.72763600	-0.08370100
C	-1.42102100	-3.47743500	-0.24407700
H	0.70170200	-3.00905400	-0.19089300
C	-2.85470100	-1.54569900	-0.09837800
C	-2.72265800	-2.93316600	-0.17987700
H	-1.25827600	-4.55794100	-0.31208900
H	-3.84758400	-1.08712100	-0.04781400
H	-3.60623400	-3.57970300	-0.19075800
N	-0.42823500	1.26565300	-0.09779900
C	-1.70534200	0.72777600	-0.08371900
C	-0.32051900	2.62317700	-0.19043500
C	-2.85454100	1.54596500	-0.09840100
C	-1.42065200	3.47755000	-0.24410900
H	0.70202400	3.00893100	-0.19091600
C	-2.72234700	2.93341700	-0.17991100
H	-3.84747500	1.08749700	-0.04782600
H	-1.25779400	4.55804000	-0.31211600
H	-3.60585300	3.58004800	-0.19079700
C	0.91886200	0.00003700	1.85832700
O	0.87102400	0.00002900	3.04287100

L-L = bpy
M = Fe

$[Fe\text{-}S]^{1+}$

	x	y	z
C	2.56265800	1.16824900	-1.06947400
C	3.09159900	-0.00082000	-0.43058500
C	2.56190600	-1.16919400	-1.07004800
C	1.69409200	-0.71490800	-2.12913300
C	1.69459600	0.71502600	-2.12878600
H	2.80856800	2.20129400	-0.81922600
H	3.74702200	-0.00125200	0.44253000
H	2.80713000	-2.20251800	-0.82028000
H	1.10669100	-1.34794800	-2.79706700
H	1.10757900	1.34877600	-2.79638700
Fe	0.97524000	-0.00013700	-0.28399400
N	-0.49241400	-1.27639500	-0.21038600
C	-0.36986500	-2.62905100	-0.20214200
C	-1.75233400	-0.73360200	-0.13661900
C	-1.47257900	-3.48752100	-0.14791600
H	0.65152200	-3.01458700	-0.23700200
C	-2.90020900	-1.54469800	-0.06802100
C	-2.76447300	-2.93741800	-0.08044200
H	-1.31268100	-4.56989700	-0.15302900
H	-3.89235500	-1.08820500	-0.00701700
H	-3.64797900	-3.58176100	-0.03335600
N	-0.49211800	1.27642500	-0.21055300
C	-1.75216100	0.73394500	-0.13669300
C	-0.36921900	2.62905000	-0.20242200
C	-2.89983200	1.54533600	-0.06812700
C	-1.47171400	3.48780000	-0.14822500
H	0.65227200	3.01431400	-0.23733500
C	-2.76374400	2.93802100	-0.08066700
H	-3.89209000	1.08910100	-0.00704300
H	-1.31154900	4.57013600	-0.15342000
H	-3.64708700	3.58258900	-0.03359800

	x	y	z
N	1.11853700	-0.00000100	1.58287000
C	1.22757300	0.00007700	2.75305200
C	1.38956600	0.00016700	4.20314200
H	1.94580300	-0.89622900	4.53079100
H	1.94460000	0.89729200	4.53082900
H	0.40311800	-0.00051400	4.69956500

[Fe-H]^{0}

	x	y	z
C	-2.82295600	1.16563000	-0.17069400
C	-3.02092500	-0.00038100	-0.98353300
C	-2.82284400	-1.16597000	-0.17014400
C	-2.48121500	-0.71738700	1.15619700
C	-2.48127900	0.71769100	1.15585100
H	-2.91571000	2.19943200	-0.50881200
H	-3.26447700	-0.00064800	-2.04682700
H	-2.91549000	-2.19994200	-0.50777200
H	-2.24068700	-1.35580200	2.00922600
H	-2.24079100	1.35652400	2.00858100
Fe	-1.04809800	-0.00004600	-0.21126200
H	-0.79158600	-0.00002200	-1.70659600
N	0.34837700	-1.24994200	-0.05684300
C	0.23242200	-2.61462000	-0.01675700
C	1.63549200	-0.72414500	-0.02851100
C	1.32454500	-3.47423500	0.04739000
H	-0.78983700	-2.99750600	-0.05102200
C	2.77609400	-1.55240900	0.01375100
C	2.63424800	-2.93979400	0.05453700
H	1.15174900	-4.55504900	0.07956000
H	3.77198200	-1.09708500	0.01669500
H	3.51176500	-3.59346100	0.08962600
N	0.34827300	1.24994500	-0.05681900
C	1.63543200	0.72425700	-0.02851000
C	0.23219500	2.61461200	-0.01671600
C	2.77596200	1.55262100	0.01372200
C	1.32424600	3.47432100	0.04740200
H	-0.79010000	2.99740900	-0.05094500
C	2.63399600	2.93999300	0.05450700
H	3.77189100	1.09738400	0.01663900
H	1.15135800	4.55512000	0.07957900
H	3.51145700	3.59373700	0.08956700

[Fe-CO]$^{1+}$

	x	y	z
C	2.66805100	1.16576300	-0.60346900
C	3.14577000	-0.00189100	0.08601500
C	2.66632700	-1.16772400	-0.60521500
C	1.92302200	-0.71157700	-1.75217000
C	1.92415900	0.71241500	-1.75110200
H	2.88777900	2.19978300	-0.33388500
H	3.75695700	-0.00303400	0.99072400
H	2.88448600	-2.20247200	-0.33715600
H	1.40584200	-1.34589100	-2.47461000
H	1.40785900	1.34860000	-2.47253200
Fe	1.03894300	-0.00031900	0.09489800
N	-0.43842900	-1.28006300	0.02162100
C	-0.31920700	-2.62888700	0.10050000
C	-1.68843400	-0.73397900	-0.12497100
C	-1.41978400	-3.48708100	0.00378000
H	0.69187400	-3.01224700	0.25314600
C	-2.83368400	-1.54466600	-0.22554900
C	-2.70069100	-2.93685600	-0.16852200
H	-1.26613100	-4.56810400	0.07075300
H	-3.82111300	-1.08952100	-0.34261300
H	-3.58217300	-3.58099000	-0.24608600
N	-0.43769600	1.28020400	0.02157600
C	-1.68801400	0.73484200	-0.12496800
C	-0.31765300	2.62895900	0.10040800
C	-2.83279300	1.54620600	-0.22547800
C	-1.41773100	3.48779700	0.00376700
H	0.69368800	3.01169300	0.25293600
C	-2.69897400	2.93831500	-0.16843900
H	-3.82049800	1.09164700	-0.34248400
H	-1.26344500	4.56873000	0.07072400
H	-3.58008400	3.58296700	-0.24592800
C	1.11323800	-0.00028600	1.84285000
O	1.19796500	-0.00024400	3.00783100

L-L = bpy
M = Co

[Co-S]$^{2+}$

	x	y	z
C	2.45139400	1.16353600	-1.24511900
C	3.05628100	-0.00334400	-0.67182600
C	2.44787400	-1.16649700	-1.24853200
C	1.48613000	-0.71423100	-2.22619000
C	1.48860500	0.71709000	-2.22427400
H	2.71089200	2.19681900	-1.00868200
H	3.80311700	-0.00567700	0.12497800
H	2.70411700	-2.20127300	-1.01511100
H	0.84360200	-1.34787100	-2.84110000
H	0.84803100	1.35449700	-2.83733300
Co	0.94186900	-0.00043700	-0.31631800
N	-0.51222600	-1.29480100	-0.14650400
C	-0.37268300	-2.64005200	-0.06981700
C	-1.76242300	-0.73506200	-0.10754500
C	-1.47903800	-3.49493700	0.01616000
H	0.64707000	-3.03102700	-0.07355100
C	-2.90886400	-1.54515600	-0.02737600
C	-2.76813200	-2.93878200	0.02543100
H	-1.32023400	-4.57611600	0.07056100
H	-3.90365700	-1.09188100	-0.00091100
H	-3.65326800	-3.58047300	0.08283100
N	-0.51107400	1.29505800	-0.14636700
C	-1.76176300	0.73643600	-0.10748000
C	-0.37026000	2.64017700	-0.06966000
C	-2.90745400	1.54759200	-0.02727300
C	-1.47582600	3.49606900	0.01636100
H	0.64987400	3.03017400	-0.07348800
C	-2.76543000	2.94108200	0.02561200
H	-3.90266500	1.09523700	-0.00085500
H	-1.31604100	4.57710300	0.07077600
H	-3.64997800	3.58358300	0.08303600
N	1.24896500	-0.00067900	1.56228000
C	1.45284900	-0.00074400	2.71487600
C	1.71718600	-0.00081400	4.14592200
H	2.29522400	-0.89988400	4.42524600
H	2.29700800	0.89718000	4.42499800
H	0.76611800	0.00018900	4.70770700

[Co-H]$^{1+}$

	x	y	z
C	-2.78821300	1.15624800	-0.10421500
C	-3.02951800	-0.00107300	-0.91786700
C	-2.78768700	-1.15736100	-0.10293600
C	-2.46936900	-0.70833300	1.23770400
C	-2.46968800	0.70878500	1.23690700
H	-2.88696200	2.19134000	-0.43534800
H	-3.31479100	-0.00172300	-1.97024400
H	-2.88591800	-2.19286100	-0.43294700
H	-2.23180100	-1.34948600	2.08857500
H	-2.23233600	1.35096700	2.08706400
Co	-1.04697600	-0.00016100	-0.24420700
N	0.36849700	-1.27890800	-0.10562600
C	0.24704900	-2.63005400	-0.15432400
C	1.62223500	-0.73276700	0.02203400
C	1.34718900	-3.48685900	-0.05322000
H	-0.76400700	-3.01973500	-0.28195400
C	2.76666900	-1.54425000	0.12234400
C	2.63189800	-2.93640900	0.08947700
H	1.18973200	-4.56864000	-0.09309700
H	3.75541800	-1.08802700	0.22208000
H	3.51319900	-3.58060400	0.16772000
N	0.36810800	1.27895300	-0.10558800
C	1.62201200	0.73320300	0.02204200
C	0.24620800	2.63005700	-0.15429000
C	2.76618600	1.54505700	0.12232000
C	1.34607300	3.48721600	-0.05322500
H	-0.76498800	3.01938700	-0.28190000
C	2.63096200	2.93717300	0.08944200
H	3.75508700	1.08915900	0.22202800
H	1.18827400	4.56894700	-0.09311300
H	3.51205900	3.58165100	0.16764900
H	-0.81287900	-0.00012500	-1.70758600

[Co-CO]$^{2+}$

	x	y	z
C	2.65732200	1.16309400	-0.61122400
C	3.16144100	-0.00305900	0.06448400
C	2.65468800	-1.16623300	-0.61412000
C	1.89545900	-0.71277500	-1.75610100
C	1.89729300	0.71419100	-1.75440700
H	2.87247500	2.19788700	-0.33702200
H	3.79378900	-0.00491200	0.95658400
H	2.86732700	-2.20220100	-0.34242700
H	1.38121200	-1.34952600	-2.47948900
H	1.38449500	1.35392900	-2.47619000
Co	1.02699000	-0.00047800	0.08759600
N	-0.43784300	-1.29761100	0.02975300
C	-0.31287700	-2.64282400	0.14617900
C	-1.67483300	-0.73449700	-0.15654900
C	-1.41865700	-3.49570000	0.04178700
H	0.68900300	-3.03502500	0.33295200
C	-2.81629000	-1.54660500	-0.27491400
C	-2.68805200	-2.93977300	-0.18375700
H	-1.27308600	-4.57578000	0.13997800
H	-3.79997400	-1.09401800	-0.42771100
H	-3.57070100	-3.58124300	-0.27410500
N	-0.43668300	1.29784200	0.02964400
C	-1.67417900	0.73582500	-0.15655300
C	-0.31045400	2.64294600	0.14599200
C	-2.81491100	1.54897500	-0.27479900
C	-1.41547200	3.49681600	0.04172900
H	0.69183300	3.03421300	0.33256000
C	-2.68540100	2.94202200	-0.18363900
H	-3.79902300	1.09728400	-0.42748600
H	-1.26891600	4.57676700	0.13987500
H	-3.56748400	3.58428900	-0.27386000
C	1.08198700	-0.00038600	1.87944100
O	1.09620300	-0.00031000	3.02927000

L-L = dppe
M = Mn

[Mn-S]^{0}

	x	y	z
C	-1.33595200	0.23622500	2.78729600
C	-0.52992600	-0.87382000	3.18799900
C	0.85442900	-0.47254300	3.15378200
C	0.89029200	0.89630500	2.72020000
C	-0.45715100	1.34409000	2.49985800
H	-2.42563000	0.25403000	2.72813400
H	-0.89771700	-1.87169000	3.43728900
H	1.71464000	-1.09810900	3.39840600
H	1.79061000	1.50341000	2.60201500
H	-0.77515400	2.34508500	2.20390300
Mn	0.00290600	-0.30031000	1.19054200
C	0.74373200	-0.17946000	-2.04270600
H	1.37817800	0.19509800	-2.86777500
H	0.64865200	-1.27689900	-2.14431000
C	-0.64856100	0.46662000	-2.05316500
H	-1.24427800	0.12860900	-2.91943100
H	-0.55834000	1.56574900	-2.12479600
P	1.51601800	0.15583200	-0.34640500
P	-1.49360600	0.06471700	-0.39417500
C	3.07480500	-0.87425100	-0.38712100
C	3.38387900	-1.80076600	-1.40640800
C	3.96080300	-0.76944800	0.71070100
C	4.54349500	-2.59723300	-1.33235400
H	2.72822700	-1.90540200	-2.27761800
C	5.12254900	-1.55234200	0.78164000
H	3.73535200	-0.06339600	1.51927100
C	5.41723800	-2.47559300	-0.24026000
H	4.76421100	-3.30883700	-2.13768800
H	5.79897900	-1.44581300	1.63832400
H	6.32218200	-3.09193200	-0.18410000
C	2.25585700	1.85528700	-0.62353900
C	1.68393100	2.97955300	0.00696500
C	3.35522000	2.05363200	-1.48978800
C	2.19199200	4.27172100	-0.22089500
H	0.83795500	2.83018300	0.68624300
C	3.86081600	3.34315900	-1.72447300
H	3.82847400	1.19153500	-1.97550900
C	3.28038500	4.45687200	-1.08997400
H	1.73862200	5.13252300	0.28549300
H	4.71473100	3.47820300	-2.39939400
H	3.67930900	5.46238000	-1.26871600
C	-2.69789100	1.48485000	-0.21401000
C	-2.24690300	2.81729400	-0.35546000

C	-4.04269900	1.27327700	0.16370300
C	-3.11457300	3.90215200	-0.14507700
H	-1.20396400	3.01643200	-0.62871100
C	-4.91001100	2.35834000	0.38544300
H	-4.42288900	0.25181100	0.27793800
C	-4.45135700	3.67697900	0.22910200
H	-2.74278900	4.92622400	-0.27172700
H	-5.94971600	2.16807900	0.67826600
H	-5.12852300	4.52257600	0.39721000
C	-2.68814900	-1.30720100	-0.86822500
C	-3.39039700	-1.34584700	-2.09387300
C	-2.92624200	-2.33012100	0.07405000
C	-4.29193900	-2.38644800	-2.37630600
H	-3.25543700	-0.54714600	-2.83322200
C	-3.83528800	-3.36814100	-0.20037800
H	-2.38568800	-2.29918600	1.02693800
C	-4.51572300	-3.40287300	-1.42951400
H	-4.82861900	-2.39747900	-3.33286700
H	-4.01075300	-4.15139900	0.54739400
H	-5.22135900	-4.21324200	-1.64823200
N	0.21714300	-2.08973800	0.74361700
C	0.37775500	-3.24568500	0.52356400
C	0.63874000	-4.65793900	0.26168800
H	1.72067100	-4.84734300	0.11884300
H	0.29276100	-5.28882200	1.10273800
H	0.10953100	-4.99743800	-0.64927300

[Mn-H]$^{-1}$

	x	y	z
C	-1.20556200	-0.21090800	2.95826800
C	-0.40942100	-1.40051100	3.06670200
C	0.98227000	-1.01708300	3.02824600
C	1.02756100	0.41947000	2.87638600
C	-0.31990300	0.92167900	2.85025700
H	-2.29741600	-0.16975700	2.94554800
H	-0.79137100	-2.41931600	3.16106500
H	1.84110400	-1.68860700	3.08027100
H	1.93766800	1.02342100	2.82957700
H	-0.62577300	1.96694700	2.76121900
Mn	0.03299500	-0.45132200	1.22394300
H	0.10345300	-1.91072500	0.63282700
C	0.74100100	-0.46133100	-2.02311200
H	1.38150300	-0.06806400	-2.83546800
H	0.66500200	-1.55718300	-2.14174500
C	-0.65283600	0.17910800	-2.02898500
H	-1.29105900	-0.22536800	-2.83750200
H	-0.56714700	1.27099000	-2.18049100
P	1.49415100	-0.13036500	-0.29972400
P	-1.43701400	-0.11804400	-0.30076900
C	3.02287900	-1.22741500	-0.34569500
C	2.88522200	-2.60277300	-0.65386200
C	4.29148400	-0.78109200	0.09639200
C	3.97318800	-3.48517300	-0.56378200
H	1.89951900	-2.99127300	-0.93683000
C	5.38044100	-1.66534800	0.20729100
H	4.43171500	0.27475300	0.35802500
C	5.23207200	-3.02188300	-0.13049600
H	3.83600700	-4.54409600	-0.82049400
H	6.35096700	-1.28856800	0.55763100
H	6.08133200	-3.71230000	-0.04915200
C	2.32074000	1.54072700	-0.66562400
C	1.96558700	2.64588900	0.13495600
C	3.20038300	1.76072900	-1.75145700
C	2.47372400	3.93149400	-0.12985700
H	1.26629900	2.47446600	0.96385300
C	3.69555600	3.04687100	-2.03639100
H	3.51340500	0.91323100	-2.37532900
C	3.33472700	4.13810500	-1.22434600
H	2.19082800	4.77471700	0.51410600
H	4.36777800	3.19731400	-2.89211200
H	3.72135800	5.14175200	-1.44420300
C	-2.62602900	1.33882700	-0.17201000
C	-2.14185700	2.65483700	-0.36557200
C	-3.94983200	1.19312500	0.30694300
C	-2.95591900	3.77612000	-0.13404600
H	-1.10209900	2.80556000	-0.68209000
C	-4.76251000	2.31399100	0.56289000
H	-4.35451100	0.18834300	0.47750700
C	-4.27479400	3.61204900	0.33505900
H	-2.55539700	4.78328000	-0.30975300
H	-5.78392700	2.16833000	0.93962800
H	-4.90902400	4.48676300	0.52801100
C	-2.69788800	-1.46213200	-0.78967700
C	-3.62733000	-1.32503100	-1.84577200
C	-2.66717400	-2.68050300	-0.08265400
C	-4.49813500	-2.37558000	-2.18722900
H	-3.68334200	-0.37949900	-2.40196100
C	-3.53834800	-3.73487800	-0.41486300
H	-1.92196500	-2.77837500	0.71920200
C	-4.45627300	-3.58692600	-1.47033200
H	-5.21262900	-2.24942000	-3.01216900
H	-3.49742600	-4.67650400	0.14910500
H	-5.13494800	-4.40833500	-1.73523800

[Mn-CO]^{0}

	x	y	z
C	-0.80746100	-1.11386100	3.06998300
C	0.63338700	-1.16792400	3.10814300
C	1.13032000	0.13362000	2.78154200
C	0.00730600	1.00333300	2.54657500
C	-1.18701900	0.22105900	2.71933600
H	-1.48502800	-1.94483800	3.27290000
H	1.23548800	-2.04687500	3.34480100
H	2.18114200	0.42288900	2.74786500
H	0.05133800	2.06331800	2.29510600
H	-2.20889300	0.58847400	2.61116600
Mn	-0.01270100	-0.63068800	1.12328700
C	0.74110900	-0.37098900	-2.09090800
H	1.36276300	0.07476200	-2.88947500
H	0.74013500	-1.46496200	-2.24727600
C	-0.69645800	0.17461900	-2.10994400
H	-1.29497100	-0.32767900	-2.88986200
H	-0.71535000	1.25668400	-2.33083000
P	1.51935000	-0.14090500	-0.38519600
P	-1.51222600	-0.06305500	-0.39734600
C	3.06898400	-1.17265900	-0.54781400
C	3.29978200	-2.23271100	0.35066700
C	4.02302300	-0.91598900	-1.55927700
C	4.45463400	-3.02801800	0.24049900
H	2.56051300	-2.43439000	1.13281800
C	5.17253800	-1.71391200	-1.67581400
H	3.87548700	-0.08009900	-2.25421200

C	5.39149600	-2.77238400	-0.77417200
H	4.61772000	-3.85076500	0.94666000
H	5.90215400	-1.50557600	-2.46756600
H	6.29097400	-3.39290100	-0.86368800
C	2.29560300	1.55791200	-0.47068300
C	3.31216200	1.89684600	0.45296600
C	1.89308700	2.53950500	-1.40323000
C	3.89245300	3.17519900	0.45638000
H	3.67756300	1.14174600	1.15880700
C	2.47578800	3.82040600	-1.40573700
H	1.12851600	2.30927600	-2.15308900
C	3.47251000	4.14568700	-0.47125400
H	4.68242500	3.41040000	1.17953100
H	2.15120700	4.56124900	-2.14638500
H	3.92699400	5.14312300	-0.47202900
C	-2.41716600	1.55809400	-0.16599300
C	-1.67467000	2.76141600	-0.17808000
C	-3.79977500	1.62672100	0.11354500
C	-2.29924100	3.99768600	0.05368400
H	-0.59348700	2.73552500	-0.35955600
C	-4.42392500	2.86416600	0.35775900
H	-4.40024100	0.71093300	0.13563700
C	-3.67868900	4.05407200	0.32354200
H	-1.70350700	4.91815200	0.03146300
H	-5.49892600	2.89330400	0.57257400
H	-4.16636400	5.01806400	0.50962600
C	-2.94155300	-1.20092700	-0.78579100
C	-3.73118100	-1.04608100	-1.94879500
C	-3.26988500	-2.22981200	0.11977000
C	-4.81028700	-1.90802200	-2.20406200
H	-3.51932900	-0.23576800	-2.65706300
C	-4.35425400	-3.09001300	-0.12963100
H	-2.65738000	-2.36003700	1.01828200
C	-5.12383600	-2.93451600	-1.29417900
H	-5.40937100	-1.77539600	-3.11296200
H	-4.58901200	-3.88897700	0.58344000
H	-5.96456200	-3.60898200	-1.49477700
C	-0.01584400	-2.29181600	0.57197000
O	-0.02062100	-3.44353500	0.26850900

L-L = dppe
M = Fe

$[Fe\text{-}S]^+$

	x	y	z
C	-1.31420100	0.24641500	2.73911800
C	-0.47743800	-0.83068000	3.15481600
C	0.89775800	-0.40100500	3.08241600
C	0.89740100	0.95744600	2.62426400
C	-0.46165200	1.36163800	2.39544300
H	-2.40418100	0.23862600	2.69344800
H	-0.81698800	-1.82914700	3.43683100
H	1.77381300	-1.00218400	3.32871500
H	1.78020900	1.57997600	2.46707900
H	-0.80672400	2.34521900	2.07509000
Fe	0.00911200	-0.27684600	1.15824900
C	0.74136100	-0.17496100	-2.03384000
H	1.37118800	0.17873800	-2.86993000
H	0.62053800	-1.26955800	-2.12712300
C	-0.63217600	0.51186400	-2.03306500
H	-1.22794100	0.22246000	-2.91544000
H	-0.51146500	1.60912800	-2.07154300
P	1.55819600	0.17471300	-0.37917200
P	-1.52360000	0.09107700	-0.42765900
C	3.07358500	-0.88449000	-0.34898800
C	3.37658400	-1.81966500	-1.36205400
C	3.95407600	-0.76135800	0.75077700
C	4.53566900	-2.61381400	-1.27665800
H	2.72671500	-1.92737200	-2.23697100
C	5.11075800	-1.55104000	0.83274900
H	3.74397400	-0.03194300	1.54202300
C	5.40310600	-2.48338000	-0.18008300
H	4.76346200	-3.32656100	-2.07759400
H	5.78744800	-1.43635700	1.68696000
H	6.30770900	-3.09810300	-0.11713700
C	2.25674000	1.87589700	-0.61296400
C	1.59265300	3.00700400	-0.09257500
C	3.43474700	2.06408400	-1.37305500
C	2.09091500	4.30094700	-0.32629500
H	0.68682300	2.87483100	0.50707600
C	3.92868100	3.35718500	-1.60857100
H	3.97574300	1.19961300	-1.77457000
C	3.25898000	4.47816400	-1.08593400
H	1.56873800	5.16888200	0.09212400
H	4.84351600	3.48791300	-2.19732000
H	3.65100000	5.48528900	-1.26527200
C	-2.73952500	1.47447200	-0.20591300
C	-2.34068900	2.81928900	-0.38334000
C	-4.06678000	1.20705800	0.19861900
C	-3.25100100	3.86874200	-0.17559700
H	-1.31751500	3.06148800	-0.69277200
C	-4.97252900	2.26000300	0.41487500
H	-4.40508500	0.17347700	0.33049700
C	-4.56918800	3.59244300	0.22731200
H	-2.92846000	4.90431700	-0.33225200
H	-5.99934100	2.03350900	0.72325800
H	-5.27846700	4.41143800	0.38899800
C	-2.63022200	-1.33947900	-0.84877600
C	-3.27136700	-1.44975700	-2.10343300
C	-2.90782500	-2.30403000	0.14441100
C	-4.15220800	-2.51272100	-2.36391700
H	-3.10764500	-0.69770600	-2.88340500
C	-3.79927500	-3.36050100	-0.11216800
H	-2.42318900	-2.22087200	1.12320000
C	-4.41762900	-3.47079500	-1.36942500
H	-4.64007100	-2.58470500	-3.34243300
H	-4.01309200	-4.09564200	0.67231300
H	-5.11021100	-4.29523600	-1.57211300
N	0.21831400	-2.06617000	0.69996500
C	0.38829500	-3.20917600	0.47575600
C	0.59107300	-4.62801000	0.20453200
H	1.59549200	-4.80077900	-0.22170500
H	0.50472000	-5.21387100	1.13715100
H	-0.16684800	-4.99726700	-0.50954300

$[Fe\text{-}H]^0$

	x	y	z
C	-1.18372400	-0.26996100	2.87023200
C	-0.37810000	-1.45396800	2.95906200
C	1.00642400	-1.05838200	2.91939400
C	1.04066500	0.37960900	2.80834900
C	-0.30622200	0.87328300	2.78656100

H	-2.27506200	-0.23664700	2.86930600
H	-0.74941600	-2.47727700	3.03378200
H	1.87106700	-1.72224600	2.94898600
H	1.94666100	0.98788900	2.76119100
H	-0.61971600	1.91716900	2.72862600
Fe	0.03953800	-0.46279200	1.16559500
H	0.11210400	-1.83609200	0.51045000
C	0.72077500	-0.45663300	-2.01760100
H	1.35414400	-0.09946600	-2.84978500
H	0.60437800	-1.55025800	-2.11673500
C	-0.64933400	0.23751800	-2.00340500
H	-1.29177500	-0.09867700	-2.83697200
H	-0.51831200	1.33061800	-2.09771900
P	1.51793500	-0.11636600	-0.34199200
P	-1.46674800	-0.09993900	-0.32464800
C	3.02781000	-1.20583500	-0.32515700
C	2.89229500	-2.58153000	-0.62085900
C	4.28942700	-0.73004700	0.09562600
C	3.99262300	-3.44886600	-0.53149700
H	1.91309900	-2.98466400	-0.90456100
C	5.38872000	-1.60130300	0.19975700
H	4.42092100	0.32965500	0.34213400
C	5.24660400	-2.96197500	-0.11905000
H	3.86765100	-4.51058400	-0.77581300
H	6.35873600	-1.21117200	0.53082500
H	6.10438200	-3.64031000	-0.04232000
C	2.26785600	1.56995800	-0.65968300
C	1.87997800	2.66048800	0.14489700
C	3.16731200	1.80256600	-1.72601600
C	2.37957800	3.95284700	-0.10130000
H	1.17473200	2.48434200	0.96525600
C	3.66105900	3.09287300	-1.98025200
H	3.49706400	0.96754400	-2.35653700
C	3.26876900	4.17229400	-1.16675100
H	2.07060500	4.78794500	0.53896800
H	4.35602400	3.25574700	-2.81286200
H	3.65637000	5.17870800	-1.36404900
C	-2.63597700	1.34063100	-0.12507300
C	-2.15023600	2.65971900	-0.27805100
C	-3.97185300	1.16428300	0.29941500
C	-2.98342700	3.76689200	-0.04923900
H	-1.10731700	2.82940700	-0.57164000
C	-4.80283000	2.27270000	0.54306400
H	-4.37371400	0.15373300	0.43369000
C	-4.31475000	3.57765500	0.36407700
H	-2.58866700	4.78054100	-0.18845400
H	-5.83683400	2.11135100	0.87101100
H	-4.96448400	4.44135800	0.54745700
C	-2.65730300	-1.47497700	-0.77328700
C	-3.58117800	-1.35116900	-1.83652600
C	-2.61181200	-2.68526900	-0.05379200
C	-4.43288200	-2.41624000	-2.17332700
H	-3.64782200	-0.41242500	-2.40085000
C	-3.46718400	-3.75188200	-0.38536700
H	-1.88049900	-2.78040800	0.75728000
C	-4.37774000	-3.62086300	-1.44721000
H	-5.14193000	-2.30558700	-3.00270800
H	-3.41779400	-4.68741300	0.18489600
H	-5.04244200	-4.45212700	-1.71068700

$[Fe\text{-}CO]^+$

	x	y	z
C	-0.85522500	-0.85172800	3.02752300
C	0.56755500	-1.08991600	3.07604500
C	1.23137300	0.11270000	2.67950600
C	0.22715800	1.10342900	2.37620700
C	-1.05526300	0.50177800	2.60701900
H	-1.63539700	-1.56878300	3.28619100
H	1.04901800	-2.02555900	3.36547200
H	2.31101600	0.25658300	2.63553700
H	0.41222700	2.12723500	2.05154600
H	-2.02009000	0.99047400	2.46529200
Fe	0.00301200	-0.57407900	1.08753100
C	0.71733000	-0.37702500	-2.10283500
H	1.32698000	0.03991500	-2.92458300
H	0.69347100	-1.47344300	-2.23903200
C	-0.70746100	0.20096600	-2.10791000
H	-1.31268000	-0.27417100	-2.89867700
H	-0.70816400	1.28557500	-2.31058800
P	1.55489600	-0.11634700	-0.44766200
P	-1.55666100	-0.03954900	-0.43486000
C	3.05064900	-1.19667200	-0.51665500
C	3.28523200	-2.18026600	0.46510300
C	3.98241300	-1.03198900	-1.56865600
C	4.42582600	-2.99883700	0.39284900
H	2.57437400	-2.30961700	1.28727600
C	5.11504000	-1.85656000	-1.64305500
H	3.83308700	-0.25300300	-2.32571800
C	5.33851800	-2.84111700	-0.66254400
H	4.59759900	-3.76066400	1.16116500
H	5.82762000	-1.72597200	-2.46491000
H	6.22573300	-3.48094000	-0.72158300
C	2.30174900	1.57700500	-0.50569900
C	3.36416800	1.88537100	0.37615800
C	1.85551000	2.57257500	-1.40307400
C	3.94894000	3.16128100	0.37360800
H	3.76827800	1.11409100	1.04209800
C	2.44810900	3.84798900	-1.40979800
H	1.06177100	2.36034400	-2.12634200
C	3.48849100	4.14873900	-0.51563900
H	4.77655900	3.37875700	1.05780100
H	2.09914800	4.60231600	-2.12392100
H	3.94997400	5.14209400	-0.52193000
C	-2.47490700	1.54824900	-0.17069500
C	-1.76573700	2.77207400	-0.17854200
C	-3.86148700	1.56074600	0.09744000
C	-2.43545200	3.98381600	0.05341100
H	-0.68434500	2.78769500	-0.36058900
C	-4.52652900	2.77705500	0.33566300
H	-4.43050700	0.62550800	0.11254800
C	-3.81840500	3.98946100	0.31146100
H	-1.87466700	4.92509800	0.03573600
H	-5.60354200	2.77155500	0.53670600
H	-4.33950100	4.93556600	0.49375800
C	-2.90447800	-1.26424000	-0.74935300
C	-3.67330700	-1.20179800	-1.93571700
C	-3.22453500	-2.23531900	0.22195200
C	-4.72243200	-2.10949100	-2.14968300
H	-3.47400300	-0.43493300	-2.69326300
C	-4.27968700	-3.13879100	0.00910600
H	-2.64187100	-2.29034000	1.14661300
C	-5.02554800	-3.08172800	-1.17937000
H	-5.30609700	-2.05335600	-3.07514600

H	-4.51167700	-3.89172900	0.77021400
H	-5.84311600	-3.79042300	-1.35049200
C	-0.01578800	-2.23970400	0.59005900
O	-0.02299600	-3.37921000	0.31094200

L-L = dppe
M = Co

[Co-S]$^{2+}$

	x	y	z
C	-1.28380200	0.47351000	2.69349000
C	-0.49243200	-0.60276700	3.18145200
C	0.90337000	-0.23500900	3.07526100
C	0.96506300	1.08445800	2.53260300
C	-0.38200500	1.51584000	2.25209400
H	-2.37327300	0.51322100	2.65011900
H	-0.86932900	-1.56140800	3.54339100
H	1.75218300	-0.85791700	3.36131800
H	1.86838800	1.66621800	2.34180600
H	-0.68465800	2.49136600	1.87045600
Co	0.01463800	-0.19060800	1.12358300
C	0.71330000	-0.13778800	-2.05337200
H	1.33399100	0.19697000	-2.90384600
H	0.56198300	-1.22846300	-2.14037100
C	-0.63587300	0.59433100	-2.02846600
H	-1.23499000	0.36815200	-2.92708500
H	-0.48691000	1.68815100	-2.01854800
P	1.60822400	0.20581300	-0.44950100
P	-1.57969300	0.14097200	-0.48120800
C	3.03543700	-0.93450700	-0.34722700
C	3.28976000	-1.90647400	-1.34189500
C	3.91949700	-0.81804500	0.75259300
C	4.41564500	-2.74261900	-1.23749300
H	2.64055700	-2.00202100	-2.21845500
C	5.03752900	-1.65896300	0.85329000
H	3.75229700	-0.05372200	1.52057100
C	5.28623300	-2.62448400	-0.14050700
H	4.61870800	-3.47637700	-2.02520300
H	5.72164200	-1.55413100	1.70218600
H	6.16478500	-3.27396300	-0.06499800
C	2.32278100	1.88911400	-0.62102400
C	1.55329400	3.03880400	-0.33069000
C	3.63296600	2.04332800	-1.13579200
C	2.07919300	4.32121300	-0.55346900
H	0.54466900	2.93420000	0.08116100
C	4.15225000	3.32975500	-1.35607900
H	4.24935200	1.16806100	-1.36405500
C	3.38043900	4.46804200	-1.06618700
H	1.47715800	5.20598800	-0.31983100
H	5.16730800	3.43893600	-1.75259500
H	3.79362600	5.46826400	-1.23343600
C	-2.83860800	1.45732900	-0.23446000
C	-2.51733100	2.82050400	-0.44439500
C	-4.14609700	1.11153800	0.18322000
C	-3.49102100	3.81368700	-0.25478100
H	-1.51893400	3.12233200	-0.78031300
C	-5.11171000	2.11259600	0.37836300
H	-4.42431900	0.06266400	0.32946300
C	-4.78811900	3.46286400	0.16122100
H	-3.23756200	4.86292700	-0.44068300
H	-6.12375000	1.83168400	0.68884400
H	-5.54597100	4.23982200	0.30644100
C	-2.54019400	-1.38806100	-0.83358300
C	-2.98548100	-1.68874200	-2.14162400
C	-2.94261000	-2.21682500	0.24001000
C	-3.80631400	-2.80658400	-2.36880900
H	-2.72151400	-1.05275900	-2.99274700
C	-3.77409500	-3.32485300	0.00886600
H	-2.61248300	-1.99414200	1.26102100
C	-4.20291000	-3.62445900	-1.29692100
H	-4.14544800	-3.02781200	-3.38644900
H	-4.09327200	-3.95048900	0.84979800
H	-4.85166200	-4.48824300	-1.47717300
N	0.19245100	-2.02076300	0.74164300
C	0.33388200	-3.17277500	0.58316800
C	0.49846300	-4.60544700	0.38519700
H	1.49107300	-4.81740200	-0.04977500
H	0.41688600	-5.13420700	1.35158700
H	-0.28388000	-4.98701000	-0.29454100

[Co-H]$^{+}$

	x	y	z
C	-1.16360400	-0.44638400	2.82589900
C	-0.22344000	-1.52798200	2.90435600
C	1.09944400	-0.97259500	2.84814500
C	0.96830500	0.46455600	2.76781300
C	-0.42232100	0.79296000	2.75069500
H	-2.25073400	-0.53972300	2.84097400
H	-0.46808200	-2.58863800	2.97355300
H	2.03470100	-1.53311600	2.86378800
H	1.79794100	1.17223700	2.72573900
H	-0.85631500	1.79260000	2.69915500
Co	0.04937600	-0.47508200	1.10125300
H	0.11499500	-1.79838700	0.42977800
C	0.71816800	-0.45622800	-2.04358300
H	1.34654300	-0.12152200	-2.88788400
H	0.57615600	-1.54755400	-2.13119500
C	-0.63359700	0.27457900	-2.01957600
H	-1.27582500	-0.02891200	-2.86466700
H	-0.48044400	1.36611400	-2.08986200
P	1.56638100	-0.10058700	-0.41363400
P	-1.50249300	-0.08774300	-0.39128700
C	3.05101700	-1.18264700	-0.31811900
C	2.94843500	-2.54758500	-0.67088600
C	4.27348700	-0.69154500	0.19308900
C	4.05826900	-3.39743200	-0.54337700
H	2.00212900	-2.95786400	-1.04189100
C	5.37828900	-1.54928600	0.32867200
H	4.37208500	0.36194400	0.47734200
C	5.27499500	-2.90056300	-0.04232600
H	3.97073500	-4.45035400	-0.83315600
H	6.32221600	-1.15638000	0.72244300
H	6.13924300	-3.56593300	0.05920000
C	2.22324600	1.60871100	-0.66439400
C	1.71300800	2.69295000	0.07875500
C	3.19898300	1.85259600	-1.66048600
C	2.17382400	4.00039000	-0.15920500
H	0.95106900	2.51037500	0.84376200
C	3.65476000	3.15881600	-1.89781600
H	3.61536100	1.02294000	-2.24410500
C	3.14470500	4.23415200	-1.14679900
H	1.77485200	4.83429400	0.42918700

H	4.41310100	3.33648800	-2.66836400
H	3.50634200	5.25150900	-1.33200000
C	-2.63967600	1.33557700	-0.09932100
C	-2.18000700	2.66164100	-0.27210100
C	-3.95254300	1.11987100	0.37730900
C	-3.02772400	3.74777800	-0.00164500
H	-1.15920300	2.85725300	-0.62096200
C	-4.79335100	2.21067100	0.65655400
H	-4.32894900	0.10049700	0.51668600
C	-4.33572900	3.52519900	0.46482000
H	-2.66494000	4.77003400	-0.15661000
H	-5.81136200	2.02878800	1.01849600
H	-4.99567200	4.37373600	0.67551000
C	-2.62471800	-1.50055600	-0.78611500
C	-3.55660900	-1.38961300	-1.84548100
C	-2.55629200	-2.70148600	-0.05140800
C	-4.39390500	-2.46958300	-2.16504400
H	-3.64210200	-0.45736200	-2.41682200
C	-3.39959700	-3.78014600	-0.37118200
H	-1.83248000	-2.78985600	0.76538400
C	-4.31649000	-3.66669900	-1.42891700
H	-5.10991100	-2.37504100	-2.98894700
H	-3.33708900	-4.70911000	0.20642700
H	-4.97183100	-4.50761800	-1.68093500

[Co-CO]$^{2+}$

	x	y	z
C	-0.78183900	-0.17199200	3.13726900
C	0.66602300	-0.24979200	3.17159700
C	1.18798800	0.91659800	2.53396800
C	0.07434700	1.71002400	2.07449000
C	-1.13944400	1.04624800	2.48458200
H	-1.47031400	-0.91058800	3.55214700
H	1.24732900	-1.05866800	3.61897200
H	2.24301800	1.16522000	2.40707200
H	0.14836400	2.67747300	1.57781800
H	-2.15348400	1.40758700	2.30713700
Co	-0.01220400	-0.17489200	1.13031300
C	0.67907400	-0.27094300	-2.08950200
H	1.28098300	0.12575200	-2.92665500
H	0.62489400	-1.36883800	-2.20593700
C	-0.71922700	0.35672400	-2.08359900
H	-1.32939600	0.00303300	-2.93445300
H	-0.67479000	1.45445500	-2.18260900
P	1.59164200	0.02160700	-0.48074700
P	-1.64016200	0.02966100	-0.49344900
C	2.90400300	-1.25105900	-0.41644300
C	3.28622400	-1.86088200	0.79896600
C	3.59358000	-1.57724900	-1.61032300
C	4.33454600	-2.79529100	0.82086000
H	2.76772700	-1.61360900	1.73116800
C	4.63586600	-2.51661600	-1.58034500
H	3.33557000	-1.09961900	-2.56184800
C	5.00674800	-3.12626800	-0.36811300
H	4.62360500	-3.26623000	1.76629900
H	5.16112000	-2.76827700	-2.50778500
H	5.82085300	-3.85841800	-0.35111900
C	2.45455000	1.63637500	-0.58643400
C	3.77852400	1.74039500	-0.09424000
C	1.83072800	2.77675100	-1.14874000
C	4.46487800	2.96306500	-0.17621400
H	4.28660100	0.86445000	0.32346700
C	2.52832200	3.99109700	-1.23637300
H	0.81240600	2.72615800	-1.54790900
C	3.84364000	4.08819800	-0.74531400
H	5.49318800	3.02970000	0.19467100
H	2.04680700	4.86138900	-1.69510400
H	4.38476600	5.03770100	-0.81429000
C	-2.86026400	1.39254700	-0.36932200
C	-2.39853000	2.73161300	-0.31915100
C	-4.25097700	1.13390600	-0.39399300
C	-3.31411000	3.79292100	-0.30066800
H	-1.32429400	2.95233700	-0.30068900
C	-5.16061200	2.20534800	-0.37329600
H	-4.62894100	0.10879400	-0.44953800
C	-4.69780500	3.53042300	-0.32389800
H	-2.95056200	4.82561100	-0.27002700
H	-6.23541100	1.99749400	-0.40306100
H	-5.41168100	4.36062300	-0.30704900
C	-2.56995200	-1.53508000	-0.65136900
C	-2.53995200	-2.31155100	-1.83233100
C	-3.32932400	-1.98135200	0.45925400
C	-3.27195800	-3.50910100	-1.90216100
H	-1.96296200	-1.99652200	-2.70777100
C	-4.06246400	-3.17336200	0.37804700
H	-3.36085300	-1.39246200	1.38400200
C	-4.03258700	-3.94019700	-0.80221500
H	-3.24851600	-4.10207100	-2.82246500
H	-4.65768700	-3.50518900	1.23533700
H	-4.60271600	-4.87308600	-0.86304800
C	-0.00993100	-1.93071000	0.96361800
O	0.00141400	-3.08698200	0.91701700

L-L = en
M = Mn

[Mn-S]^{0}

	x	y	z
C	1.97187600	0.72504800	-1.16760300
C	1.36322300	1.87106700	-0.55042700
C	1.48045400	1.73350900	0.88024100
C	2.14538200	0.49006500	1.13507200
C	2.48506700	-0.13647700	-0.11423100
H	2.08144000	0.56698600	-2.24534700
H	0.86301800	2.68828000	-1.07515100
H	1.10491700	2.43045100	1.63231600
H	2.36130000	0.08404500	2.12929700
H	3.06304700	-1.05638000	-0.23989400
Mn	0.36983600	0.10297500	0.00028600
C	-1.17054700	-2.31704300	0.70261300
H	-1.33108400	-3.22235300	1.32235800
H	-2.15508300	-1.86621100	0.48377500
C	-0.46418500	-2.66708800	-0.60753700
H	-1.07456700	-3.37432900	-1.20619800
H	0.51284400	-3.14135000	-0.39983500
N	-0.35414700	-1.27640900	1.38883900
N	-0.19468600	-1.38450200	-1.31466700
H	-0.92610200	-0.76616800	2.07279300
H	0.49344600	-1.52345700	-2.06523400
H	-1.06039200	-1.03175800	-1.75772600
H	0.41940800	-1.71238500	1.90867900
N	-1.27219300	0.79367700	-0.17030800

C	-2.38383500	1.19180700	-0.46993300
C	-3.60160700	1.83851700	0.09166300
H	-3.48744000	2.07051600	1.17369400
H	-3.82854500	2.78368300	-0.43653000
H	-4.48987900	1.19026400	-0.03034800

$[Mn\text{-}H]^{-1}$

	x	y	z
C	-1.69108800	1.23514700	0.32161000
C	-2.11865900	0.47357400	-0.83765900
C	-2.06495300	-0.93678900	-0.48402600
C	-1.57523200	-1.00221900	0.87612800
C	-1.40586800	0.32511300	1.41763600
H	-1.63134800	2.33236900	0.37382700
H	-2.44644600	0.88858400	-1.79580500
H	-2.34669600	-1.78483600	-1.11665400
H	-1.37178000	-1.93464900	1.42488100
H	-1.16381700	0.58948100	2.45444200
Mn	-0.19742100	-0.01530200	-0.32737100
H	0.28161000	-0.19134100	-1.91489400
C	2.62824900	-0.71444200	-0.10840300
H	3.45809400	-1.25190600	0.40953300
H	2.84572500	-0.69095900	-1.19265600
C	2.52649200	0.72181300	0.41714000
H	3.48797200	1.26359700	0.23231600
H	2.33645100	0.71207200	1.50829900
N	1.30872000	-1.36779800	0.06266300
N	1.35012200	1.35598300	-0.23347900
H	1.16483800	-2.09164800	-0.65086700
H	1.10620900	2.23280800	0.25050900
H	1.56922800	1.58527600	-1.21448700
H	1.23993600	-1.80677700	0.99699000

$[Mn\text{-}CO]^{0}$

	x	y	z
C	1.71946300	-0.74164000	-1.18733000
C	2.23528300	0.38662100	-0.46075500
C	2.14253800	0.10560500	0.95429100
C	1.56268100	-1.19617800	1.08534100
C	1.32015500	-1.74565600	-0.21691000
H	1.71494700	-0.84980100	-2.27568400
H	2.62990400	1.30283300	-0.90426500
H	2.45805500	0.76098900	1.76779100
H	1.33230900	-1.68916600	2.03562200
H	0.93136200	-2.74471200	-0.43116200
Mn	0.20589400	0.09720100	-0.00812400
C	-2.64262300	-0.18569800	0.69737600
H	-3.45681500	-0.61252200	1.31768800
H	-2.87697400	0.87454800	0.49745800
C	-2.50288700	-0.95147500	-0.61861900
H	-3.43679400	-0.89115000	-1.21306500
H	-2.29835500	-2.01862400	-0.41176600
N	-1.31543400	-0.22418100	1.37423400
N	-1.31728700	-0.40089800	-1.33738400
H	-1.25983600	0.50327600	2.09594000
H	-0.97440000	-1.07649800	-2.03209900
H	-1.57459600	0.44741800	-1.85539100
H	-1.18994800	-1.12751400	1.85244800
C	-0.06530700	1.82029700	-0.10363500
O	-0.16662100	3.01439900	-0.17461300

L-L = en

M = Fe

$[Fe\text{-}S]^{+}$

	x	y	z
C	1.72214600	1.18856800	-1.17704100
C	0.79499600	2.10108400	-0.59215800
C	0.89820300	1.98780200	0.84749500
C	1.89916700	1.00532500	1.12588200
C	2.41368200	0.48955000	-0.11497900
H	1.86954600	1.03976700	-2.24956300
H	0.10388900	2.74548200	-1.13794100
H	0.31934700	2.54623400	1.58453100
H	2.19625200	0.67587100	2.12530300
H	3.21908000	-0.23838400	-0.23069100
Fe	0.35603900	0.13649900	0.00420200
C	-0.45932600	-2.56988900	0.68695800
H	-0.34006500	-3.48217800	1.29973600
H	-1.53848500	-2.39208600	0.53989800
C	0.24647400	-2.70548700	-0.65855200
H	-0.19126000	-3.52107000	-1.26328100
H	1.31829700	-2.92572200	-0.51097500
N	0.10702700	-1.35970100	1.35861700
N	0.15117000	-1.37824100	-1.34359200
H	-0.50868700	-1.05418300	2.12436500
H	0.84665100	-1.32094800	-2.09884000
H	-0.76876100	-1.28233400	-1.79795100
H	1.01106100	-1.59352500	1.79205300
N	-1.48491100	0.43541900	-0.01993300
C	-2.63427100	0.70444300	-0.04618100
C	-4.04117300	1.09435300	-0.07333100
H	-4.27957700	1.74051400	0.79091100
H	-4.27190500	1.65174200	-0.99925000
H	-4.69478300	0.20500400	-0.03176700

$[Fe\text{-}H]^{0}$

	x	y	z
C	-1.68390100	1.22200800	0.37185300
C	-2.05672200	0.55922300	-0.84946700
C	-2.02801500	-0.86750700	-0.59598600
C	-1.63497000	-1.04618300	0.77922700
C	-1.45393400	0.23867000	1.40687800
H	-1.59083000	2.30691700	0.49188000
H	-2.30434100	1.04464800	-1.79489800
H	-2.25935200	-1.65933600	-1.31060800
H	-1.49691100	-2.01634300	1.26923600
H	-1.24104900	0.43113800	2.46132400
Fe	-0.17857200	-0.03091300	-0.27839100
H	0.24392200	-0.20832800	-1.77293600
C	2.62428600	-0.70197100	-0.15115100
H	3.46950600	-1.24903200	0.31368000
H	2.76940500	-0.69128800	-1.24526500
C	2.54602900	0.73239600	0.37605000
H	3.46737800	1.29608600	0.12233500
H	2.43977500	0.72571600	1.47658600
N	1.30629200	-1.34428800	0.10745700
N	1.31182800	1.34683900	-0.18693400
H	1.17345900	-2.14825500	-0.51657100
H	1.04107200	2.17688900	0.35550000
H	1.46786000	1.65123400	-1.15550100
H	1.25949700	-1.69399300	1.07531100

[Fe-CO]$^{+}$

	x	y	z
C	1.68013100	-0.96989700	-1.10320000
C	2.18287800	0.29859300	-0.68431900
C	2.14346300	0.33236000	0.76535900
C	1.62529700	-0.92241700	1.21121700
C	1.32624000	-1.73772600	0.06527000
H	1.57965400	-1.29412400	-2.14174000
H	2.53304200	1.09907900	-1.33790700
H	2.46604400	1.15923400	1.39984300
H	1.46254100	-1.19897100	2.25594400
H	0.96118400	-2.76635100	0.07891400
Fe	0.18538400	0.06525400	-0.00859500
C	-2.61902600	-0.16547700	0.68657100
H	-3.41475400	-0.58125800	1.33076500
H	-2.85337500	0.89460400	0.48904500
C	-2.48789200	-0.94795600	-0.61518500
H	-3.39971000	-0.86489900	-1.23319200
H	-2.31311500	-2.01791600	-0.40535900
N	-1.27981700	-0.21712000	1.35500100
N	-1.27942500	-0.42424400	-1.33307400
H	-1.23122200	0.47793000	2.11221800
H	-0.94813500	-1.12115600	-2.01317700
H	-1.53240300	0.40416600	-1.88841000
H	-1.15406100	-1.13580800	1.80394900
C	-0.08906200	1.79346000	-0.12352400
O	-0.20414600	2.95659900	-0.19925700

L-L = en
M = Co

[Co-S]$^{2+}$

	x	y	z
C	-1.72342500	-1.23406700	-1.17305600
C	-0.74852500	-2.11195100	-0.62056400
C	-0.82188800	-2.01438400	0.82693100
C	-1.85901300	-1.08729100	1.14658000
C	-2.39172100	-0.55347000	-0.08280800
H	-1.90737800	-1.08030300	-2.23932400
H	-0.05014100	-2.73083700	-1.18769300
H	-0.20261100	-2.56033200	1.54159700
H	-2.15849300	-0.79383300	2.15631800
H	-3.21132900	0.16317700	-0.17323400
Co	-0.35813300	-0.11567400	0.00397700
C	0.40013000	2.56977100	0.68313800
H	0.26111500	3.46485900	1.31485100
H	1.48190300	2.41050200	0.54300400
C	-0.31781800	2.70336800	-0.65228900
H	0.11677500	3.50600700	-1.27393600
H	-1.38925400	2.92456300	-0.50878900
N	-0.15966200	1.34777800	1.35604400
N	-0.21489700	1.37136200	-1.34456300
H	0.45250300	1.04430300	2.12852300
H	-0.90686900	1.31603900	-2.10585900
H	0.70695800	1.27467200	-1.79757700
H	-1.06919300	1.57638800	1.78501200
N	1.54492700	-0.39254300	-0.02356900
C	2.68683900	-0.66242200	-0.04654200
C	4.09976600	-1.00688100	-0.07474800
H	4.34196100	-1.67852400	0.76906500
H	4.34588400	-1.52029700	-1.02214600
H	4.71911500	-0.09539200	0.00757900

[Co-H]$^{+}$

	x	y	z
C	-1.84463800	1.14592400	-0.26943800
C	-2.06277400	-0.06981300	-1.00132200
C	-1.86305200	-1.16576800	-0.09286400
C	-1.61549600	-0.61199100	1.22771800
C	-1.60425700	0.79791400	1.12180700
H	-1.90865800	2.15574600	-0.67941400
H	-2.29613200	-0.14816700	-2.06349400
H	-1.94900600	-2.22508600	-0.34183500
H	-1.44497100	-1.18828200	2.13993900
H	-1.42897700	1.50233700	1.93785400
Co	-0.13394800	-0.03188000	-0.25936500
H	0.20505000	-0.20644300	-1.69912100
C	2.63326200	-0.69280800	-0.13307800
H	3.45645300	-1.25252300	0.34599500
H	2.79982400	-0.69283800	-1.22351200
C	2.54335800	0.73477300	0.39893700
H	3.44383400	1.32057300	0.14057400
H	2.43746900	0.73518500	1.49800300
N	1.30525100	-1.34002000	0.11704700
N	1.30027700	1.34363900	-0.17575100
H	1.19764200	-2.17218900	-0.47776600
H	1.02293100	2.17205700	0.36820800
H	1.47520600	1.67510800	-1.13424000
H	1.24881600	-1.66943100	1.09201700

[Co-CO]$^{2+}$

	x	y	z
C	1.72782500	-0.93627600	-1.10679200
C	2.17350900	0.35574000	-0.68876500
C	2.12715900	0.39153200	0.76710400
C	1.66888600	-0.88781300	1.21811300
C	1.38285300	-1.70420700	0.06703500
H	1.65095100	-1.27255200	-2.14451200
H	2.50608300	1.16574300	-1.34370700
H	2.42650900	1.23056200	1.40092800
H	1.52987100	-1.17656100	2.26393100
H	1.04801400	-2.74467800	0.08111600
Co	0.17283300	0.04824200	-0.00657900
C	-2.60912100	-0.19988200	0.68103500
H	-3.38798400	-0.61951400	1.34259800
H	-2.86175300	0.85563800	0.48278400
C	-2.46800100	-1.00062000	-0.60486500
H	-3.36768400	-0.92103400	-1.24029000
H	-2.29243400	-2.06913100	-0.39257700
N	-1.26548700	-0.23967100	1.36046100
N	-1.25783800	-0.47204700	-1.33459100
H	-1.22416300	0.44964000	2.12789300
H	-0.92494400	-1.17350700	-2.01282100
H	-1.51752100	0.34860100	-1.90385700
H	-1.13221800	-1.16165600	1.80490200
C	-0.14583100	1.82778500	-0.13262000
O	-0.32495200	2.96129800	-0.20891200

2. Pincer Systems: [(PCP)M(L-L)]$^{n+}$

L-L = bis CO
M = Mn

[Mn-S]^{0}

	x	y	z
C	-1.19575100	2.00790000	-0.11460800
C	0.00000800	1.27024300	-0.15718100
C	1.19578700	2.00785200	-0.11441000
C	1.22693100	3.41133600	-0.02451400
C	0.00004800	4.09995200	0.01820000
C	-1.22685600	3.41138500	-0.02471900
H	2.18302700	3.94426800	0.00474900
H	0.00006400	5.19431500	0.08289100
H	-2.18293600	3.94435300	0.00439100
O	-2.41220800	1.31388000	-0.16104100
O	2.41222600	1.31378800	-0.16064900
P	-2.19820100	-0.37884600	-0.24011300
P	2.19817200	-0.37890100	-0.24035100
Mn	-0.00002300	-0.78492900	-0.24538400
C	-3.26531300	-0.77812400	-1.69203300
H	-3.36912500	-1.87430400	-1.77961000
H	-4.26388700	-0.32236300	-1.57482800
H	-2.79695100	-0.39607900	-2.61343100
C	-3.29413600	-0.89910800	1.15828800
H	-4.28513400	-0.42069700	1.06951000
H	-3.41246000	-1.99704700	1.14203400
H	-2.83781100	-0.60607400	2.11731200
C	3.29432500	-0.89970300	1.15767300
H	3.41265900	-1.99763300	1.14095700
H	4.28530400	-0.42124200	1.06894200
H	2.83813000	-0.60706800	2.11688000
C	3.26503900	-0.77766700	-1.69259600
H	4.26369800	-0.32211600	-1.57529900
H	3.36866600	-1.87382200	-1.78071000
H	2.79661600	-0.39511300	-2.61375300
C	-0.00013700	-0.69379700	-2.01080000
O	-0.00025700	-0.62639900	-3.18936300
C	-0.00006400	-2.58105000	-0.27674300
O	-0.00012300	-3.76141300	-0.31750800
N	0.00012200	-0.73934000	1.72499800
C	0.00011900	-0.67161100	2.89732000
C	0.00033300	-0.57018700	4.35367800
H	0.89781500	-1.05504100	4.77813200
H	-0.00440500	0.49043300	4.66260500
H	-0.89221800	-1.06334600	4.77896300

[Mn-H]$^{-1}$

	x	y	z
C	1.19227500	1.95942600	-0.02707000
C	0.00004800	1.20960900	-0.01715300
C	-1.19212300	1.95951500	-0.02712200
C	-1.22470200	3.36791400	-0.00270500
C	0.00015300	4.06435100	0.01738400
C	1.22495800	3.36782300	-0.00265100
H	-2.18379300	3.89940400	-0.00383000
H	0.00019400	5.16203800	0.03802000
H	2.18408900	3.89924200	-0.00373700
O	2.40235200	1.26141900	-0.07585600
O	-2.40225000	1.26159900	-0.07595600
P	2.12398200	-0.43870100	-0.21609100
P	-2.12401100	-0.43855000	-0.21608500
Mn	-0.00002800	-0.84406900	-0.03121400
C	3.39622200	-1.00617900	1.01779200
H	3.53243200	-2.09885900	0.92096500
H	4.36557200	-0.49785900	0.86065700
H	3.02640100	-0.79094900	2.03408900
C	3.05810800	-0.75433800	-1.79439400
H	4.08874000	-0.35526000	-1.75143100
H	3.08681100	-1.84333600	-1.98220400
H	2.50546500	-0.27589900	-2.61969700
C	-3.05819000	-0.75422000	-1.79434900
H	-3.08697100	-1.84322800	-1.98208900
H	-4.08879400	-0.35506900	-1.75139000
H	-2.50553100	-0.27587100	-2.61969500
C	-3.39627100	-1.00585800	1.01785800
H	-4.36558700	-0.49748000	0.86070500
H	-3.53255900	-2.09853500	0.92110800
H	-3.02641700	-0.79058500	2.03413400
C	-0.00002200	-1.03814000	1.73489500
O	-0.00006000	-1.19913500	2.91697500
C	-0.00009400	-2.57016800	-0.45119200
O	-0.00017600	-3.71729400	-0.77703600
H	-0.00002900	-0.62839300	-1.62539700

[Mn-CO]^{0}

	x	y	z
C	-1.19772200	1.98486800	-0.00011500
C	0.00000000	1.25364300	0.00009000
C	1.19772700	1.98486000	0.00010300
C	1.22754400	3.39010400	-0.00025300
C	0.00001000	4.07850300	-0.00054800
C	-1.22752900	3.39011300	-0.00047700
H	2.18323800	3.92377700	-0.00024400
H	0.00001400	5.17437200	-0.00080400
H	-2.18321900	3.92379300	-0.00063900
O	-2.40940400	1.28163900	0.00024900
O	2.40940400	1.28162100	0.00069900
P	-2.20557900	-0.40742500	-0.00045200
P	2.20556700	-0.40744100	-0.00057200
Mn	-0.00000900	-0.81268900	0.00065500
C	-3.27140700	-0.87976300	-1.43025700
H	-3.36022300	-1.97985600	-1.47279600
H	-4.27592400	-0.43534000	-1.32099000
H	-2.81891600	-0.52558400	-2.37042900
C	-3.27307200	-0.88133400	1.42756500
H	-4.27748600	-0.43675700	1.31794100
H	-3.36195200	-1.98149100	1.46846200
H	-2.82150200	-0.52849200	2.36869000
C	3.27324300	-0.88182800	1.42714200
H	3.36223200	-1.98199400	1.46756700
H	4.27760400	-0.43711200	1.31759700
H	2.82173900	-0.52942100	2.36846200
C	3.27120400	-0.87931500	-1.43068000
H	4.27578400	-0.43505500	-1.32133200
H	3.35988600	-1.97940100	-1.47369700
H	2.81865600	-0.52468500	-2.37065500

C	-0.00009500	-0.71410400	-1.81311300
O	-0.00015100	-0.63380300	-2.98279600
C	-0.00001300	-2.60819400	0.00229500
O	-0.00005200	-3.78576100	0.00364200
C	0.00005400	-0.71055800	1.81441800
O	0.00030300	-0.62725600	2.98381200

L-L = bis CO
M = Fe

[Fe-S]$^+$

	x	y	z
C	-1.20138800	1.98777100	-0.03313800
C	-0.00038200	1.26475900	-0.10888700
C	1.20028700	1.98831200	-0.03292200
C	1.22777800	3.38203600	0.12539000
C	-0.00104300	4.06340000	0.19972300
C	-1.22954200	3.38148300	0.12516200
H	2.18199400	3.91400200	0.18220300
H	-0.00129900	5.15210800	0.31654800
H	-2.18401000	3.91301600	0.18180800
O	-2.41457100	1.28773700	-0.11301700
O	2.41381300	1.28884200	-0.11254600
P	-2.23265000	-0.37726700	-0.25459400
P	2.23271900	-0.37616900	-0.25509600
Fe	0.00011400	-0.73553400	-0.27421400
C	-3.24509000	-0.76800700	-1.73374300
H	-3.30275300	-1.86274100	-1.86757200
H	-4.26409500	-0.36590700	-1.59902900
H	-2.80048600	-0.31975000	-2.63650200
C	-3.25173700	-1.01471100	1.13455600
H	-4.27081600	-0.59745000	1.05940800
H	-3.30469300	-2.11624600	1.08249400
H	-2.81341300	-0.71752600	2.09925400
C	3.25265500	-1.01386600	1.13330200
H	3.30649700	-2.11531500	1.08034500
H	4.27137600	-0.59572100	1.05820400
H	2.81435700	-0.71781800	2.09836100
C	3.24483100	-0.76556200	-1.73483700
H	4.26377100	-0.36329900	-1.60012700
H	3.30273600	-1.86017900	-1.86951300
H	2.79984200	-0.31674100	-2.63712600
C	-0.00019600	-0.44491700	-1.99502600
O	-0.00049500	-0.22984000	-3.14081200
C	0.00049100	-2.52817500	-0.41337900
O	0.00137300	-3.68754000	-0.52165800
N	0.00040900	-0.79569700	1.68699700
C	0.00068200	-0.78586000	2.85722100
C	0.00099000	-0.75674000	4.31518100
H	0.89652800	-1.26879400	4.70897900
H	0.00155900	0.28840200	4.67154900
H	-0.89492300	-1.26788900	4.70931800

[Fe-H]^0

	x	y	z
C	1.19383100	1.93704700	-0.04427200
C	-0.00001300	1.19948400	-0.03088500
C	-1.19385800	1.93703900	-0.04468000
C	-1.22581300	3.34146600	-0.04233800
C	-0.00001900	4.03254500	-0.03183400
C	1.22578400	3.34147300	-0.04192500
H	-2.18261100	3.87303300	-0.04953700
H	-0.00002300	5.12833200	-0.02820600
H	2.18258200	3.87304300	-0.04881500
O	2.40164600	1.22625800	-0.07240400
O	-2.40165800	1.22625700	-0.07321500
P	2.14349800	-0.44186100	-0.22742400
P	-2.14353700	-0.44194900	-0.22738000
Fe	-0.00000500	-0.80772700	-0.00423000
C	3.36310400	-1.08269300	0.99635200
H	3.42686300	-2.18179800	0.90871900
H	4.36002500	-0.64322400	0.81698700
H	3.03044500	-0.82850600	2.01583800
C	2.96357400	-0.80772700	-1.83670700
H	4.00744800	-0.44816200	-1.83670200
H	2.94837700	-1.89752000	-2.01582000
H	2.40016300	-0.31155200	-2.64338100
C	-2.96390600	-0.80861800	-1.83632500
H	-2.94893500	-1.89852100	-2.01479300
H	-4.00772100	-0.44887900	-1.83635300
H	-2.40055400	-0.31301500	-2.64338900
C	-3.36297100	-1.08216100	0.99690700
H	-4.35994000	-0.64285400	0.81741300
H	-3.42667100	-2.18132000	0.90991400
H	-3.03020200	-0.82737100	2.01620700
C	0.00003200	-0.86857900	1.76910100
O	0.00004500	-0.90485200	2.94133000
C	0.00002000	-2.53395100	-0.36273900
O	0.00034000	-3.67261300	-0.65165400
H	-0.00010100	-0.62598500	-1.53241500

[Fe-CO]$^+$

	x	y	z
C	-1.20496700	1.95394200	0.00009700
C	0.00011900	1.23699100	0.00027800
C	1.20530100	1.95377900	0.00027900
C	1.23079900	3.35546900	0.00049700
C	0.00030800	4.03867200	0.00055800
C	-1.23027600	3.35563600	0.00031100
H	2.18402000	3.89185500	0.00056000
H	0.00038100	5.13338900	0.00075100
H	-2.18342400	3.89215000	0.00023100
O	-2.41145500	1.23949200	-0.00050000
O	2.41168700	1.23916400	-0.00010100
P	-2.24423100	-0.42889400	-0.00017000
P	2.24422600	-0.42920000	-0.00037100
Fe	-0.00002500	-0.78424700	0.00027800
C	-3.24196000	-0.95692900	-1.44416200
H	-3.27630700	-2.05994100	-1.48781300
H	-4.26940800	-0.56792900	-1.33587000
H	-2.81000600	-0.57141200	-2.38116400
C	-3.24269900	-0.95633800	1.44351900
H	-4.27029400	-0.56806500	1.33402600
H	-3.27636800	-2.05931800	1.48826800
H	-2.81167900	-0.56954900	2.38043600
C	3.24284700	-0.95725800	1.44298000
H	3.27650200	-2.06025600	1.48725800
H	4.27043600	-0.56895700	1.33352400
H	2.81194800	-0.57085500	2.38011100
C	3.24166400	-0.95688900	-1.44469700

H	4.26922300	-0.56819500	-1.33636200
H	3.27572500	-2.05989100	-1.48881400
H	2.80969900	-0.57087000	-2.38148500
C	-0.00007800	-0.58568300	-1.78682200
O	-0.00074100	-0.41535200	-2.93471400
C	-0.00011700	-2.57845400	-0.00000300
O	-0.00038100	-3.74043300	-0.00029500
C	0.00013100	-0.58701300	1.78788800
O	0.00012000	-0.41735300	2.93597400

L-L = bis CO
M = Co

[Co-S]$^{2+}$

	x	y	z
C	-1.24310300	1.96045300	0.45151800
C	-0.00005800	1.36935500	0.02284200
C	1.24292500	1.96053400	0.45158500
C	1.24615000	3.16480500	1.14938500
C	-0.00018100	3.75034100	1.47009300
C	-1.24645700	3.16473900	1.14928800
H	2.18785300	3.63164400	1.45192900
H	-0.00022700	4.69571500	2.02398000
H	-2.18821200	3.63152700	1.45174800
O	-2.40826300	1.32931700	0.10519600
O	2.40815200	1.32948100	0.10534200
P	-2.27753800	-0.31496800	-0.33923300
P	2.27758300	-0.31481600	-0.33909800
Co	0.00004100	-0.60686300	-0.37469600
C	-3.23561300	-0.39089600	-1.88618700
H	-3.37192700	-1.44697200	-2.18135300
H	-4.22845200	0.06579800	-1.72329700
H	-2.71438500	0.14558700	-2.69581500
C	-3.26214000	-1.16401500	0.94311900
H	-4.29295500	-0.76735600	0.92077500
H	-3.28626000	-2.24703200	0.72730300
H	-2.82483500	-0.99423600	1.93892400
C	3.26212800	-1.16381000	0.94332700
H	3.28630200	-2.24682900	0.72752700
H	4.29292900	-0.76711100	0.92107200
H	2.82472400	-0.99403200	1.93909100
C	3.23574200	-0.39065300	-1.88600200
H	4.22853800	0.06611500	-1.72306500
H	3.37215000	-1.44671300	-2.18118200
H	2.71449900	0.14580200	-2.69564000
C	0.00001200	0.82584700	-1.45895600
O	0.00004300	1.31755800	-2.54203500
C	0.00016500	-2.03229000	-1.46148400
O	0.00034300	-2.91365700	-2.20766500
N	0.00001400	-1.54394300	1.41570600
C	-0.00003500	-2.14932500	2.41919000
C	-0.00011400	-2.90686900	3.66367000
H	0.89797200	-3.54782300	3.71919300
H	-0.00406000	-2.22170700	4.53007300
H	-0.89426100	-3.55361200	3.71551900

[Co-H]$^{+}$

	x	y	z
C	1.20080300	1.91819400	-0.06284000
C	0.00010400	1.19449900	-0.04233200
C	-1.20045500	1.91841100	-0.06323700
C	-1.22723200	3.31968000	-0.07761100
C	0.00037000	4.00603300	-0.07718400
C	1.22784800	3.31945600	-0.07720900
H	-2.18234700	3.85250300	-0.08869000
H	0.00047100	5.10057900	-0.08745100
H	2.18306400	3.85210300	-0.08799300
O	2.40646800	1.20251700	-0.06468900
O	-2.40625200	1.20297700	-0.06549000
P	2.19580900	-0.44481800	-0.24234100
P	-2.19592300	-0.44449700	-0.24226400
Co	-0.00007900	-0.78237200	0.01487900
C	3.36749600	-1.13721300	0.98055000
H	3.39153300	-2.23763500	0.89341700
H	4.37864600	-0.73769700	0.78656800
H	3.06102400	-0.86115100	2.00222000
C	2.91866300	-0.83208100	-1.87934400
H	3.96766200	-0.48937100	-1.91141500
H	2.88707600	-1.92174600	-2.05604800
H	2.34176600	-0.32294400	-2.66824200
C	-2.91915600	-0.83244900	-1.87893100
H	-2.88801500	-1.92223000	-2.05499700
H	-3.96803300	-0.48936800	-1.91103300
H	-2.34220400	-0.32398900	-2.66822400
C	-3.36754500	-1.13606600	0.98117000
H	-4.37866800	-0.73651400	0.78711700
H	-3.39174400	-2.23653100	0.89464300
H	-3.06088900	-0.85948900	2.00264500
C	-0.00000700	-0.73144300	1.82805000
O	0.00007500	-0.64645800	2.98116500
C	-0.00019400	-2.54836800	-0.29242700
O	-0.00023300	-3.67953300	-0.54281400
H	-0.00013700	-0.59069400	-1.46248500

[Co-CO]$^{2+}$

	x	y	z
C	-1.21670200	1.93000600	0.00002500
C	-0.00000500	1.22717100	0.00005300
C	1.21668900	1.93001000	0.00002400
C	1.23353900	3.32935400	0.00009800
C	-0.00001000	4.00899900	0.00015200
C	-1.23355600	3.32935000	0.00009700
H	2.18560700	3.86865200	0.00006800
H	-0.00001100	5.10360700	0.00020400
H	-2.18562600	3.86864400	0.00006800
O	-2.41934700	1.21420900	-0.00023800
O	2.41933800	1.21421800	-0.00023400
P	-2.32302600	-0.44119400	-0.00006000
P	2.32302500	-0.44118500	-0.00013700
Co	-0.00000200	-0.76457200	0.00001000
C	-3.26277000	-0.99463500	-1.46218400
H	-3.28431400	-2.09855400	-1.50233100
H	-4.29936800	-0.62268700	-1.35833700
H	-2.83504800	-0.59643800	-2.39592300
C	-3.26222100	-0.99438800	1.46255400
H	-4.29952400	-0.62462400	1.35804200
H	-3.28162300	-2.09827700	1.50446500
H	-2.83538400	-0.59387900	2.39571900
C	3.26226900	-0.99444100	1.46242300
H	3.28166700	-2.09833200	1.50429100
H	4.29957000	-0.62467800	1.35789200

H	2.83546200	-0.59396500	2.39561800
C	3.26272300	-0.99456000	-1.46231700
H	4.29932600	-0.62262500	-1.35848000
H	3.28425700	-2.09847600	-1.50252100
H	2.83497700	-0.59631100	-2.39602200
C	-0.00003800	-0.48564800	-1.78410600
O	-0.00015700	-0.27043000	-2.91577200
C	0.00000600	-2.60207300	-0.00013000
O	0.00000300	-3.75106700	-0.00022100
C	0.00002800	-0.48595900	1.78413600
O	0.00021200	-0.27093400	2.91583600

L-L = bpy
M = Mn

[Mn-S]^0

	x	y	z
C	2.62879700	-0.61443900	-1.18676200
C	1.90305000	-0.36821300	0.00094500
C	2.62485300	-0.62266700	1.18930200
C	3.94797700	-1.09828100	1.22469600
C	4.60520800	-1.33227800	0.00210300
C	3.95204000	-1.08983200	-1.22102100
H	4.44445000	-1.27757700	2.18459800
H	5.63668900	-1.70397000	0.00253400
H	4.45167500	-1.26260800	-2.18047900
O	1.99019300	-0.37748900	-2.41350600
O	1.98209500	-0.39417000	2.41549200
P	0.41278400	0.23978600	-2.19298600
P	0.40832400	0.23182700	2.19369300
Mn	-0.01140000	0.35452000	0.00015700
C	-0.52734500	-0.90538000	-3.30744800
H	-1.55751700	-0.52739900	-3.43839200
H	-0.03773800	-0.98161300	-4.29444100
H	-0.57821300	-1.90780000	-2.85151600
C	0.52468800	1.74275600	-3.27384500
H	0.93208700	1.48236000	-4.26662100
H	-0.47966500	2.18759600	-3.39396900
H	1.18380800	2.48670600	-2.79765400
C	0.52570900	1.73225100	3.27756700
H	-0.47658300	2.18196300	3.39683500
H	0.93020200	1.46804000	4.27051700
H	1.18931500	2.47369400	2.80366900
C	-0.53910400	-0.91110200	3.30411600
H	-0.05172800	-0.99151500	4.29188400
H	-1.56769500	-0.52845500	3.43389400
H	-0.59383900	-1.91241500	2.84619400
C	-0.17143000	-2.65382100	-0.00591400
C	-2.22371000	-1.50685900	-0.00330400
C	-0.81235200	-3.89144700	-0.00762700
H	0.91935200	-2.56968700	-0.00631800
C	-2.92255600	-2.73309500	-0.00496000
C	-2.22356500	-3.94194200	-0.00704800
H	-0.20847400	-4.80497700	-0.00932100
H	-4.01738100	-2.73098400	-0.00455300
H	-2.75759500	-4.89802600	-0.00824100
C	-2.85008800	-0.19288100	-0.00127000
C	-4.24143200	0.04033400	-0.00091100
C	-2.44006700	2.11939100	0.00171600
C	-4.73807000	1.34565000	0.00074100
H	-4.93204200	-0.80956800	-0.00196800
C	-3.80157900	2.40571000	0.00203000
H	-1.69166000	2.91690700	0.00277200
H	-5.81580200	1.53803800	0.00099500
H	-4.12838100	3.45150300	0.00328600
N	-1.92734400	0.84709900	0.00013900
N	-0.83675200	-1.45641100	-0.00354500
N	0.58793300	2.11258800	0.00322900
C	0.98416300	3.23410700	0.00488600
C	1.65009500	4.53509300	0.00643100
H	1.36993500	5.13142300	-0.88290900
H	1.37636300	5.12597400	0.90138500
H	2.75193300	4.41721200	0.00210300

[Mn-H]$^{-1}$

	x	y	z
C	2.67083400	-1.19080700	0.17463000
C	1.92273900	-0.00037700	-0.02730000
C	2.67157700	1.18923800	0.17670700
C	4.00511000	1.22144300	0.62593300
C	4.66560300	-0.00201100	0.86548200
C	4.00435000	-1.22463000	0.62377600
H	4.51193200	2.18285200	0.77513400
H	5.70426800	-0.00264400	1.22098700
H	4.51057600	-2.18661600	0.77126700
O	2.02603100	-2.39894900	-0.10449900
O	2.02749600	2.39825000	-0.10027900
P	0.52384000	-2.04186700	-0.90147800
P	0.52521500	2.04339700	-0.89815300
Mn	-0.06970800	0.00069000	-0.52155600
C	-0.42479300	-3.51503300	-0.25357600
H	-1.39060600	-3.58500800	-0.78632200
H	0.14080300	-4.45640400	-0.38846400
H	-0.62897800	-3.36354000	0.81982700
C	0.95997200	-2.68072900	-2.59880100
H	1.40081600	-3.69451800	-2.55447700
H	0.05131200	-2.69331400	-3.22836300
H	1.68199000	-1.97905300	-3.04908600
C	0.96215100	2.68452800	-2.59441800
H	0.05369100	2.69840700	-3.22423600
H	1.40343700	3.69805500	-2.54850400
H	1.68400300	1.98318200	-3.04549300
C	-0.42247300	3.51630400	-0.24823300
H	0.14401600	4.45740300	-0.38126000
H	-1.38793600	3.58801600	-0.78138600
H	-0.62736500	3.36318900	0.82479300
H	0.03359600	0.00197000	-2.11856200
C	0.18745200	-0.00145600	2.48499500
C	-2.00483100	-0.00109400	1.62240500
C	-0.28027500	-0.00261400	3.79167700
H	1.25880100	-0.00114400	2.26586100
C	-2.52885700	-0.00229900	2.93729800
C	-1.68059500	-0.00308500	4.04288300
H	0.44495100	-0.00315300	4.61429000
H	-3.61659500	-0.00259700	3.07770200
H	-2.07932100	-0.00402600	5.06408200
C	-2.79770500	-0.00007000	0.41853700
C	-4.21207200	-0.00019700	0.36603100
C	-2.70605100	0.00230500	-1.93742800
C	-4.88209700	0.00092800	-0.85673000
H	-4.78097000	-0.00123600	1.30357400
C	-4.09082500	0.00224300	-2.04003200

H	-2.06145200	0.00327400	-2.82152300
H	-5.97739900	0.00080900	-0.90199500
H	-4.55407400	0.00323200	-3.03417000
N	-2.01459400	0.00111600	-0.74558200
N	-0.62199600	-0.00072500	1.36770900

[Mn-CO]^0

	x	y	z
C	2.71700900	0.96990300	-0.69170800
C	1.97422500	0.01599300	0.03630800
C	2.70727600	-1.11637700	0.45120400
C	4.06843000	-1.31992900	0.16206700
C	4.74584100	-0.33369000	-0.57840000
C	4.07851300	0.82612100	-1.01341700
H	4.57822700	-2.22306800	0.51386000
H	5.80780500	-0.46806400	-0.81482900
H	4.59597800	1.60493900	-1.58350100
O	2.05928800	2.12800200	-1.12644500
O	2.04002600	-2.10107600	1.19180500
P	0.43024600	2.16094300	-0.62274800
P	0.41563300	-1.67836600	1.49107000
Mn	-0.00999700	0.26951900	0.48270500
C	-0.38228200	2.55423100	-2.24304200
H	-1.44898300	2.78867000	-2.07535900
H	0.10906000	3.41944000	-2.72236600
H	-0.31450400	1.68359300	-2.91576500
C	0.38452800	3.81618300	0.19910900
H	0.82186700	4.58717400	-0.45934800
H	-0.66117500	4.08577900	0.43317600
H	0.95328400	3.77444100	1.14208200
C	0.37115300	-1.86554000	3.32984000
H	-0.67340000	-1.80433600	3.68523400
H	0.80262300	-2.83634200	3.63093400
H	0.94639700	-1.04949100	3.79631800
C	-0.41293000	-3.25033400	0.96203800
H	0.07151700	-4.12231000	1.43585000
H	-1.47811800	-3.22151700	1.25442500
H	-0.35110400	-3.35433800	-0.13358600
C	0.22206600	-1.21144200	-2.19733000
C	-1.95890000	-0.78976600	-1.43758000
C	-0.24897100	-1.85267200	-3.34411200
H	1.29243200	-1.09932100	-1.99831900
C	-2.49493100	-1.42716800	-2.57913100
C	-1.64040300	-1.96551700	-3.54305500
H	0.46720000	-2.25612700	-4.06711200
H	-3.57957900	-1.49862500	-2.70562800
H	-2.04558200	-2.46279500	-4.43089300
C	-2.75152300	-0.19656000	-0.36772300
C	-4.16250500	-0.17845200	-0.33973100
C	-2.67482300	0.93630400	1.68604700
C	-4.83955000	0.41247000	0.72744400
H	-4.72497100	-0.63201900	-1.16206500
C	-4.06303800	0.98157700	1.76214100
H	-2.05618200	1.36962500	2.47580400
H	-5.93355700	0.43214000	0.75934300
H	-4.53230400	1.46054600	2.62801300
N	-1.98318400	0.36185800	0.64647500
N	-0.59739100	-0.68219000	-1.24515300
C	0.33040400	1.09424300	1.98445800
O	0.54585100	1.65935400	3.01207000

L-L = bpy

M = Fe

[Fe-S]$^+$

	x	y	z
C	-2.57698800	0.64835600	-1.18971300
C	-1.86548100	0.40743900	0.00253400
C	-2.57274800	0.65529300	1.19589000
C	-3.89039800	1.13981400	1.22940600
C	-4.54050200	1.37739500	0.00450100
C	-3.89477200	1.13267700	-1.22130800
H	-4.38877600	1.32161800	2.18666200
H	-5.56852200	1.75532400	0.00523200
H	-4.39652000	1.30908200	-2.17781700
O	-1.93279400	0.39645400	-2.41296300
O	-1.92391400	0.41074200	2.41816100
P	-0.36223300	-0.19280400	-2.20911600
P	-0.35836200	-0.19061300	2.21119000
Fe	-0.02055300	-0.32771000	0.00136400
C	0.60291000	0.97534600	-3.26067500
H	1.63991400	0.61076400	-3.37036100
H	0.14027300	1.05005900	-4.26002900
H	0.62429800	1.97724900	-2.80257600
C	-0.40378700	-1.70273400	-3.26655700
H	-0.75780500	-1.44949400	-4.28070800
H	0.60953000	-2.13731300	-3.33314100
H	-1.08390100	-2.44990200	-2.82805900
C	-0.40855600	-1.70190400	3.26639700
H	0.60075500	-2.14676800	3.32578700
H	-0.75383500	-1.44738600	4.28322800
H	-1.09874900	-2.44129000	2.83038700
C	0.61684500	0.96977000	3.26176100
H	0.15511500	1.04931200	4.26117000
H	1.65058800	0.59607700	3.37153600
H	0.64690500	1.97105800	2.80279900
C	0.22484000	2.63931400	-0.00331400
C	2.23134700	1.41620700	-0.00393200
C	0.91591100	3.85449000	-0.00638700
H	-0.86791900	2.59458700	-0.00164300
C	2.97908200	2.61004900	-0.00725000
C	2.32179700	3.84478200	-0.00855500
H	0.35037500	4.79134000	-0.00699900
H	4.07212600	2.57050900	-0.00868300
H	2.89295600	4.77859900	-0.01107900
C	2.81521600	0.06906700	-0.00155400
C	4.19654800	-0.20783800	-0.00184100
C	2.32035400	-2.22182400	0.00461500
C	4.63821300	-1.53527300	0.00115800
H	4.92198400	0.61092700	-0.00428300
C	3.67576100	-2.56129600	0.00457300
H	1.54051300	-2.98815000	0.00726900
H	5.70818000	-1.76559800	0.00099000
H	3.96855800	-3.61588900	0.00718000
N	1.87558300	-0.93668300	0.00147100
N	0.85360000	1.43119800	-0.00230200
N	-0.75902800	-2.04037000	-0.00004400
C	-1.30121700	-3.08580600	-0.00307600
C	-2.01435100	-4.35976100	-0.00829400
H	-1.74994300	-4.95460500	-0.90094400
H	-1.76528000	-4.95337900	0.88954700
H	-3.10509600	-4.18298800	-0.01775700

[Fe-H]^0

	x	y	z
C	-2.69884300	1.18990300	0.01966100
C	-1.95334300	-0.00015000	-0.12856900
C	-2.69836500	-1.19027900	0.02155000
C	-4.06398300	-1.22440700	0.35314200
C	-4.73782300	-0.00019900	0.52672900
C	-4.06447700	1.22400200	0.35120300
H	-4.58187200	-2.18333100	0.46222500
H	-5.80283300	-0.00020900	0.78654000
H	-4.58273500	2.18289200	0.45882100
O	-2.02459000	2.40147800	-0.18283200
O	-2.02358300	-2.40186800	-0.17915800
P	-0.44522000	2.10734600	-0.76095600
P	-0.44522400	-2.10756500	-0.76003300
Fe	-0.00354100	-0.00005300	-0.51912600
C	0.45640300	3.41916300	0.19089400
H	1.49146300	3.50353800	-0.18629500
H	-0.04788900	4.39676900	0.08972400
H	0.49162400	3.13908500	1.25684500
C	-0.55009600	2.88684900	-2.43591900
H	-0.92896700	3.92258100	-2.37317900
H	0.44992500	2.88886900	-2.90654000
H	-1.22760600	2.28042400	-3.05899200
C	-0.55294900	-2.88676700	-2.43496900
H	0.44620600	-2.88840400	-2.90742800
H	-0.93148100	-3.92259500	-2.37178000
H	-1.23172700	-2.28032500	-3.05664800
C	0.45805900	-3.41942600	0.19010300
H	-0.04617300	-4.39711300	0.08942100
H	1.49255400	-3.50346000	-0.18870100
H	0.49485100	-3.13966000	1.25608000
H	-0.26663100	-0.00023700	-2.03307800
C	-0.23935300	0.00086400	2.47250600
C	1.93140200	0.00034800	1.57674600
C	0.25439500	0.00122300	3.77708200
H	-1.31334400	0.00088800	2.26211800
C	2.48703300	0.00071100	2.87355600
C	1.64899900	0.00114600	3.99149000
H	-0.44999200	0.00155500	4.61560300
H	3.57451100	0.00066400	3.00047200
H	2.06660700	0.00142900	5.00382900
C	2.70666600	0.00000700	0.33842100
C	4.11558900	-0.00019200	0.26402100
C	2.55891000	-0.00056700	-2.00795000
C	4.75156900	-0.00057200	-0.97991300
H	4.70831400	-0.00008700	1.18446300
C	3.94647900	-0.00075300	-2.13988400
H	1.89269200	-0.00073800	-2.87483800
H	5.84431400	-0.00075500	-1.04967800
H	4.39088200	-0.00105000	-3.14076500
N	1.91757100	-0.00019200	-0.79946800
N	0.56297100	0.00042400	1.36667100

$[Fe\text{-}CO]^{+}$

	x	y	z
C	2.67992800	-1.19438800	0.12131500
C	1.95556400	0.00006800	-0.05268300
C	2.67886800	1.19680700	0.10924100
C	4.04096100	1.22987600	0.44587100
C	4.71086900	0.00466200	0.61718100
C	4.04204000	-1.22280800	0.45832700
H	4.55799300	2.18696300	0.56353400
H	5.77499200	0.00643600	0.87566200
H	4.55991900	-2.17819600	0.58567700
O	2.00364800	-2.41292700	-0.04074400
O	2.00141200	2.41304400	-0.06483800
P	0.37789600	-2.22105600	-0.44123100
P	0.37663500	2.21584600	-0.46675400
Fe	0.02634100	-0.00319600	-0.52947300
C	-0.45027800	-3.25123900	0.84262700
H	-1.52639300	-3.34185100	0.61096800
H	0.00029800	-4.25887600	0.85648200
H	-0.33394700	-2.79335000	1.83786900
C	0.24042200	-3.27193300	-1.94446600
H	0.64280900	-4.27788600	-1.73364500
H	-0.81803500	-3.36386800	-2.24669000
H	0.81081500	-2.82462800	-2.77427900
C	0.24169600	3.24956000	-1.98216000
H	-0.81620500	3.33717500	-2.28763800
H	0.64256900	4.25822000	-1.78161000
H	0.81440900	2.79376300	-2.80574300
C	-0.45448900	3.26059200	0.80330800
H	-0.00477400	4.26870400	0.80578800
H	-1.53024700	3.34745700	0.56866400
H	-0.33947400	2.81494200	1.80424700
C	0.24187800	0.01473300	2.49923100
C	-1.92735700	0.00897300	1.60094100
C	-0.25165800	0.02200500	3.80860800
H	1.31537200	0.01367300	2.28624100
C	-2.48440300	0.01620100	2.89555000
C	-1.64186100	0.02276700	4.01282800
H	0.45040700	0.02693900	4.64785600
H	-3.56982300	0.01663100	3.02830200
H	-2.06237300	0.02831800	5.02359600
C	-2.71441300	0.00158800	0.35843000
C	-4.12241400	0.00119700	0.31164900
C	-2.60530100	-0.01174800	-1.98330800
C	-4.77754000	-0.00573300	-0.92438600
H	-4.70339200	0.00651200	1.23819600
C	-3.99845200	-0.01230000	-2.09422200
H	-1.97150100	-0.01670500	-2.87382000
H	-5.87086300	-0.00597400	-0.97453200
H	-4.45824300	-0.01788100	-3.08715900
N	-1.95383400	-0.00494000	-0.78782200
N	-0.56822600	0.00813600	1.40934000
C	0.49596600	-0.01315200	-2.19313500
O	0.83083000	-0.02036800	-3.31904400

L-L = bpy
M = Co

$[Co\text{-}S]^{2+}$

	x	y	z
C	-2.53809000	0.66297000	-1.20216000
C	-1.84720000	0.42581100	-0.00013900
C	-2.53926500	0.65983300	1.20182800
C	-3.85056300	1.15673000	1.22738300
C	-4.49111800	1.40501000	-0.00015500
C	-3.84936800	1.15990800	-1.22771000
H	-4.35189900	1.33846200	2.18278500
H	-5.51504300	1.79209000	-0.00015400
H	-4.34979100	1.34406800	-2.18312400

O	-1.89476400	0.39558000	-2.42106000
O	-1.89717700	0.38909800	2.42063500
P	-0.33013900	-0.17211600	-2.26032400
P	-0.33133800	-0.17535500	2.25997500
Co	-0.04027200	-0.31325300	-0.00018800
C	0.65830700	1.01681500	-3.24703400
H	1.69964200	0.66098200	-3.33874000
H	0.21863200	1.09269700	-4.25739300
H	0.65287200	2.01583200	-2.78338100
C	-0.32569800	-1.69345500	-3.28290900
H	-0.68180300	-1.44884000	-4.29920600
H	0.69933500	-2.09843900	-3.35018900
H	-0.98991400	-2.45595300	-2.84835800
C	-0.32476500	-1.69750000	3.28130100
H	0.70121900	-2.09978000	3.35025400
H	-0.68341200	-1.45455400	4.29710900
H	-0.98611700	-2.46148300	2.84499200
C	0.65455400	1.01447500	3.24822300
H	0.21463800	1.08824800	4.25862900
H	1.69665000	0.66078400	3.33957800
H	0.64703300	2.01400800	2.78571400
C	0.22797900	2.62960900	0.00171500
C	2.22286900	1.37133300	0.00136800
C	0.93934500	3.83517000	0.00261000
H	-0.86490200	2.60399300	0.00150100
C	2.98465700	2.55503200	0.00229300
C	2.34300000	3.80006400	0.00291900
H	0.38876000	4.78077700	0.00306000
H	4.07684100	2.50307400	0.00249800
H	2.92914900	4.72467600	0.00362600
C	2.79564700	0.01391200	0.00048900
C	4.17361000	-0.27573800	0.00052300
C	2.27498800	-2.27543100	-0.00146100
C	4.59661700	-1.61157600	-0.00045800
H	4.91226900	0.53058000	0.00128000
C	3.62960700	-2.63026200	-0.00149700
H	1.48734200	-3.03376700	-0.00232000
H	5.66446400	-1.85253100	-0.00044300
H	3.91454300	-3.68668900	-0.00232000
N	1.85881800	-0.98656100	-0.00046100
N	0.84774000	1.42022500	0.00109100
N	-0.85535400	-2.00946300	-0.00141600
C	-1.46260400	-3.01211900	-0.00181400
C	-2.23433200	-4.24805600	-0.00200500
H	-1.56448100	-5.12578100	0.02059400
H	-2.89571400	-4.28178300	0.88238500
H	-2.86277100	-4.30283200	-0.90902000

[Co-H]$^+$

	x	y	z
C	-2.69942600	1.19651900	-0.03320800
C	-1.96886200	0.00022600	-0.16528300
C	-2.69948000	-1.19618100	-0.03454300
C	-4.07109200	-1.22613700	0.26043700
C	-4.74396600	-0.00003400	0.41281500
C	-4.07103600	1.22620700	0.26180500
H	-4.59315600	-2.18277400	0.35834900
H	-5.81504500	-0.00013500	0.64065900
H	-4.59305200	2.18275900	0.36079500
O	-2.01421600	2.40772700	-0.20249000
O	-2.01431800	-2.40722600	-0.20521800
P	-0.42410200	2.16280400	-0.70244300
P	-0.42437400	-2.16176600	-0.70548000
Co	-0.05781500	0.00037300	-0.51692000
C	0.46856000	3.36960200	0.36282600
H	1.52263700	3.44648200	0.04269500
H	-0.00408000	4.36420600	0.28284100
H	0.43494300	3.03975300	1.41396200
C	-0.39657200	2.95286000	-2.36206900
H	-0.74444100	3.99852600	-2.29737900
H	0.63004000	2.93784500	-2.76956700
H	-1.05711900	2.38844600	-3.04016100
C	-0.39768200	-2.94945500	-2.36623700
H	0.62885600	-2.93458600	-2.77392300
H	-0.74626800	-3.99496900	-2.30292700
H	-1.05798100	-2.38362400	-3.04338900
C	0.46861600	-3.37011400	0.35775200
H	-0.00406000	-4.36460300	0.27658100
H	1.52257400	-3.44655500	0.03712700
H	0.43540700	-3.04169900	1.40935500
H	-0.41907600	0.00148000	-1.95420300
C	-0.24336900	-0.00204600	2.49605000
C	1.90800200	-0.00122900	1.55418800
C	0.27827500	-0.00310200	3.79563200
H	-1.32121100	-0.00193400	2.30248100
C	2.49408900	-0.00225900	2.83503200
C	1.67247200	-0.00319500	3.96925000
H	-0.40344900	-0.00382800	4.65157800
H	3.58207600	-0.00232900	2.94723800
H	2.11370400	-0.00399700	4.97117300
C	2.66683500	-0.00023200	0.28888700
C	4.07302200	-0.00014200	0.20087200
C	2.49100600	0.00149100	-2.05516500
C	4.68944400	0.00077800	-1.05589500
H	4.68219300	-0.00080000	1.10915700
C	3.88148400	0.00160300	-2.20620900
H	1.81778200	0.00211200	-2.91673700
H	5.78112000	0.00084500	-1.13682300
H	4.31680900	0.00232700	-3.21007800
N	1.88397200	0.00060000	-0.84002400
N	0.54535300	-0.00110900	1.39386900

[Co-CO]$^{2+}$

	x	y	z
C	-2.66010800	-1.20672900	-0.11271800
C	-1.95287600	0.00011100	0.04560700
C	-2.65927500	1.20808800	-0.10758400
C	-4.02049300	1.23168700	-0.43849800
C	-4.68619600	0.00242800	-0.60127700
C	-4.02133100	-1.22796500	-0.44379400
H	-4.54160900	2.18618700	-0.55800200
H	-5.75149900	0.00332700	-0.85280200
H	-4.54307900	-2.18159600	-0.56742500
O	-1.98005800	-2.41906300	0.05884400
O	-1.97843800	2.41928300	0.06902100
P	-0.35645300	-2.27612600	0.41775800
P	-0.35508600	2.27397400	0.42821900
Co	-0.04294500	-0.00132800	0.48831500
C	0.48138800	-3.23950300	-0.89475800
H	1.56177600	-3.31020100	-0.67848100
H	0.05333300	-4.25800700	-0.90769100
H	0.33106300	-2.77580300	-1.88194500
C	-0.16449600	-3.28586800	1.93176600
H	-0.55040900	-4.30153600	1.73142500

H	0.90148100	-3.35998200	2.21044000
H	-0.73311300	-2.85208900	2.76998900
C	-0.16334300	3.27721900	1.94658800
H	0.90256600	3.35008200	2.22584000
H	-0.54897800	4.29379900	1.75034500
H	-0.73229000	2.84010300	2.78284300
C	0.48404900	3.24244100	-0.87974600
H	0.05679300	4.26131800	-0.88847300
H	1.56438000	3.31124400	-0.66257900
H	0.33386900	2.78309700	-1.86900900
C	-0.22010400	0.00596400	-2.50778700
C	1.93889200	0.00357500	-1.56065000
C	0.30316700	0.00911100	-3.80704600
H	-1.29810400	0.00551100	-2.32072200
C	2.51903400	0.00662900	-2.84354900
C	1.69705700	0.00947500	-3.97863400
H	-0.37982300	0.01123500	-4.66195400
H	3.60649600	0.00681700	-2.95751700
H	2.13922600	0.01188900	-4.98021800
C	2.70350800	0.00034400	-0.29969300
C	4.10912100	0.00011100	-0.22094600
C	2.54230700	-0.00569200	2.04714400
C	4.73363400	-0.00300900	1.03363700
H	4.71509300	0.00238300	-1.13110900
C	3.93496100	-0.00599100	2.18860000
H	1.89100800	-0.00810400	2.92560500
H	5.82590800	-0.00312800	1.10706200
H	4.37579000	-0.00856500	3.19007600
N	1.93079000	-0.00253000	0.83469300
N	0.57420700	0.00321000	-1.40775500
C	-0.66224200	-0.00457000	2.12154600
O	-1.07927900	-0.00668700	3.20367900

L-L = en
M = Mn

[Mn-S]^{0}

	x	y	z
C	0.77193200	-2.34638300	-0.11579100
C	-0.26262100	-1.38127400	-0.08506900
C	-1.56829400	-1.92078600	-0.15316600
C	-1.85341500	-3.29731600	-0.21762300
C	-0.77465000	-4.20131400	-0.22774500
C	0.55256100	-3.73515500	-0.17870600
H	-2.89171900	-3.64492300	-0.25655500
H	-0.96973600	-5.27938000	-0.27577800
H	1.40198300	-4.42716400	-0.19084600
O	2.09474900	-1.89690800	-0.09229000
O	-2.64753200	-1.02865700	-0.16180700
P	2.17463700	-0.17415400	-0.00337400
P	-2.11086400	0.59428100	0.06336400
Mn	0.10297500	0.61461600	-0.10368300
C	3.54549200	0.00394900	-1.27403800
H	4.01276000	1.00312500	-1.19201900
H	4.32078000	-0.76713300	-1.11520400
H	3.15766700	-0.11449500	-2.30252400
C	3.20773100	-0.02004500	1.53436100
H	4.11080100	-0.65073800	1.45972800
H	3.50422000	1.03319300	1.69357500
H	2.60948400	-0.34529100	2.40034400
C	-2.93770900	0.96641500	1.68686500
H	-2.83173200	2.03892700	1.93398600
H	-4.00831000	0.69883300	1.65107800
H	-2.44316100	0.38036900	2.47826300
C	-3.38415900	1.35135100	-1.08619900
H	-4.38849600	0.93754800	-0.88331500
H	-3.41686800	2.44895900	-0.95162600
H	-3.13372600	1.13491300	-2.14023200
N	0.46134100	2.66469000	-0.58063600
N	0.04724500	0.50463400	-2.23806100
H	1.18370900	3.02684100	0.05516300
H	-0.75857000	-0.06358200	-2.53073000
H	0.87024600	-0.00195500	-2.59015600
H	-0.38144800	3.22324100	-0.39595200
C	-0.01062900	1.86649800	-2.83736000
H	-1.06040100	2.20811000	-2.79611000
H	0.30467900	1.88193600	-3.90021000
C	0.86778200	2.80324500	-2.00112400
H	1.92550200	2.49444100	-2.07680300
H	0.78844600	3.84385500	-2.37822400
N	0.21419200	1.01390500	1.65801200
C	0.33116500	1.48070800	2.76323000
C	0.33476700	1.34190600	4.23379400
H	-0.47976200	1.93301900	4.69406500
H	0.20342100	0.28262600	4.54006300
H	1.28235500	1.70855100	4.67123700

[Mn-H]$^{-1}$

	x	y	z
C	0.93509600	2.07870300	0.37283000
C	-0.15839100	1.19528700	0.12717300
C	-1.43854600	1.77619200	0.36766400
C	-1.63672600	3.09685000	0.81937900
C	-0.50754500	3.91166800	1.05436100
C	0.79128300	3.40577300	0.82477100
H	-2.65526400	3.47721000	0.97249000
H	-0.63938300	4.94323200	1.40848300
H	1.68070000	4.02971200	0.98395400
O	2.21469600	1.58950100	0.12991900
O	-2.55422500	0.98111300	0.11273500
P	2.06419100	-0.01623800	-0.61596900
P	-1.99717300	-0.52811500	-0.62864800
Mn	0.09441400	-0.75668900	-0.27679700
C	3.78570100	-0.54251500	0.02773600
H	4.11265100	-1.46372800	-0.49381300
H	4.54705600	0.24765000	-0.12927600
H	3.72848700	-0.75882500	1.11131900
C	2.63724800	0.45446900	-2.34123200
H	3.54334200	1.09072900	-2.31711800
H	2.83827500	-0.46171100	-2.92859900
H	1.80913700	0.99618900	-2.82737600
C	-2.66234300	-0.22095100	-2.35903100
H	-2.62051200	-1.16123100	-2.94117000
H	-3.70067700	0.16385000	-2.34278900
H	-1.99696400	0.51079600	-2.84627000
C	-3.52678700	-1.49060000	-0.00422400
H	-4.46927700	-0.93505500	-0.18222900
H	-3.58826300	-2.46944900	-0.52067400
H	-3.43138700	-1.67551300	1.08210300
H	0.11808100	-0.88316100	-1.88484000
N	0.35425700	-2.87800200	-0.14011700
N	0.12542300	-0.99016800	1.86792600

H	1.04438500	-3.12039200	-0.85672800
H	-0.63749300	-0.41461100	2.24380500
H	0.99688500	-0.59532100	2.24290800
H	-0.52216000	-3.35316900	-0.38149100
C	-0.01653900	-2.41636900	2.22492600
H	-1.08707500	-2.67817400	2.12110100
H	0.29757000	-2.65584300	3.26610200
C	0.80410700	-3.24081600	1.22258500
H	1.87051600	-2.95650600	1.30014500
H	0.71487700	-4.32802900	1.45161200

$[Mn\text{-}CO]^0$

	x	y	z
C	-1.34335400	1.98674300	-0.25059100
C	-0.10192300	1.32151100	-0.14938400
C	1.02980700	2.12952000	-0.39606200
C	0.96081300	3.49691000	-0.72079400
C	-0.30910800	4.09810200	-0.80452400
C	-1.47791800	3.35074800	-0.56990100
H	1.87752700	4.07096800	-0.89398400
H	-0.38871600	5.16286600	-1.05396700
H	-2.47129300	3.80857500	-0.62923200
O	-2.50780600	1.24704900	-0.02579000
O	2.29276900	1.53287800	-0.31276100
P	-2.15973200	-0.41292100	0.26233900
P	2.18583800	-0.07979900	0.29300400
Mn	0.04675600	-0.66445400	0.22899300
C	-3.37853600	-1.09601700	-0.99059500
H	-3.50995800	-2.18532900	-0.84776000
H	-4.36103200	-0.60291600	-0.88007000
H	-3.02282100	-0.91681500	-2.02126500
C	-3.17208300	-0.65665600	1.79612600
H	-4.16783600	-0.19517500	1.67535800
H	-3.29540700	-1.73554400	2.00426100
H	-2.65578400	-0.19682100	2.65401700
C	3.09517600	0.12127300	1.89601700
H	3.30337100	-0.86755700	2.34371800
H	4.04541400	0.65958000	1.73449000
H	2.46669000	0.68911000	2.60085400
C	3.59003600	-0.74660500	-0.75518100
H	4.44751800	-0.04996400	-0.73723000
H	3.91866700	-1.72796400	-0.36587200
H	3.27830000	-0.87537100	-1.80765800
N	0.19863000	-2.80276800	0.07454800
N	0.11039100	-0.91502700	-1.92755200
H	-0.71064200	-3.22319500	0.29945700
H	1.01112900	-0.57211200	-2.28622100
H	-0.59706100	-0.30365400	-2.35504800
H	0.85372400	-3.15031800	0.78373000
C	-0.09514500	-2.33723100	-2.30749700
H	0.25247700	-2.56528800	-3.33509500
H	-1.18015000	-2.54000400	-2.26749000
C	0.63156800	-3.21690100	-1.28667800
H	0.43898000	-4.28947900	-1.49288700
H	1.72143400	-3.05045800	-1.35390200
C	0.01952200	-0.59884600	1.96542300
O	0.00612300	-0.58389400	3.16226300

L-L = en
M = Fe

$[Fe\text{-}S]^+$

	x	y	z
C	1.07279100	-2.06003100	-0.70424600
C	-0.06422100	-1.29515700	-0.37713400
C	-1.28442700	-1.99893000	-0.36121700
C	-1.39357600	-3.36903500	-0.64765800
C	-0.22020900	-4.07575000	-0.96826900
C	1.03000900	-3.43064500	-1.00224000
H	-2.37016800	-3.86269200	-0.62565900
H	-0.28092000	-5.14526100	-1.19615900
H	1.94860300	-3.97482400	-1.24285700
O	2.31740700	-1.39987500	-0.72043800
O	-2.45271300	-1.28156400	-0.05355600
P	2.21695800	0.07676800	0.10831400
P	-2.18798900	0.38810000	-0.11099600
Fe	0.04846200	0.64291100	0.01281100
C	3.62663900	0.93094600	-0.73843300
H	3.90726200	1.84324400	-0.18213100
H	4.49986200	0.25620800	-0.77571400
H	3.37323100	1.21438900	-1.77456700
C	2.98018800	-0.34526400	1.73707900
H	3.98472000	-0.77493500	1.58350200
H	3.06203800	0.55366000	2.37396800
H	2.34773800	-1.08892500	2.24880900
C	-3.33236800	0.92714700	1.23139100
H	-3.49233800	2.01938100	1.18366800
H	-4.30683600	0.42045500	1.12226500
H	-2.90639500	0.67236800	2.21441800
C	-3.19303100	0.82570800	-1.61303300
H	-4.18711500	0.35167700	-1.53579300
H	-3.33039800	1.91915500	-1.69467000
H	-2.71719300	0.45379100	-2.53756000
N	0.18903900	2.74617200	0.16281800
N	0.22110400	1.04037300	-1.98971000
H	0.82523100	3.03844300	0.91578600
H	-0.44313500	0.46245800	-2.52306800
H	1.14424000	0.75669500	-2.34775500
H	-0.72438200	3.15040900	0.40774400
C	-0.00318100	2.49348300	-2.26449600
H	-1.09270600	2.66663600	-2.27149500
H	0.39071500	2.79453800	-3.25334200
C	0.65283600	3.30088500	-1.14431200
H	1.74976400	3.18798300	-1.18469200
H	0.42214700	4.37751700	-1.25361000
N	-0.09717000	0.42024000	1.84469400
C	-0.18650200	0.26258400	3.01038100
C	-0.29267800	0.02515400	4.44738800
H	-0.77964600	0.87705100	4.95517100
H	-0.88909600	-0.88444100	4.64298900
H	0.70824400	-0.11623000	4.89401900

$[Fe\text{-}H]^0$

	x	y	z
C	0.99478600	2.06799600	0.35677600
C	-0.12195900	1.23651300	0.10696400
C	-1.37980800	1.85955400	0.27756300
C	-1.54225300	3.19820100	0.67733600
C	-0.38807700	3.96760800	0.92082500

C	0.89599500	3.41145100	0.75945100
H	-2.54599100	3.62275500	0.78924100
H	-0.49008400	5.01379900	1.23271100
H	1.80107200	4.00390700	0.93312600
O	2.26405500	1.50225200	0.18475800
O	-2.51967700	1.08520200	0.03400300
P	2.09647100	-0.04814900	-0.55500700
P	-2.05879900	-0.46717800	-0.55296800
Fe	0.06227600	-0.68986500	-0.26139100
C	3.68164400	-0.75498100	0.15141500
H	3.93929200	-1.69872100	-0.36469200
H	4.51823200	-0.04364300	0.02653900
H	3.56480900	-0.96565300	1.22956700
C	2.68447800	0.34604700	-2.27069700
H	3.65672300	0.87048200	-2.25533900
H	2.77216700	-0.58562000	-2.85910100
H	1.92375100	0.98266600	-2.75150700
C	-2.75988200	-0.37805400	-2.26961800
H	-2.69450900	-1.37036700	-2.75287400
H	-3.81192100	-0.04069300	-2.25959500
H	-2.14707400	0.32770900	-2.85360600
C	-3.44866800	-1.40828300	0.28354900
H	-4.42225400	-0.91637400	0.10562400
H	-3.50006700	-2.44315900	-0.10483100
H	-3.27615000	-1.45019200	1.37362300
H	0.05071200	-0.61713300	-1.79557500
N	0.26790300	-2.77238900	-0.29493000
N	0.10663000	-1.01541300	1.82489200
H	0.93263900	-3.05026700	-1.02578300
H	-0.63183800	-0.45826200	2.27251900
H	0.99051500	-0.66121100	2.21255100
H	-0.62389100	-3.21898500	-0.54107900
C	-0.05190300	-2.46372300	2.10973000
H	-1.12887400	-2.70217800	2.03846100
H	0.29147900	-2.75154200	3.12427500
C	0.71814900	-3.24808200	1.04246000
H	1.79842300	-3.03194900	1.12394100
H	0.57681200	-4.33948200	1.18326700

$[Fe\text{-}CO]^{+}$

	x	y	z
C	-1.06965000	2.05000100	-0.50746600
C	0.07218100	1.30191400	-0.16625500
C	1.29395900	1.99905800	-0.17239600
C	1.39955900	3.36077500	-0.49540600
C	0.22361600	4.05672900	-0.82750700
C	-1.02680800	3.41160100	-0.84139200
H	2.37525700	3.85593100	-0.48809100
H	0.28226100	5.12043300	-1.08061100
H	-1.94594000	3.94902400	-1.09376000
O	-2.31075800	1.38531500	-0.51737200
O	2.46194800	1.29047400	0.14854000
P	-2.22486400	-0.09990300	0.28512000
P	2.21464400	-0.37497500	0.20413400
Fe	-0.03506400	-0.63953300	0.22026200
C	-3.58019800	-0.97793200	-0.61582500
H	-3.83980900	-1.91410400	-0.09032400
H	-4.47445000	-0.33069600	-0.64439300
H	-3.29928600	-1.21762100	-1.65536700
C	-3.02348000	0.26327800	1.90460100
H	-4.03250600	0.67765900	1.73898000
H	-3.10118000	-0.65490600	2.51367400
H	-2.41858000	0.99852900	2.46005900
C	3.25464300	-0.82495200	1.65360400
H	3.36501000	-1.92226300	1.72290700
H	4.25642700	-0.37462300	1.54467500
H	2.79514500	-0.45486200	2.58380800
C	3.29325200	-0.92937700	-1.20037800
H	4.28575300	-0.45699800	-1.09637300
H	3.42309400	-2.02659900	-1.18644900
H	2.87700500	-0.62709600	-2.17646200
N	-0.16095100	-2.73874700	0.23249600
N	-0.13606300	-0.95532400	-1.84812300
H	-0.80530300	-3.08340300	0.95585200
H	0.54400000	-0.35096300	-2.32947300
H	-1.04855000	-0.65083700	-2.21550200
H	0.75365800	-3.14057400	0.47668900
C	0.09591900	-2.39471200	-2.17841500
H	1.18508000	-2.56900200	-2.16576000
H	-0.27364500	-2.65545000	-3.18748400
C	-0.58693200	-3.24526500	-1.10917300
H	-1.68306300	-3.14000800	-1.17444400
H	-0.34482200	-4.31594100	-1.24446200
C	0.04090600	-0.42957200	1.93055500
O	0.09214900	-0.29521300	3.09743000

L-L = en
M = Co

$[Co\text{-}S]^{2+}$

	x	y	z
C	-0.98538900	2.07684300	-0.42542100
C	0.12740000	1.21879600	-0.45879600
C	1.35606400	1.77099200	-0.85876900
C	1.49371100	3.11501500	-1.23188000
C	0.34994000	3.93392100	-1.18834700
C	-0.90018200	3.42961300	-0.78415400
H	2.47018700	3.50933400	-1.52919600
H	0.43474300	4.98702400	-1.47440200
H	-1.79180800	4.06359300	-0.76068700
O	-2.22436900	1.53466300	-0.03508600
O	2.48383000	0.92090900	-0.85492400
P	-2.24655800	-0.12084500	-0.26463400
P	2.26026000	-0.32117000	0.25142900
Co	-0.02302800	-0.66592000	0.04775600
C	-2.98820800	-0.30163700	-1.94503100
H	-3.09049100	-1.36149000	-2.23966500
H	-3.99571000	0.15155600	-1.93576200
H	-2.39052000	0.24269600	-2.69799900
C	-3.58016400	-0.64232200	0.87580100
H	-4.44961300	0.02604800	0.74161900
H	-3.89424100	-1.67588500	0.64575900
H	-3.24909100	-0.58789400	1.92449600
C	2.85480400	0.35834000	1.84946100
H	2.78318400	-0.40653800	2.64256900
H	3.90932900	0.66638800	1.74122300
H	2.25139200	1.23671500	2.13001600
C	3.59677900	-1.46509900	-0.27480000
H	4.55579400	-0.91637100	-0.26320600
H	3.67346200	-2.31788300	0.42253400
H	3.43380200	-1.84370700	-1.29691900
N	-0.20667700	-2.70768900	0.40472200
N	0.24058600	-1.22612900	-1.83888100

H	-0.97393700	-2.93256100	1.05301400
H	1.01347400	-0.70397600	-2.28199100
H	-0.59301600	-0.96757100	-2.38639700
H	0.64217300	-3.07467800	0.85778300
C	0.46973300	-2.71069800	-1.93826500
H	1.53217500	-2.90249400	-1.71695200
H	0.26746700	-3.07207800	-2.96212100
C	-0.41930500	-3.39933600	-0.90767700
H	-1.48531000	-3.29721000	-1.17522300
H	-0.19262800	-4.47908900	-0.84989200
N	-0.28097600	-0.22702200	1.86345600
C	-0.45907700	0.09327500	2.97737200
C	-0.67946700	0.50549000	4.35709800
H	-1.22987400	-0.27553500	4.91134000
H	0.28601400	0.68257300	4.86397600
H	-1.26677100	1.44122500	4.38020600

$[Co\text{-}H]^{+}$

	x	y	z
C	-1.30116300	1.93939100	-0.22418000
C	-0.07133200	1.26215600	-0.12690100
C	1.08332800	2.02866600	-0.37713700
C	1.04171400	3.38510900	-0.73068400
C	-0.21708700	4.00534200	-0.83068100
C	-1.40298900	3.29425400	-0.57438900
H	1.96895500	3.93669100	-0.91288600
H	-0.27410300	5.06425000	-1.10338200
H	-2.38436500	3.77347100	-0.64125800
O	-2.47509800	1.21507900	0.02773800
O	2.32879600	1.38689600	-0.26289600
P	-2.14967700	-0.36842700	0.50275800
P	2.15139900	-0.09854800	0.51945800
Co	0.02899300	-0.64297200	0.23066500
C	-3.44455600	-1.25670400	-0.47519300
H	-3.51640700	-2.31319000	-0.15929100
H	-4.42502900	-0.77551600	-0.31122000
H	-3.21714900	-1.21766500	-1.55387700
C	-2.83865600	-0.42845000	2.20766400
H	-3.89346400	-0.10342000	2.21098500
H	-2.77523600	-1.45610000	2.60902800
H	-2.24617500	0.23685500	2.85634800
C	2.70424900	0.25635700	2.23806800
H	2.69382200	-0.67194700	2.83712500
H	3.72163600	0.68400700	2.24097900
H	2.00070100	0.97168000	2.69468600
C	3.60793000	-0.97940100	-0.19781400
H	4.50819200	-0.34882600	-0.08980400
H	3.78103200	-1.93127500	0.33536900
H	3.46047900	-1.18884300	-1.27057100
H	-0.00341800	-0.40273900	1.69259300
N	0.15843700	-2.69392800	0.42329100
N	0.07338800	-1.07145400	-1.80507200
H	-0.75106500	-3.09439800	0.68841500
H	0.96802900	-0.76409700	-2.21064400
H	-0.64137700	-0.52905100	-2.30813300
H	0.80152600	-2.97546300	1.17447000
C	-0.11973100	-2.53675800	-2.01137300
H	0.24720700	-2.87769400	-2.99748900
H	-1.20380100	-2.74266400	-1.97133300
C	0.59731300	-3.28054700	-0.88334700
H	0.39635400	-4.36694300	-0.93835800
H	1.68852500	-3.13686400	-0.96340800

$[Co\text{-}CO]^{2+}$

	x	y	z
C	-0.98341900	2.13907600	-0.09093400
C	0.11228600	1.26625600	-0.20578300
C	1.33700700	1.80181000	-0.63941600
C	1.48130500	3.15314900	-0.97719500
C	0.35634800	3.99147400	-0.85452000
C	-0.88510500	3.50024200	-0.41193900
H	2.45081100	3.53942400	-1.30633900
H	0.45141100	5.05215200	-1.10720700
H	-1.76077900	4.15057200	-0.32509300
O	-2.20870900	1.61036900	0.34790300
O	2.44279500	0.92984000	-0.72799500
P	-2.29635500	-0.03836100	0.13000000
P	2.26730400	-0.38247100	0.29084100
Co	-0.04850800	-0.64960000	0.21266400
C	-3.19703300	-0.22028300	-1.46755300
H	-3.33657500	-1.27937900	-1.74736000
H	-4.19459800	0.23993200	-1.34634500
H	-2.68127700	0.32256100	-2.27929800
C	-3.49000900	-0.54332900	1.41705200
H	-4.38513300	0.10047800	1.34035000
H	-3.79972000	-1.59259900	1.26468000
H	-3.05999400	-0.42967300	2.42531800
C	3.00829000	0.12468200	1.88841600
H	2.93480300	-0.69193800	2.62890000
H	4.07318300	0.36892000	1.72802300
H	2.49374300	1.01567200	2.28472900
C	3.47108400	-1.55875500	-0.43458200
H	4.45721600	-1.06002600	-0.47000800
H	3.56108600	-2.45912000	0.19839200
H	3.19918600	-1.85280100	-1.46098000
N	-0.23888200	-2.70286300	0.43058300
N	0.04331400	-1.10177400	-1.76305100
H	-0.91635300	-2.98270300	1.15449100
H	0.81279200	-0.59847600	-2.23127200
H	-0.80935300	-0.75255000	-2.22318600
H	0.65564900	-3.11316200	0.73565900
C	0.16519800	-2.58665700	-1.97988500
H	1.23199000	-2.85563000	-1.91179700
H	-0.18454900	-2.87027400	-2.98826000
C	-0.63424100	-3.29539800	-0.89194100
H	-1.71727200	-3.13104800	-1.02312300
H	-0.45737600	-4.38578500	-0.91541600
C	-0.12518900	-0.24158000	1.91332700
O	-0.18453000	0.03047600	3.03729300

3. Bis-Bipyridine Systems: $[(bpy)_2M(L)]^{n+}$

L = CO
M = Mn

$[Mn\text{-}S]^{+}$

	x	y	z
C	-0.71512700	3.90841400	0.48473600
C	-0.11083200	2.65400500	0.61280600
C	-2.12388500	1.54962700	0.14705900
C	-2.78890100	2.78175900	-0.00089900
C	-2.08203100	3.97748200	0.16719600
H	-0.11730200	4.81251600	0.63503300
H	-3.85495400	2.80477400	-0.24377500
H	-2.58787400	4.94205100	0.05847900
C	2.56256700	0.47041000	1.90744900
C	3.89757400	0.87547700	1.95832300
C	2.58369800	0.43937800	-0.43751400
C	4.60527000	1.07322600	0.75810200
H	4.37108400	1.03057900	2.93278500
C	3.93175100	0.84713900	-0.44639300
H	5.65251400	1.39084300	0.76255800
H	4.45344500	0.98404000	-1.39802900
N	1.88308100	0.25457400	0.74070500
N	-0.78427100	1.48655200	0.44778300
C	1.80185800	0.17051200	-1.65134500
C	2.29151900	0.30416200	-2.96684600
C	-0.27975200	-0.53105400	-2.46529200
C	1.45773900	0.00618600	-4.04990000
H	3.31756700	0.63886500	-3.14328100
C	0.14432400	-0.42411700	-3.79439000
H	-1.29255400	-0.86426500	-2.22209000
H	1.82658400	0.10635300	-5.07588400
H	-0.54474400	-0.67294600	-4.60716500
C	-2.76916200	0.23515400	0.02470500
C	-4.13934400	0.03977800	-0.23391100
C	-2.42899300	-2.08101200	0.14565900
C	-4.65668600	-1.25848400	-0.30312300
H	-4.79986900	0.90025900	-0.37344100
C	-3.77941500	-2.33924100	-0.10589900
H	-1.71652000	-2.89223700	0.31125500
H	-5.72073600	-1.42370900	-0.49922500
H	-4.13288800	-3.37437000	-0.14044900
N	0.51705600	-0.24161000	-1.40853700
N	-1.91066800	-0.82503700	0.20968000
Mn	0.00286700	-0.35357800	0.58846700
H	1.99471200	0.30895100	2.82638000
H	0.94828200	2.56118300	0.86473100
N	0.61002400	-2.17178900	0.59843400
C	1.05286200	-3.26241300	0.62255700
C	1.60347200	-4.61287700	0.68266000
H	2.70779500	-4.57967700	0.69606300
H	1.26102500	-5.12690900	1.59869600
H	1.28465700	-5.20680300	-0.19253200
C	-0.28997900	-0.40311800	2.34051900
O	-0.49245300	-0.44265300	3.50052700

$[Mn\text{-}H]^{0}$

	x	y	z
C	0.29503100	-2.39620000	-2.86944800
C	-0.18772700	-1.81306000	-1.69766000
C	1.92490200	-0.94151000	-1.17065800
C	2.47174100	-1.50782500	-2.33993900
C	1.65688500	-2.24480300	-3.20431200
H	-0.38920100	-2.96148300	-3.51092600
H	3.53260600	-1.36706900	-2.57037600
H	2.06803600	-2.69024500	-4.11620000
C	-2.55999900	-1.72735000	1.08707900
C	-3.92343200	-1.94649700	0.93491800
C	-2.71540700	0.38213400	0.05934100
C	-4.73316200	-0.95895200	0.32399200
H	-4.35110700	-2.88707800	1.29838000
C	-4.10501800	0.20767600	-0.11135900
H	-5.81092800	-1.10243300	0.19777600
H	-4.68922500	1.00070800	-0.58974500
N	-1.91194600	-0.58256500	0.67234600
N	0.59317600	-1.09550400	-0.84094100
C	-1.97736700	1.56085200	-0.35745300
C	-2.53399900	2.69367100	-0.99312500
C	0.14666000	2.55823400	-0.39283200
C	-1.72033800	3.77738200	-1.32626600
H	-3.60383100	2.71774200	-1.22283100
C	-0.34520800	3.70572100	-1.01543400
H	1.20607800	2.46835600	-0.13726000
H	-2.14251100	4.66064300	-1.81754300
H	0.33851100	4.52689100	-1.25401800
C	2.67420600	-0.16130300	-0.18651100
C	4.05632300	0.10849100	-0.25271900
C	2.51257300	1.01717200	1.83998500
C	4.67195600	0.85578000	0.75557200
H	4.64473200	-0.27225400	-1.09356800
C	3.87491200	1.31834800	1.82309500
H	1.85436900	1.34383300	2.64913500
H	5.74508700	1.07016200	0.71609800
H	4.30391000	1.90559000	2.64155400
N	-0.63168000	1.49317300	-0.05771100
N	1.89418500	0.28960500	0.86247000
Mn	-0.02654900	-0.21068500	0.84085700
H	-0.14317200	0.59448100	2.19763300
H	-1.92472700	-2.47737800	1.56340300
H	-1.23670300	-1.91265200	-1.40417400
C	0.23778900	-1.48362600	2.03881800
O	0.41596700	-2.26370800	2.91345000

$[Mn\text{-}CO]^{+}$

	x	y	z
C	-0.14618600	3.10149600	-2.24022200
C	0.28612700	2.23427500	-1.23013600
C	-1.84164900	1.28043600	-1.02055100
C	-2.34005400	2.11955900	-2.03623900
C	-1.48567100	3.03874000	-2.65589500
H	0.55849800	3.81044800	-2.68505400
H	-3.38926200	2.05979800	-2.33768200
H	-1.86151800	3.69903700	-3.44401600
C	2.66172300	1.28898400	1.44411200
C	4.03201200	1.51973900	1.28814900
C	2.65672900	-0.30852700	-0.27186700
C	4.73802400	0.80524700	0.30645800

H	4.52953200	2.24797400	1.93568500
C	4.03665500	-0.11812100	-0.47670900
H	5.81167400	0.95811400	0.15875400
H	4.56276200	-0.69606100	-1.24149200
N	1.96471100	0.39906400	0.68467400
N	-0.52989100	1.33546800	-0.63062800
C	1.84168500	-1.28040600	-1.02053800
C	2.34009900	-2.11942500	-2.03630300
C	-0.28598400	-2.23453400	-1.22993200
C	1.48578600	-3.03872600	-2.65587600
H	3.38925800	-2.05949200	-2.33788700
C	0.14635700	-3.10169800	-2.24005100
H	-1.31917800	-2.25553000	-0.87374000
H	1.86164700	-3.69894800	-3.44405300
H	-0.55825800	-3.81076700	-2.68480800
C	-2.65673300	0.30857800	-0.27188900
C	-4.03665100	0.11818100	-0.47676300
C	-2.66175300	-1.28906100	1.44397700
C	-4.73803500	-0.80525600	0.30631200
H	-4.56274700	0.69616300	-1.24152000
C	-4.03203400	-1.51982000	1.28795600
H	-2.08749000	-1.82473600	2.20326100
H	-5.81168100	-0.95811400	0.15857500
H	-4.52955800	-2.24811600	1.93542100
N	0.52996700	-1.33560800	-0.63049700
N	-1.96472000	-0.39906600	0.68463100
Mn	-0.00003300	-0.00002100	0.85764600
H	2.08741700	1.82464700	2.20336800
H	1.31935000	2.25517500	-0.87401700
C	0.31499800	-1.20855500	2.12758500
O	0.52916900	-1.99576400	2.97253400
C	-0.31515800	1.20873400	2.12735800
O	-0.52939700	1.99612800	2.97211100

L = CO
M = Fe

[Fe-S]$^{2+}$

	x	y	z
C	-0.70158000	3.89571600	0.49685300
C	-0.08549300	2.64252200	0.60207800
C	-2.10715400	1.52977400	0.16268400
C	-2.78015500	2.75865900	0.04090300
C	-2.07305400	3.95644100	0.20799200
H	-0.10731200	4.80212600	0.64544400
H	-3.85085400	2.78148800	-0.17876600
H	-2.58693300	4.91895100	0.12056400
C	2.53826500	0.46449500	1.91559600
C	3.87803400	0.86739700	1.97084000
C	2.55662800	0.45551700	-0.43455800
C	4.57472500	1.07298600	0.77003600
H	4.35583100	1.01377100	2.94417600
C	3.90405700	0.86124400	-0.44179800
H	5.62265100	1.38948800	0.77494400
H	4.42990500	1.00819900	-1.38898700
N	1.87751700	0.26821000	0.74472300
N	-0.76300400	1.48159100	0.43477200
C	1.76416000	0.19162600	-1.64978900
C	2.25140700	0.33080000	-2.96255900
C	-0.32024000	-0.51554700	-2.46051600
C	1.41377000	0.03360500	-4.04596000
H	3.27589200	0.66756300	-3.14214200
C	0.10469800	-0.40169300	-3.79085900
H	-1.33077600	-0.85469200	-2.22045300
H	1.78055100	0.13809300	-5.07211500
H	-0.58419400	-0.65079200	-4.60345000
C	-2.74945800	0.21066900	0.03640100
C	-4.12076900	0.00620000	-0.20198600
C	-2.38448500	-2.11059900	0.12329400
C	-4.62365700	-1.29955800	-0.27668200
H	-4.79471800	0.85896600	-0.31991400
C	-3.73940900	-2.37691100	-0.10920100
H	-1.66211700	-2.91687100	0.26523600
H	-5.68986200	-1.47151200	-0.45533300
H	-4.08679200	-3.41352900	-0.15003700
N	0.48160700	-0.22442200	-1.41078600
N	-1.89144600	-0.84934500	0.18760800
Fe	-0.00204900	-0.34264300	0.55172600
H	1.97061900	0.29744100	2.83406300
H	0.97777900	2.55643200	0.83441300
N	0.67095300	-2.15213800	0.57518600
C	1.15147400	-3.21907700	0.63283400
C	1.73902000	-4.55032200	0.71210200
H	2.83753500	-4.47608800	0.79955300
H	1.35023600	-5.08505200	1.59697700
H	1.49518000	-5.13326400	-0.19361100
C	-0.35787100	-0.40265600	2.28718600
O	-0.62202400	-0.44962400	3.41683800

[Fe-H]$^{+}$

	x	y	z
C	0.37071200	-2.44197100	-2.88398400
C	-0.15948100	-1.84340300	-1.73429100
C	1.92107600	-0.94639300	-1.14507700
C	2.51862800	-1.51913200	-2.28370500
C	1.73663100	-2.27650800	-3.16467600
H	-0.27987900	-3.02661900	-3.54142000
H	3.58510200	-1.37815300	-2.48083500
H	2.18669900	-2.73090000	-4.05309200
C	-2.56194600	-1.72495700	1.09616300
C	-3.93918100	-1.92965500	0.97628400
C	-2.70985400	0.37517500	0.06241600
C	-4.73153300	-0.93440400	0.38016600
H	-4.37611800	-2.85860300	1.35490300
C	-4.10200500	0.22651900	-0.08100200
H	-5.81397700	-1.05947600	0.27804100
H	-4.69049100	1.01958600	-0.55064900
N	-1.93563900	-0.59660600	0.65741000
N	0.58741800	-1.11091600	-0.87630100
C	-1.95083300	1.54848600	-0.38654100
C	-2.51555300	2.67571300	-1.01359700
C	0.18176100	2.50465500	-0.50537200
C	-1.69261000	3.74238700	-1.39069300
H	-3.59161600	2.72000200	-1.20264400
C	-0.31525800	3.65291100	-1.13153000
H	1.24670000	2.40472400	-0.28274700
H	-2.11807400	4.62646400	-1.87609300
H	0.37170900	4.45899300	-1.40622300
C	2.64009500	-0.14280300	-0.13908800
C	4.01778300	0.14377000	-0.17667700
C	2.41513800	1.05291500	1.87564500

C	4.59568000	0.90725700	0.84512100
H	4.63571100	-0.23261600	-0.99673800
C	3.77806400	1.36957700	1.88985400
H	1.73451000	1.37773800	2.66654400
H	5.66681400	1.13247300	0.82880700
H	4.18438300	1.96382000	2.71370500
N	-0.60609600	1.46682500	-0.13293300
N	1.84770800	0.31749400	0.88422400
Fe	-0.03865900	-0.20248400	0.82069600
H	-0.33186700	0.57257100	2.09986700
H	-1.92490800	-2.47951700	1.56289100
H	-1.21716900	-1.95020200	-1.47572000
C	0.27112800	-1.44993700	1.99392800
O	0.48415100	-2.22181100	2.84955600

[Fe-CO]$^{2+}$

	x	y	z
C	-0.14526800	3.06744700	-2.25777100
C	0.30225000	2.20047200	-1.25250500
C	-1.83740700	1.26103400	-1.00405200
C	-2.34533600	2.10288900	-2.01010300
C	-1.49231600	3.01483600	-2.64625500
H	0.55620000	3.77030100	-2.71691800
H	-3.39997500	2.05319700	-2.29330100
H	-1.87749100	3.67828900	-3.42717500
C	2.61253700	1.32870400	1.46652100
C	3.98791000	1.55623300	1.33878500
C	2.64519000	-0.29074800	-0.24367200
C	4.70980700	0.82874500	0.38001400
H	4.47360900	2.28839500	1.99076100
C	4.02881000	-0.10214900	-0.41568300
H	5.78748700	0.97701700	0.25743500
H	4.57559000	-0.68580800	-1.16104100
N	1.94603000	0.43396600	0.69071000
N	-0.51626300	1.31172000	-0.64298000
C	1.83765000	-1.26155700	-1.00360100
C	2.34534000	-2.10283600	-2.01020800
C	-0.30221700	-2.20046900	-1.25250200
C	1.49210200	-3.01419800	-2.64691600
H	3.39994400	-2.05308800	-2.29355700
C	0.14507100	-3.06680300	-2.25842900
H	-1.34093500	-2.21816300	-0.91567100
H	1.87714800	-3.67714500	-3.42833000
H	-0.55656100	-3.76920100	-2.71801900
C	-2.64496400	0.29014800	-0.24416200
C	-4.02853700	0.10125600	-0.41650300
C	-2.61273100	-1.32809900	1.46720100
C	-4.70967100	-0.82905500	0.37972000
H	-4.57516600	0.68420200	-1.16252200
C	-3.98804700	-1.55565700	1.33940200
H	-2.02565700	-1.87302800	2.21022100
H	-5.78728800	-0.97759000	0.25688700
H	-4.47399900	-2.28717200	1.99191500
N	0.51650900	-1.31238400	-0.64233100
N	-1.94600500	-0.43405300	0.69072200
Fe	0.00015300	-0.00004800	0.80897900
H	2.02546000	1.87415600	2.20920400
H	1.34101200	2.21798100	-0.91591400
C	0.40212500	-1.21898500	2.05035700
O	0.69891500	-2.00341700	2.84732000
C	-0.40221100	1.21930200	2.04987600
O	-0.69963500	2.00355400	2.84676200

L = CO
M = Co

[Co-S]$^{3+}$

	x	y	z
C	-0.67533800	3.89802600	0.44081600
C	-0.05159000	2.64639500	0.53343800
C	-2.10042900	1.52734000	0.14414900
C	-2.76959200	2.75965100	0.04148400
C	-2.05483800	3.95712700	0.19001600
H	-0.07511800	4.80407400	0.57143800
H	-3.84622500	2.78412100	-0.15003800
H	-2.57047100	4.92069700	0.11647800
C	2.47778400	0.49614300	1.94575000
C	3.81941300	0.89161200	2.02237200
C	2.55415000	0.45270500	-0.41977200
C	4.54379000	1.07289500	0.83251800
H	4.27565300	1.05055700	3.00486600
C	3.90400700	0.84912600	-0.39582300
H	5.59443100	1.38178400	0.85904100
H	4.45540500	0.97940700	-1.33148700
N	1.85828600	0.29284800	0.75363800
N	-0.74662800	1.49239000	0.38393500
C	1.77726200	0.17479400	-1.63695800
C	2.27149500	0.27862100	-2.94920800
C	-0.32493400	-0.52761200	-2.45419000
C	1.43629800	-0.03021300	-4.03329800
H	3.30252400	0.59757800	-3.12711400
C	0.11912700	-0.44338300	-3.78166600
H	-1.34144200	-0.84986700	-2.22106800
H	1.81233000	0.04831400	-5.05902400
H	-0.56750000	-0.70020400	-4.59448600
C	-2.74057500	0.21017700	0.02850600
C	-4.11290400	-0.01133600	-0.18464200
C	-2.34681400	-2.12400000	0.09915300
C	-4.60162700	-1.32499100	-0.25413700
H	-4.79942700	0.83406400	-0.28720500
C	-3.70527600	-2.39703200	-0.11019200
H	-1.61593200	-2.92540900	0.22056200
H	-5.67018900	-1.50737600	-0.41171800
H	-4.04339900	-3.43742500	-0.15080700
N	0.48262000	-0.22308000	-1.41196000
N	-1.87845300	-0.85179500	0.15552700
Co	0.00227500	-0.31341800	0.49424800
H	1.89049700	0.34861800	2.85551600
H	1.01703700	2.56526000	0.73668300
N	0.69660800	-2.12685800	0.57425800
C	1.19456500	-3.18182000	0.67923400
C	1.79633700	-4.49815700	0.80503000
H	2.89103000	-4.40350100	0.92931400
H	1.38288900	-5.02119900	1.68746000
H	1.59139100	-5.10020000	-0.09946000
C	-0.45677100	-0.39062400	2.28084200
O	-0.79414800	-0.46302700	3.37225300

[Co-H]$^{2+}$

	x	y	z
C	0.41434300	-2.49153800	-2.86941000

C	-0.14681500	-1.86874600	-1.74533400
C	1.92732500	-0.96277700	-1.11844600
C	2.55218600	-1.56095300	-2.22726700
C	1.78731600	-2.33480100	-3.11239800
H	-0.21860500	-3.08803800	-3.53307100
H	3.62361600	-1.43081300	-2.40212100
H	2.26022600	-2.80974800	-3.97798000
C	-2.53450800	-1.74035000	1.12590400
C	-3.91501000	-1.95213500	1.03744000
C	-2.71794400	0.36274000	0.07687400
C	-4.71918200	-0.96027900	0.45482100
H	-4.34077600	-2.87983200	1.43126600
C	-4.11099300	0.20386800	-0.03238400
H	-5.80366500	-1.08978500	0.38076200
H	-4.71808800	0.98751900	-0.49346800
N	-1.94325300	-0.60975300	0.65950100
N	0.58661100	-1.12305100	-0.89124300
C	-1.96544300	1.52905500	-0.40510400
C	-2.53608200	2.64010800	-1.05024500
C	0.17700000	2.47757700	-0.58815200
C	-1.71393700	3.69395100	-1.47054300
H	-3.61488100	2.68515300	-1.22161700
C	-0.33310200	3.60870000	-1.23821600
H	1.24501400	2.38335700	-0.38324500
H	-2.14636600	4.56677500	-1.96998500
H	0.35003500	4.40520100	-1.54758200
C	2.62229800	-0.13187000	-0.11450000
C	3.99892400	0.15925600	-0.12262700
C	2.34821300	1.13821400	1.86790900
C	4.54882100	0.95642600	0.89108600
H	4.63995900	-0.23962600	-0.91347100
C	3.71086400	1.45590500	1.90166900
H	1.65400800	1.49281800	2.63399100
H	5.62057300	1.18008200	0.89384800
H	4.09837900	2.07693900	2.71476500
N	-0.61475800	1.45867500	-0.18011600
N	1.81929200	0.37083400	0.87985100
Co	-0.04949100	-0.16509500	0.78430100
H	-0.41124500	0.59132300	2.00766000
H	-1.88521800	-2.49273900	1.57946900
H	-1.21147500	-1.96833200	-1.51442900
C	0.33638300	-1.41986600	1.97709700
O	0.60944600	-2.17556500	2.80426400

[Co-CO]$^{3+}$

	x	y	z
C	-0.17688400	3.04351100	-2.27233700
C	0.29171600	2.17520900	-1.27614200
C	-1.86244500	1.23034700	-0.99795600
C	-2.37798100	2.07891900	-1.99368000
C	-1.53096900	2.99291400	-2.63888500
H	0.51972300	3.74729900	-2.73929600
H	-3.43747800	2.03146200	-2.26179500
H	-1.92732500	3.66111600	-3.41125900
C	2.55894400	1.37215700	1.49643700
C	3.93630600	1.60680400	1.40020100
C	2.65084000	-0.25765100	-0.22769500
C	4.68681200	0.88332400	0.45804200
H	4.39987900	2.34241200	2.06606900
C	4.03665100	-0.05377000	-0.35949800
H	5.76734400	1.03958400	0.36578300
H	4.60951900	-0.63224200	-1.09034700
N	1.93126000	0.47151000	0.69140300
N	-0.52983300	1.28861300	-0.66450300
C	1.86273700	-1.22990600	-0.99817400
C	2.37848600	-2.07818200	-1.99404400
C	-0.29129300	-2.17496700	-1.27681400
C	1.53164700	-2.99212300	-2.63954800
H	3.43801600	-2.03054200	-2.26200100
C	0.17752300	-3.04297900	-2.27315100
H	-1.33506200	-2.19395400	-0.95821500
H	1.92816100	-3.66011400	-3.41202200
H	-0.51893400	-3.74676500	-2.74034000
C	-2.65079600	0.25792100	-0.22791800
C	-4.03666200	0.05444400	-0.35994100
C	-2.55949200	-1.37253800	1.49561400
C	-4.68713100	-0.88279600	0.45718400
H	-4.60933100	0.63334200	-1.09060400
C	-3.93689400	-1.60680700	1.39916100
H	-1.95420500	-1.91601700	2.22548000
H	-5.76769200	-1.03876700	0.36477600
H	-4.40072400	-2.34253900	2.06471500
N	0.53009900	-1.28837600	-0.66492200
N	-1.93151400	-0.47177300	0.69093900
Co	-0.00002500	-0.00021700	0.74204800
H	1.95348000	1.91520400	2.22646600
H	1.33545900	2.19406600	-0.95747900
C	0.49387300	-1.26185000	2.00238600
O	0.85387800	-2.04022300	2.75863400
C	-0.49423100	1.26068300	2.00303300
O	-0.85447400	2.03853700	2.75970400

L = Cl
M = Mn

[Mn-S]^0

	x	y	z
C	-0.96241600	3.90987400	0.10490100
C	-0.27310500	2.70032900	0.20224500
C	-2.25872100	1.46178900	0.01674800
C	-3.01022400	2.65097400	-0.08034400
C	-2.36575000	3.88941100	-0.03383000
H	-0.40621800	4.85201200	0.14575600
H	-4.09784700	2.59889000	-0.19144600
H	-2.93964300	4.81958300	-0.10426100
C	2.32640200	0.83801100	1.92825400
C	3.65987100	1.21979400	2.09587200
C	2.66754200	0.41977900	-0.36163200
C	4.53404400	1.19409800	0.98921500
H	4.00677800	1.52719700	3.08810700
C	4.02453900	0.78688000	-0.24788500
H	5.58579600	1.48301800	1.09104300
H	4.67427100	0.75062300	-1.12839900
N	1.81488200	0.43776400	0.72823500
N	-0.88669600	1.48660200	0.16800100
C	2.00626000	0.01876300	-1.59872100
C	2.61517500	-0.01796100	-2.86914300
C	-0.05466200	-0.60996600	-2.54603100
C	1.86788400	-0.35870800	-3.99981700
H	3.67671500	0.23233500	-2.96672400
C	0.49661300	-0.65126600	-3.82446900
H	-1.10811800	-0.84334900	-2.37282100

H	2.33192100	-0.38882400	-4.99101500
H	-0.14227200	-0.91493800	-4.67404700
C	-2.80275400	0.11286100	-0.05678900
C	-4.16765800	-0.20149700	-0.20433300
C	-2.24726000	-2.16847100	-0.08255000
C	-4.57740100	-1.53457400	-0.29146100
H	-4.90684300	0.60541900	-0.23876600
C	-3.58270700	-2.53570300	-0.23056600
H	-1.45762100	-2.92030100	-0.01824700
H	-5.63631500	-1.79135600	-0.39798100
H	-3.84176900	-3.59839800	-0.28807500
N	0.66565800	-0.29780000	-1.42479400
N	-1.82345400	-0.87020700	0.00670300
Mn	-0.02002700	-0.26529900	0.38556800
H	1.60587000	0.81625500	2.75367800
H	0.81235200	2.67588000	0.32714600
N	0.65618900	-2.00626900	0.72765900
C	1.12780600	-3.04742700	1.01643600
C	1.69068100	-4.32220200	1.44897900
H	2.78342100	-4.23938300	1.59570800
H	1.23736500	-4.62849100	2.40972100
H	1.50255600	-5.11882200	0.70558500
Cl	-0.61475000	-0.18024900	2.70145500

[Mn-H]$^{-1}$

	x	y	z
C	0.67910500	-2.39055200	-2.86871400
C	0.05516500	-1.72197800	-1.81728400
C	2.10431300	-0.86477700	-1.03693100
C	2.79205700	-1.52964000	-2.07536900
C	2.08823900	-2.30382900	-3.00191300
H	0.07586100	-2.98258000	-3.56688800
H	3.88116200	-1.43222600	-2.15227400
H	2.61402500	-2.82760100	-3.80878800
C	-2.35760200	-1.89439500	1.08112100
C	-3.70521800	-2.23925900	1.07196000
C	-2.82958400	0.20517100	0.11200900
C	-4.66934000	-1.32642800	0.57076700
H	-4.00254300	-3.21989700	1.46270800
C	-4.20795600	-0.09807600	0.09108000
H	-5.73792700	-1.57130900	0.56054900
H	-4.91323700	0.64409300	-0.30155600
N	-1.87907000	-0.68722900	0.62206200
N	0.72708400	-0.97397100	-0.89550800
C	-2.22801500	1.42732800	-0.36660700
C	-2.90565000	2.50290300	-0.97708500
C	-0.16754600	2.54374300	-0.66512500
C	-2.20485600	3.62124300	-1.43718700
H	-3.99410600	2.44603900	-1.09649400
C	-0.79379800	3.62168600	-1.27834400
H	0.91614200	2.52014600	-0.52620700
H	-2.72625400	4.45945800	-1.91305800
H	-0.18213200	4.46039300	-1.63174300
C	2.70001600	0.02628900	-0.05742800
C	4.07518000	0.32433800	0.04346800
C	2.22921900	1.50675600	1.71497100
C	4.53394800	1.23900000	0.99569200
H	4.78339500	-0.17835000	-0.62556900
C	3.57432500	1.84408900	1.84536200
H	1.45920900	1.92198000	2.37054600
H	5.60175000	1.46912400	1.08716800
H	3.87184100	2.56168600	2.61893200
N	-0.83863800	1.44216400	-0.18073500
N	1.75473700	0.61134300	0.78950000
Mn	-0.05182100	-0.05790500	0.67274400
H	-0.29928000	0.62741800	2.08138400
H	-1.58151600	-2.55481600	1.48228900
H	-1.02758100	-1.78280200	-1.66413800
Cl	0.68446500	-1.93064400	2.04650900

[Mn-CO]^{0}

	x	y	z
C	-0.34349700	3.01388700	-2.37083200
C	0.16142100	2.12029600	-1.42147900
C	-1.95464800	1.19156300	-1.03441100
C	-2.52978200	2.06641300	-1.98225900
C	-1.72209400	2.98457000	-2.65732700
H	0.33007400	3.72011700	-2.86634200
H	-3.60486500	2.03008500	-2.18163900
H	-2.15846700	3.67099100	-3.39108500
C	2.49993800	1.50380800	1.35642000
C	3.86331800	1.80452700	1.27891300
C	2.72890200	-0.23520000	-0.20602600
C	4.68940000	1.05125000	0.42373800
H	4.26673300	2.61882700	1.88948200
C	4.10995900	0.02039700	-0.32355100
H	5.76179700	1.25988000	0.34487200
H	4.72498700	-0.58828500	-0.99358400
N	1.92505400	0.50369800	0.63420500
N	-0.60950400	1.22257100	-0.76428100
C	2.00630500	-1.28197800	-0.93108300
C	2.58745500	-2.15761600	-1.86862300
C	-0.09121300	-2.28530900	-1.25462500
C	1.80196600	-3.11861700	-2.51213200
H	3.65583000	-2.08058100	-2.09316500
C	0.43001800	-3.17475000	-2.19403200
H	-1.14774400	-2.30623700	-0.97680000
H	2.24347500	-3.80515200	-3.24170700
H	-0.23530200	-3.90470300	-2.66659700
C	-2.68588400	0.19142000	-0.26269900
C	-4.07358600	-0.02840400	-0.37249400
C	-2.52582200	-1.49744100	1.35983600
C	-4.69857700	-1.00635200	0.40323200
H	-4.66244900	0.58031500	-1.06588400
C	-3.89264700	-1.75245600	1.29066000
H	-1.89004000	-2.05692800	2.04906100
H	-5.77682100	-1.18012000	0.32974200
H	-4.32099800	-2.52587300	1.93663400
N	0.66329800	-1.34760200	-0.61114000
N	-1.88645100	-0.54937300	0.60115100
Mn	-0.01021900	-0.06775500	0.71286000
H	1.80403300	2.04641900	2.00640100
H	1.22226600	2.11926600	-1.15529500
Cl	-0.56723200	1.66091100	2.25098800
C	0.34395000	-1.13041700	2.09329000
O	0.60123300	-1.82778100	3.01012100

L = Cl
M = Fe

[Fe-S]$^{+}$

	x	y	z

C	-0.77269200	3.89730500	0.39750500
C	-0.12836500	2.65538600	0.40579000
C	-2.15340600	1.51761100	0.10383600
C	-2.86035500	2.73336200	0.08884400
C	-2.16695900	3.93947300	0.23959000
H	-0.18341200	4.81074900	0.52151900
H	-3.94682500	2.73542900	-0.03474600
H	-2.70467800	4.89288900	0.23619000
C	2.34480700	0.55705700	1.97725200
C	3.68521400	0.92286900	2.15790500
C	2.63293300	0.41932400	-0.35205200
C	4.52291700	1.03668400	1.03629400
H	4.05751800	1.11177000	3.16945400
C	3.98783400	0.78035300	-0.23280800
H	5.57519800	1.31885500	1.14564800
H	4.61938600	0.85809400	-1.12250300
N	1.82135900	0.31193800	0.75009200
N	-0.79160900	1.48279400	0.26573700
C	1.94659400	0.13264600	-1.62112700
C	2.54292300	0.21775700	-2.89293700
C	-0.09860200	-0.49421200	-2.58551700
C	1.78550600	-0.06410500	-4.03573500
H	3.59333900	0.50750800	-2.98805200
C	0.43720200	-0.42559900	-3.87566100
H	-1.14088000	-0.77596100	-2.41836900
H	2.23712500	-0.00051500	-5.03077400
H	-0.19892900	-0.65328700	-4.73639400
C	-2.76366300	0.19157800	-0.05102900
C	-4.14027300	-0.04494700	-0.21476400
C	-2.31607100	-2.10536000	-0.16388900
C	-4.60550800	-1.35737300	-0.35586200
H	-4.84455800	0.79163400	-0.22014900
C	-3.67183100	-2.40633800	-0.33191800
H	-1.56024200	-2.89190100	-0.12348000
H	-5.67485700	-1.55745300	-0.47637000
H	-3.98375500	-3.45040000	-0.43116100
N	0.62825300	-0.22676700	-1.47082600
N	-1.85435700	-0.83715600	-0.03060000
Fe	-0.01958000	-0.30523700	0.37075500
H	1.63805500	0.44270800	2.80674600
H	0.95227500	2.58299300	0.54241800
N	0.61568100	-2.08014400	0.59532500
C	1.05165700	-3.13948100	0.84487700
C	1.56826100	-4.45558700	1.20354700
H	2.65913200	-4.40802200	1.36804400
H	1.08836000	-4.80472100	2.13513300
H	1.36642000	-5.18844300	0.40268500
Cl	-0.64374200	-0.35240200	2.61138700

[Fe-H]^0

	x	y	z
C	0.64698700	-2.50315700	-2.81419600
C	0.01929700	-1.81226900	-1.77293300
C	2.04536000	-0.87800400	-1.06125600
C	2.74129900	-1.54563200	-2.08841900
C	2.03950500	-2.36982800	-2.97514600
H	0.05452500	-3.13890300	-3.48028300
H	3.82420500	-1.42388300	-2.19030800
H	2.56661200	-2.89991200	-3.77560500
C	-2.35829300	-1.84374600	1.12998200
C	-3.71513900	-2.17605500	1.11121200
C	-2.79412600	0.22502600	0.10224200
C	-4.64880000	-1.26802700	0.57434400
H	-4.03185600	-3.13956300	1.52405300
C	-4.17361800	-0.05524700	0.06498600
H	-5.71900200	-1.50020600	0.55532500
H	-4.86736000	0.67740400	-0.35968800
N	-1.88239100	-0.66128100	0.64272700
N	0.68862400	-1.01556500	-0.90578900
C	-2.16423200	1.43707900	-0.40783100
C	-2.83452700	2.50553700	-1.03239400
C	-0.10033800	2.51966800	-0.70856500
C	-2.11378400	3.60720900	-1.50357100
H	-3.92226500	2.46620100	-1.14890500
C	-0.71345800	3.60174500	-1.33876600
H	0.98126800	2.48887000	-0.56046700
H	-2.62428200	4.44494100	-1.98952200
H	-0.09516800	4.43224100	-1.69531100
C	2.65603200	0.02582900	-0.07856600
C	4.03068000	0.32110100	-0.00405600
C	2.22101300	1.43799600	1.74910200
C	4.50187700	1.20268600	0.97444800
H	4.72820400	-0.14948700	-0.70396500
C	3.57300800	1.77036900	1.86721500
H	1.46313700	1.82735700	2.43277700
H	5.56970300	1.43431700	1.04767100
H	3.88988800	2.45456600	2.66084700
N	-0.79110600	1.44252400	-0.22825900
N	1.74857500	0.58988300	0.79422500
Fe	-0.05637400	-0.04821300	0.68254600
H	-0.38573300	0.65531200	1.99431300
H	-1.59129000	-2.49683100	1.55901700
H	-1.05852100	-1.89689800	-1.60025800
Cl	0.65795800	-1.80497700	2.06686900

[Fe-CO]$^+$

	x	y	z
C	-0.27508300	2.97366100	-2.40746800
C	0.22226900	2.07857200	-1.45220100
C	-1.90212400	1.17342700	-1.07359300
C	-2.46803500	2.04392600	-2.02345200
C	-1.64754200	2.95535900	-2.69864200
H	0.40596900	3.67199900	-2.90275800
H	-3.54142000	2.01703100	-2.22898100
H	-2.07483700	3.64282400	-3.43565700
C	2.43805700	1.52139400	1.37744100
C	3.80317100	1.83185700	1.31659700
C	2.70339800	-0.22409200	-0.17801100
C	4.63931400	1.08124100	0.47560100
H	4.19429700	2.64917600	1.92982900
C	4.08116600	0.04104600	-0.27947300
H	5.71083800	1.29644500	0.41087800
H	4.71377300	-0.56240200	-0.93636600
N	1.89526700	0.51951100	0.64372700
N	-0.56305500	1.19471100	-0.79859100
C	1.99666500	-1.29087100	-0.90666100
C	2.60282600	-2.17106700	-1.82180300
C	-0.08169600	-2.33071200	-1.23169800
C	1.83206700	-3.15501300	-2.45237900
H	3.67126200	-2.08794300	-2.03917900
C	0.46336300	-3.23492300	-2.15022300

H	-1.14047300	-2.36190300	-0.96412800
H	2.29230700	-3.84726000	-3.16445300
H	-0.18150300	-3.98727300	-2.61398900
C	-2.66337900	0.18714600	-0.28998200
C	-4.05356200	-0.00011000	-0.39448900
C	-2.53255300	-1.47558500	1.35819500
C	-4.68994900	-0.95506100	0.40650400
H	-4.63784600	0.60628800	-1.09179900
C	-3.91112100	-1.70670400	1.29958600
H	-1.90352800	-2.03610700	2.05307300
H	-5.77224500	-1.10387100	0.33927200
H	-4.35676500	-2.45960600	1.95631300
N	0.65784000	-1.37595700	-0.61692000
N	-1.90411200	-0.55632200	0.58048800
Fe	-0.00862300	-0.05667300	0.68670100
H	1.73065100	2.06039400	2.01667100
H	1.28177800	2.07166800	-1.18574000
Cl	-0.65705200	1.58826700	2.18624800
C	0.35263700	-1.04316100	2.09781900
O	0.61761600	-1.67150700	3.04320600

L = Cl
M = Co

[Co-S]$^{2+}$

	x	y	z
C	-0.70922900	3.87374700	0.56009000
C	-0.07665600	2.62407500	0.54863100
C	-2.11584000	1.52016900	0.15108900
C	-2.80284400	2.74587500	0.15089100
C	-2.09551500	3.93706300	0.35853100
H	-0.11300200	4.77440900	0.73312900
H	-3.88508200	2.76852200	-0.00231500
H	-2.62074100	4.89737600	0.36813900
C	2.38487900	0.45717500	1.95718100
C	3.72999300	0.82599000	2.10666100
C	2.58848100	0.44079900	-0.39465100
C	4.51965900	1.00565400	0.96105200
H	4.13792800	0.96592200	3.11223100
C	3.94218700	0.80919600	-0.30223300
H	5.57281200	1.29306200	1.04552700
H	4.54299600	0.94002000	-1.20643900
N	1.83051400	0.27813400	0.73609100
N	-0.75968400	1.47521600	0.34538100
C	1.86474200	0.19932700	-1.65563500
C	2.42508900	0.32933900	-2.93920000
C	-0.20322000	-0.42901000	-2.58608200
C	1.63636500	0.06919600	-4.06800000
H	3.46881000	0.63253900	-3.05851900
C	0.29946100	-0.31762500	-3.88915400
H	-1.23659600	-0.73083800	-2.40099200
H	2.06127400	0.16736100	-5.07213800
H	-0.35367800	-0.53191000	-4.74023000
C	-2.74278400	0.20498400	-0.02917300
C	-4.11817900	-0.01871600	-0.21201000
C	-2.31682800	-2.10836300	-0.11129600
C	-4.59213300	-1.33073700	-0.34519100
H	-4.81558900	0.82293200	-0.23695400
C	-3.67603200	-2.39255400	-0.29505800
H	-1.57102100	-2.90201700	-0.05139200
H	-5.66204200	-1.52000700	-0.47853000
H	-4.00036700	-3.43335400	-0.38586400
N	0.55770600	-0.17764900	-1.49722300
N	-1.85985000	-0.84103900	0.00806000
Co	-0.02095800	-0.31329600	0.39095400
H	1.71556900	0.29943600	2.80889100
H	0.99712500	2.53662500	0.71706300
N	0.64812700	-2.10904700	0.52331600
C	1.10743600	-3.16132900	0.74243000
C	1.65811200	-4.47604500	1.03745300
H	2.75543400	-4.41182700	1.14419300
H	1.23050000	-4.85442300	1.98330000
H	1.42175600	-5.18523400	0.22474000
Cl	-0.60239800	-0.42930500	2.56782800

[Co-H]$^{+}$

	x	y	z
C	0.58043600	-2.45348300	-2.90253600
C	-0.03694200	-1.80867000	-1.82145400
C	1.98342200	-0.85551500	-1.12824600
C	2.67015700	-1.46738100	-2.19340400
C	1.95993900	-2.27671600	-3.09035800
H	-0.01130900	-3.08189800	-3.57485000
H	3.74662600	-1.32353600	-2.32093200
H	2.47911800	-2.76538100	-3.92117800
C	-2.42883000	-1.78472000	1.17238400
C	-3.79780700	-2.07752500	1.10859100
C	-2.76004000	0.27039900	0.07860000
C	-4.67084700	-1.15616500	0.51072900
H	-4.16227300	-3.01742100	1.53377200
C	-4.14258900	0.03210000	-0.01038900
H	-5.74587600	-1.35505600	0.45315800
H	-4.80057000	0.76973700	-0.47822400
N	-1.91577800	-0.63704400	0.66658000
N	0.64077300	-1.02865900	-0.95619500
C	-2.07913000	1.47269600	-0.42083700
C	-2.71414500	2.55082900	-1.06336300
C	0.00504400	2.53233100	-0.62375000
C	-1.95475800	3.64559100	-1.49233200
H	-3.79552900	2.53374700	-1.22446500
C	-0.56881800	3.63354100	-1.26878400
H	1.07821300	2.48838500	-0.42598000
H	-2.43649500	4.49283600	-1.99053100
H	0.06846700	4.46470100	-1.58488900
C	2.62122500	-0.00153400	-0.10398500
C	3.99635000	0.29196100	-0.05597700
C	2.25813500	1.24771200	1.86060100
C	4.50070700	1.08682800	0.98098100
H	4.67088600	-0.10897100	-0.81735800
C	3.61663100	1.57141600	1.95744900
H	1.52955400	1.57591300	2.60500000
H	5.57028700	1.31479900	1.03086900
H	3.96636400	2.18218700	2.79482100
N	-0.72488300	1.47104300	-0.20356200
N	1.76736500	0.49302800	0.84775300
Co	-0.05827900	-0.09035400	0.72823000
H	-0.41430400	0.57136000	1.99440500
H	-1.69989600	-2.45154800	1.64307900
H	-1.10846500	-1.92286900	-1.62806200
Cl	0.59044500	-1.80812900	2.04727000

[Co-CO]$^{2+}$

	x	y	z
C	-0.25287800	2.99069000	-2.39020300
C	0.24603100	2.09186900	-1.43801200
C	-1.89379800	1.19071900	-1.06367500
C	-2.45200500	2.06773600	-2.00944600
C	-1.62511900	2.97920700	-2.68000400
H	0.43152100	3.68710100	-2.88351200
H	-3.52517900	2.04619300	-2.21673400
H	-2.04896000	3.67200400	-3.41398600
C	2.44056400	1.52512500	1.34777400
C	3.81134500	1.81271900	1.28236100
C	2.67983800	-0.24946200	-0.19859400
C	4.63261800	1.03759700	0.44972400
H	4.21450200	2.63012300	1.88774200
C	4.06013100	-0.00271200	-0.29763800
H	5.70785000	1.23384000	0.38468600
H	4.68627500	-0.62036100	-0.94726100
N	1.89306900	0.52466900	0.61705700
N	-0.55140400	1.21224400	-0.79300600
C	1.95742000	-1.31054400	-0.91970000
C	2.54962800	-2.21480500	-1.81936000
C	-0.14933900	-2.31485000	-1.25038400
C	1.75955700	-3.18972700	-2.44335400
H	3.62094900	-2.16012000	-2.03072000
C	0.38729200	-3.24025800	-2.15483500
H	-1.21063600	-2.32318000	-0.99345500
H	2.21131500	-3.90051000	-3.14278000
H	-0.26844700	-3.98489300	-2.61571300
C	-2.65508400	0.20546200	-0.28278200
C	-4.04466700	0.01060800	-0.37252000
C	-2.50258200	-1.46584000	1.37518400
C	-4.66666000	-0.94771800	0.43843600
H	-4.63957100	0.61207100	-1.06507000
C	-3.88160700	-1.70046600	1.32530200
H	-1.86804900	-2.02832700	2.06357200
H	-5.74940200	-1.09943000	0.38239000
H	-4.32032600	-2.45584200	1.98399100
N	0.61492300	-1.37442600	-0.64887000
N	-1.90044600	-0.54258500	0.58446200
Co	-0.01401100	-0.02269200	0.65027900
H	1.75150700	2.08617400	1.98597700
H	1.30561900	2.08098300	-1.17665100
Cl	-0.63609800	1.49865000	2.20323100
C	0.40361200	-0.99850900	2.11379200
O	0.70927700	-1.59256700	3.04939600

L = NH_3
M = Mn

$[Mn\text{-}S]^+$

	x	y	z
C	-0.98454800	3.91415800	0.02733600
C	-0.29443600	2.71787500	0.23013200
C	-2.27466500	1.46163600	0.08355600
C	-3.02360400	2.63758300	-0.12273100
C	-2.38193500	3.87922800	-0.14961500
H	-0.43082700	4.85795800	0.00544700
H	-4.10620700	2.57596200	-0.26851900
H	-2.95348100	4.79856400	-0.31109000
C	2.39694600	0.97051500	1.91893400
C	3.73385000	1.36284000	2.00471000
C	2.61755100	0.41301300	-0.34652700
C	4.55131100	1.26262000	0.86225700
H	4.12479300	1.74017600	2.95492200
C	3.97538300	0.78646700	-0.32034800
H	5.60552500	1.55449900	0.89360000
H	4.57944600	0.70307400	-1.22873600
N	1.81673600	0.48623800	0.78143400
N	-0.90476400	1.49753100	0.27079400
C	1.90356700	-0.04917700	-1.53740400
C	2.46377900	-0.14066200	-2.82709500
C	-0.18058500	-0.74976500	-2.36260700
C	1.67169000	-0.54549100	-3.90557700
H	3.51565500	0.11531600	-2.98546800
C	0.31802800	-0.84963100	-3.66251400
H	-1.22200200	-0.98979200	-2.13577600
H	2.09480100	-0.61740800	-4.91238500
H	-0.34878500	-1.16522400	-4.47106400
C	-2.82381800	0.10857400	0.10379800
C	-4.18763800	-0.21068200	-0.04625900
C	-2.28430700	-2.17275900	0.28126600
C	-4.60376900	-1.54538500	-0.03141900
H	-4.92140100	0.59124900	-0.17320400
C	-3.62053700	-2.54297000	0.13599400
H	-1.50063300	-2.92376200	0.40447700
H	-5.66049300	-1.80454900	-0.14715000
H	-3.88642500	-3.60487900	0.15219400
N	0.58109500	-0.36582800	-1.29886500
N	-1.85136400	-0.87130700	0.27112600
Mn	-0.01887500	-0.24483200	0.56124400
H	1.74817900	1.04919300	2.79607900
H	0.79012600	2.70866700	0.36504300
N	0.67798600	-1.97851300	0.90781500
C	1.18965100	-3.02315500	1.11928900
C	1.81230900	-4.32199300	1.35956600
H	2.87171800	-4.20195500	1.65079300
H	1.29087900	-4.86279800	2.17014200
H	1.77401800	-4.94678900	0.44841100
N	-0.46564300	-0.10384800	2.62173300
H	-1.37401400	-0.55800300	2.79761300
H	0.21425100	-0.58635300	3.22558600
H	-0.55690000	0.86072200	2.97126100

$[Mn\text{-}H]^0$

	x	y	z
C	0.81070200	-3.02934500	-2.21229500
C	0.14885900	-2.15048500	-1.36069900
C	2.17668700	-1.13319700	-0.71428500
C	2.89747000	-2.01166800	-1.55260500
C	2.22386300	-2.97268500	-2.30912600
H	0.23052600	-3.75122600	-2.79736000
H	3.98783300	-1.92666900	-1.61557900
H	2.77309500	-3.65481500	-2.96642000
C	-2.30585100	-1.86665800	1.20980100
C	-3.65155600	-2.21502600	1.27987800
C	-2.80831900	0.16606900	0.13524200
C	-4.62853800	-1.32395000	0.77642200
H	-3.93275700	-3.18010500	1.71573900
C	-4.18303300	-0.13593500	0.18843600
H	-5.69554700	-1.56338000	0.82548600
H	-4.90342700	0.57127600	-0.23673000

N	-1.84116900	-0.67621200	0.69699000
N	0.79006200	-1.21211600	-0.59291800
C	-2.22004900	1.32709800	-0.49578800
C	-2.92318800	2.33540400	-1.18831900
C	-0.17133600	2.36889500	-1.02362300
C	-2.23303500	3.37767900	-1.80816000
H	-4.01601100	2.28691200	-1.24314800
C	-0.81889300	3.37685800	-1.72897700
H	0.91781000	2.34332400	-0.94016900
H	-2.76987600	4.16399700	-2.34892600
H	-0.22252900	4.15744200	-2.21363100
C	2.74724300	-0.04784900	0.06466600
C	4.11987200	0.27096300	0.14524500
C	2.22252400	1.78135700	1.46277000
C	4.54664300	1.36960200	0.89625200
H	4.84951100	-0.35117500	-0.38447300
C	3.56478900	2.14264900	1.56143300
H	1.43082500	2.34060400	1.96904100
H	5.60980900	1.62262000	0.96524200
H	3.84010700	3.01925900	2.15772200
N	-0.83136100	1.35156700	-0.38016200
N	1.77816300	0.69694200	0.74376700
Mn	-0.03706900	-0.00157700	0.70884800
H	-0.34850400	0.94478100	1.97101200
H	-1.53896000	-2.56193600	1.56700500
H	-0.94084200	-2.16816700	-1.25897300
N	0.49855800	-1.25802000	2.29638400
H	1.28439100	-0.81136300	2.78777400
H	-0.27190600	-1.32256700	2.97321200
H	0.79426200	-2.21316900	2.04594100

[Mn-CO]$^{+}$

	x	y	z
C	0.22286000	3.64811500	1.13243700
C	-0.23971800	2.49849800	0.48425800
C	1.90604500	1.55007300	0.44211700
C	2.43555500	2.68162000	1.09815600
C	1.59113200	3.74011100	1.44721500
H	-0.47783400	4.44940700	1.38582700
H	3.50169400	2.73191100	1.33660800
H	1.99181300	4.62190500	1.95758600
C	-2.62693800	0.80949000	-1.85886800
C	-3.99582000	1.08584000	-1.81791000
C	-2.67150600	-0.16888400	0.26613200
C	-4.72986700	0.71746000	-0.67683500
H	-4.47338300	1.57657600	-2.67145000
C	-4.05344000	0.08203400	0.36976400
H	-5.80438700	0.91361300	-0.60882100
H	-4.59953800	-0.22746700	1.26528500
N	-1.94870000	0.19942400	-0.84871000
N	0.56801200	1.46346700	0.13819800
C	-1.87536000	-0.85623600	1.29076700
C	-2.38503200	-1.31775800	2.51964900
C	0.24728100	-1.69781200	1.81728000
C	-1.54360000	-1.98734400	3.41423000
H	-3.43617400	-1.15564100	2.77429500
C	-0.20024000	-2.18198500	3.04984500
H	1.28263400	-1.83623500	1.49804600
H	-1.92764900	-2.35319400	4.37159200
H	0.49969400	-2.70533900	3.70839600
C	2.68443100	0.38125400	0.02555200
C	4.07497100	0.25246700	0.21726500
C	2.60696800	-1.73713000	-0.98855800
C	4.74163500	-0.90075300	-0.20388200
H	4.63332100	1.06141200	0.69761400
C	3.97922600	-1.91377000	-0.82039300
H	2.00102800	-2.51343900	-1.46107400
H	5.82071100	-1.00980200	-0.05973200
H	4.44245100	-2.84041100	-1.17364800
N	-0.55863000	-1.04216600	0.94079100
N	1.92648100	-0.61588900	-0.57880500
Mn	0.00783600	-0.28156300	-0.80790700
H	-2.04124600	1.07726400	-2.74192300
H	-1.29681700	2.38478200	0.22619800
C	-0.30482900	-1.79756800	-1.66902200
O	-0.52363100	-2.79949200	-2.25637200
N	0.44345600	0.73421100	-2.60689100
H	0.21420700	1.73860300	-2.58220100
H	1.45949500	0.68012600	-2.76986600
H	0.00627800	0.34128000	-3.45135900

L = NH_3
M = Fe

[Fe-S]$^{2+}$

	x	y	z
C	-0.76917400	-3.91363500	-0.25378800
C	-0.13103800	-2.67696400	-0.40855200
C	-2.15726400	-1.51755500	-0.17303000
C	-2.85517900	-2.72811400	-0.01073000
C	-2.15799400	-3.94235300	-0.05200300
H	-0.17814900	-4.83364300	-0.29062200
H	-3.93676300	-2.72393900	0.14974500
H	-2.68914100	-4.89118400	0.07291600
C	2.49458500	-0.68123000	-1.93790500
C	3.83547700	-1.07941600	-1.98361800
C	2.56296500	-0.41787100	0.38919900
C	4.56303900	-1.14311100	-0.78411600
H	4.29293600	-1.33220300	-2.94483200
C	3.91542700	-0.80814800	0.41155700
H	5.61423800	-1.44818100	-0.77962500
H	4.46161300	-0.84810700	1.35787200
N	1.85372900	-0.35584800	-0.78633600
N	-0.79746900	-1.49434800	-0.37675700
C	1.78335000	-0.05044100	1.58327700
C	2.28305200	-0.07379100	2.89838400
C	-0.30588900	0.68638900	2.35294500
C	1.45143400	0.29545800	3.96325900
H	3.31425000	-0.38100000	3.09269300
C	0.13261000	0.68417400	3.68299700
H	-1.32422400	0.98736400	2.09838000
H	1.82729900	0.27984900	4.99112400
H	-0.55621500	0.98319800	4.47873700
C	-2.77853800	-0.18403100	-0.13902500
C	-4.15331500	0.05638400	0.04188100
C	-2.35891500	2.12266300	-0.27610800
C	-4.63103300	1.37327200	0.06306300
H	-4.84818300	-0.77908600	0.16415800
C	-3.71442900	2.42512400	-0.09984400
H	-1.61374800	2.91153600	-0.39998900
H	-5.69804300	1.57350200	0.20253600
H	-4.03803200	3.47033900	-0.09189000

N	0.49119600	0.32739700	1.31667800
N	-1.88310100	0.84915800	-0.29439500
Fe	-0.01305000	0.29566700	-0.58329600
H	1.90953000	-0.62750800	-2.85969400
H	0.94826500	-2.61802400	-0.56374300
N	0.65963800	2.07216900	-0.80657600
C	1.15207200	3.13149900	-0.93113400
C	1.75482500	4.45150900	-1.08028100
H	2.83135900	4.35772800	-1.30878700
H	1.26862000	5.00828500	-1.90095600
H	1.64329800	5.03082800	-0.14639100
N	-0.43545300	0.23427900	-2.59155700
H	-0.40473700	-0.71123200	-3.00029200
H	-1.38888600	0.58540700	-2.76677400
H	0.18784400	0.83022200	-3.15483600

[Fe-H]$^+$

	x	y	z
C	-0.65130600	-3.00718500	2.27974700
C	-0.02713200	-2.17714300	1.34317100
C	-2.06242300	-1.16374600	0.76936500
C	-2.75219000	-1.97379900	1.69236500
C	-2.04363700	-2.90867200	2.45642900
H	-0.05286000	-3.71575500	2.86080600
H	-3.83412100	-1.87081000	1.81834200
H	-2.56493200	-3.54369700	3.17969000
C	2.46203800	-1.70804000	-1.28809400
C	3.83459100	-1.96815700	-1.28511700
C	2.75650200	0.28683500	-0.09605700
C	4.70432900	-1.04573400	-0.67588000
H	4.20982400	-2.88277100	-1.75453700
C	4.14829500	0.08423200	-0.06661100
H	5.78598500	-1.21105400	-0.66522900
H	4.79346000	0.81156700	0.43471400
N	1.90425300	-0.59186000	-0.73747000
N	-0.70222200	-1.27165000	0.58738700
C	2.05385700	1.39265700	0.55196100
C	2.66659300	2.41034600	1.30646800
C	-0.06728000	2.28464700	1.00740800
C	1.88071100	3.38826300	1.92413000
H	3.75522200	2.43193000	1.41137800
C	0.48401100	3.31162200	1.77690400
H	-1.14711700	2.20372700	0.86875900
H	2.34367000	4.18665300	2.51226300
H	-0.17958600	4.04148700	2.25087500
C	-2.68341200	-0.12498000	-0.06589300
C	-4.05818500	0.17872400	-0.08536200
C	-2.25747600	1.57009100	-1.64474300
C	-4.53343600	1.20745000	-0.90773200
H	-4.75299600	-0.38896100	0.54057100
C	-3.61121500	1.91674700	-1.69879600
H	-1.50010800	2.08965900	-2.23759400
H	-5.60027900	1.45112100	-0.93138000
H	-3.93164400	2.73152000	-2.35535600
N	0.68653600	1.33842600	0.38194800
N	-1.78133500	0.56777200	-0.85023900
Fe	0.05439700	-0.03879300	-0.79194300
H	0.41613900	0.92212900	-1.93643100
H	1.77026700	-2.42241300	-1.74327600
H	1.05414500	-2.22028200	1.17854700
N	-0.39097200	-1.29962000	-2.33450700
H	0.34796400	-1.27491000	-3.04938500
H	-0.55407600	-2.28498800	-2.07966000
H	-1.24650100	-0.97196300	-2.80437400

[Fe-CO]$^{2+}$

	x	y	z
C	0.20276000	3.61772800	1.20504600
C	-0.26544200	2.48565300	0.52496200
C	1.87922200	1.53551900	0.46490500
C	2.41189300	2.64381100	1.14932400
C	1.56682200	3.69693200	1.52488400
H	-0.49567500	4.41522900	1.47560100
H	3.47729500	2.68839600	1.39061700
H	1.96909500	4.56435100	2.05797600
C	-2.61496300	0.87288300	-1.82350800
C	-3.99061800	1.12496900	-1.77083200
C	-2.64296100	-0.18662300	0.27015000
C	-4.71224500	0.69862600	-0.64467000
H	-4.47856800	1.63957600	-2.60388800
C	-4.02793900	0.03728800	0.38295300
H	-5.79046500	0.87206300	-0.57000100
H	-4.57246400	-0.31036400	1.26480800
N	-1.94111200	0.23898300	-0.83118200
N	0.54428200	1.46290900	0.15820300
C	-1.82924200	-0.88986700	1.27650900
C	-2.33118300	-1.40364000	2.48600800
C	0.31822200	-1.68804800	1.78964600
C	-1.47170900	-2.07764200	3.36330600
H	-3.38726800	-1.28389800	2.74176300
C	-0.12283600	-2.22306400	3.00660900
H	1.35861900	-1.79085700	1.47544800
H	-1.85150300	-2.48625600	4.30505600
H	0.58674100	-2.74758800	3.65335700
C	2.67058000	0.37517500	0.01473900
C	4.06055500	0.24720300	0.19461400
C	2.59306000	-1.71046100	-1.06960200
C	4.72282700	-0.89539400	-0.27396200
H	4.62614900	1.03726200	0.69583700
C	3.97300300	-1.89181600	-0.91820500
H	1.98422100	-2.47229100	-1.56260000
H	5.80386900	-1.00313900	-0.13975200
H	4.44073600	-2.80247700	-1.30439200
N	-0.50725900	-1.03143200	0.93960900
N	1.93867800	-0.60624000	-0.61500000
Fe	0.00040200	-0.23546000	-0.80447800
H	-2.03360700	1.18845000	-2.69297100
H	-1.32144400	2.38666400	0.26093700
C	-0.39006000	-1.74017200	-1.64025100
O	-0.67911400	-2.72535400	-2.18822800
N	0.45244400	0.72405100	-2.56688200
H	0.27719000	1.73852100	-2.51504000
H	1.45733000	0.62155900	-2.77399800
H	-0.03550100	0.37733400	-3.40549200

L = NH_3
M = Co

[Co-S]$^{3+}$

	x	y	z

C	-0.71520000	3.90602300	0.27359900
C	-0.08116000	2.66443500	0.41895600
C	-2.13144500	1.51672300	0.18716600
C	-2.81543100	2.73583700	0.03526200
C	-2.10458600	3.94407100	0.07890300
H	-0.11610600	4.82117700	0.31413800
H	-3.89846300	2.74337600	-0.11730400
H	-2.62893700	4.89851700	-0.03769300
C	2.49708300	0.64658100	1.93291400
C	3.84073300	1.04112800	1.96112300
C	2.53213900	0.42252600	-0.41335100
C	4.54732200	1.12540300	0.74993300
H	4.31372400	1.27545700	2.92006500
C	3.88424500	0.81240300	-0.44521000
H	5.59944000	1.42955600	0.73562100
H	4.41928600	0.86906500	-1.39733600
N	1.85187600	0.34945600	0.77716300
N	-0.77013400	1.49649800	0.38235900
C	1.73353500	0.06772700	-1.59674200
C	2.20807500	0.09226000	-2.92047600
C	-0.38037800	-0.67073300	-2.33788300
C	1.35614800	-0.27548900	-3.97215700
H	3.23665800	0.39732200	-3.13249300
C	0.04183900	-0.66580300	-3.67482400
H	-1.39469400	-0.97232800	-2.07215700
H	1.71592100	-0.25831400	-5.00640300
H	-0.65950000	-0.96493100	-4.46006100
C	-2.76192700	0.18905200	0.15726200
C	-4.13731700	-0.05296300	-0.01302800
C	-2.34076400	-2.13070800	0.30663700
C	-4.61348600	-1.37287900	-0.02152300
H	-4.83475300	0.78074900	-0.13541000
C	-3.70055900	-2.42811500	0.14105100
H	-1.59756700	-2.92049400	0.43115800
H	-5.68260500	-1.57269600	-0.15104400
H	-4.02708600	-3.47291500	0.14269900
N	0.44276600	-0.31039300	-1.32482900
N	-1.88044000	-0.85368100	0.31124000
Co	-0.01309700	-0.28513900	0.58075100
H	1.92983500	0.57818800	2.86415700
H	0.99738800	2.59986500	0.56985200
N	0.68871700	-2.08289000	0.77966600
C	1.19857900	-3.13262300	0.88694500
C	1.81685900	-4.44195300	1.02193900
H	2.90324000	-4.33292100	1.19549800
H	1.37448500	-4.98837300	1.87521300
H	1.66183100	-5.03243000	0.10016200
N	-0.42505400	-0.23255300	2.55284100
H	-1.38805800	-0.55729700	2.73104800
H	0.18959200	-0.85571600	3.09813400
H	-0.35994300	0.71141900	2.96382300

[Co-H]$^{2+}$

	x	y	z
C	0.58253500	-2.99618700	-2.33631300
C	-0.03267400	-2.19700400	-1.36190100
C	2.00164200	-1.17207000	-0.80413000
C	2.68306900	-1.94246100	-1.76437800
C	1.96527100	-2.86650500	-2.53808400
H	-0.01564500	-3.70131200	-2.92114800
H	3.76003300	-1.82724500	-1.91476000
H	2.48050200	-3.47368800	-3.28931600
C	-2.54543800	-1.60670900	1.36352100
C	-3.92725000	-1.82450500	1.31837700
C	-2.72944000	0.34009600	0.06552600
C	-4.73518000	-0.90598000	0.62984600
H	-4.35199900	-2.69872700	1.82055400
C	-4.12473200	0.18011600	-0.00959600
H	-5.82096400	-1.03692400	0.58471300
H	-4.73150800	0.90077100	-0.56428700
N	-1.94820000	-0.54451800	0.76757300
N	0.65308200	-1.30892500	-0.60780000
C	-1.97028400	1.41062000	-0.59357600
C	-2.53381000	2.41638200	-1.39810200
C	0.18539300	2.26087800	-0.97742500
C	-1.70176600	3.36618600	-2.00432700
H	-3.61547600	2.45787500	-1.55164000
C	-0.31733500	3.28027300	-1.79544400
H	1.25601900	2.17264000	-0.78762200
H	-2.12780600	4.15659200	-2.63026200
H	0.37558300	3.99296500	-2.25238500
C	2.64284100	-0.15970800	0.06329100
C	4.01807500	0.14080700	0.06147800
C	2.27042900	1.46329700	1.74243100
C	4.51745500	1.12615800	0.92494600
H	4.69727300	-0.39302900	-0.60871900
C	3.62939900	1.79967700	1.77936400
H	1.53627700	1.96173300	2.38097100
H	5.58632700	1.36311600	0.92998400
H	3.97453200	2.57622500	2.46850900
N	-0.61605900	1.34532600	-0.38181400
N	1.78515400	0.50776600	0.90594000
Co	-0.06603500	-0.05581300	0.83773300
H	-0.45630400	0.88956200	1.90584400
H	-1.89846400	-2.31446900	1.88732000
H	-1.10922800	-2.26180200	-1.17368300
N	0.35695300	-1.32400000	2.32275700
H	1.27108700	-1.09571200	2.73948000
H	-0.32455900	-1.26180900	3.09245900
H	0.41158000	-2.30954100	2.02557900

[Co-CO]$^{3+}$

	x	y	z
C	0.21590400	3.57758400	1.29131200
C	-0.26791500	2.45917700	0.59751100
C	1.89325300	1.51072000	0.48498700
C	2.42915100	2.60931300	1.18038700
C	1.58563200	3.65378400	1.58881300
H	-0.48082400	4.36832500	1.58744300
H	3.49901100	2.65302100	1.40407700
H	1.99454900	4.51357900	2.13053300
C	-2.59227800	0.94916600	-1.79547400
C	-3.97257600	1.17933200	-1.74286000
C	-2.62894300	-0.19218100	0.27457700
C	-4.69816300	0.70124400	-0.63877900
H	-4.45896900	1.71862500	-2.56217400
C	-4.01743200	0.01290300	0.37613600
H	-5.78042300	0.85683500	-0.57082600
H	-4.56859600	-0.37060800	1.23951300
N	-1.93144700	0.28858000	-0.80919600
N	0.54897900	1.45195200	0.20090800
C	-1.81137500	-0.91417900	1.26002400

C	-2.30240000	-1.47983700	2.45034200
C	0.36644200	-1.68786900	1.76276500
C	-1.43002700	-2.16389200	3.30985100
H	-3.36234400	-1.39156700	2.70536400
C	-0.07512000	-2.26877800	2.95972000
H	1.41109700	-1.75933300	1.45654500
H	-1.80610100	-2.61200500	4.23592500
H	0.64344600	-2.79739700	3.59424300
C	2.67169700	0.36010500	0.00130800
C	4.06302200	0.20729700	0.14739700
C	2.54432100	-1.71422400	-1.13520800
C	4.69829900	-0.93525400	-0.36294100
H	4.65099600	0.97834700	0.65376300
C	3.92593700	-1.91155400	-1.01399500
H	1.92163300	-2.46093600	-1.63463400
H	5.78151600	-1.05884100	-0.25532400
H	4.37560800	-2.81906400	-1.43028400
N	-0.48000600	-1.02460300	0.93767200
N	1.92804500	-0.60663600	-0.63719400
Co	0.00636200	-0.17298700	-0.77296800
H	-2.01106600	1.30937300	-2.64710800
H	-1.32775300	2.36765100	0.35080000
C	-0.48194200	-1.71556100	-1.64023900
O	-0.84076600	-2.67065200	-2.16200400
N	0.45857100	0.77732400	-2.50942400
H	0.28470800	1.79190000	-2.43315700
H	1.46378000	0.67280400	-2.72008500
H	-0.03783800	0.44505500	-3.35161200

L = Pyrrole
M = Mn

[Mn-S]^0

	x	y	z
C	1.00432100	0.45064000	-3.86435600
C	0.32645000	0.13570900	-2.68636700
C	2.22726700	0.58148400	-1.37779000
C	2.96479700	0.90886100	-2.53601800
C	2.35831500	0.84297800	-3.79211700
H	0.48037700	0.38040900	-4.82300900
H	4.01162800	1.21522300	-2.44417900
H	2.92247800	1.09253900	-4.69708200
C	-2.17795500	-1.84160300	-0.93867600
C	-3.48448400	-2.10012900	-1.36306000
C	-2.70428300	0.41162600	-0.50211200
C	-4.44071000	-1.06476500	-1.34391900
H	-3.74516300	-3.11029300	-1.69521500
C	-4.03730300	0.20273900	-0.90994300
H	-5.47343200	-1.24212500	-1.66307300
H	-4.75124500	1.03256000	-0.88846400
N	-1.77419900	-0.61210600	-0.50079300
N	0.90351600	0.18907700	-1.45331500
C	-2.14524100	1.69962600	-0.09232100
C	-2.84938100	2.92058100	-0.06944500
C	-0.16811200	2.80013200	0.54741500
C	-2.18819800	4.10533400	0.26924400
H	-3.91302000	2.93838700	-0.32834100
C	-0.81132400	4.03753200	0.57377000
H	0.89283800	2.70390100	0.79506800
H	-2.72574700	5.05912200	0.28990600
H	-0.24094600	4.93453400	0.83693500

C	2.73155100	0.62666200	-0.01516500
C	4.05803800	0.93935400	0.34481700
C	2.16299800	0.32538100	2.24436700
C	4.44123300	0.94434000	1.68737100
H	4.78970300	1.16335800	-0.43840300
C	3.45962300	0.62203600	2.65313900
H	1.39023100	0.06249000	2.96923600
H	5.47047300	1.17746900	1.97836600
H	3.70257400	0.59109800	3.72070400
N	-0.80334600	1.63302900	0.23906600
N	1.75833100	0.32991300	0.93472500
Mn	0.00562300	-0.16296200	0.27108600
H	-1.40790000	-2.61775500	-0.92655900
H	-0.71689500	-0.18857500	-2.70236200
N	-0.74107600	-0.37976600	1.99194700
C	-1.29197700	-0.44809500	3.03506000
C	-1.95462500	-0.58553600	4.32810900
H	-3.02395400	-0.83529500	4.19703100
H	-1.48535000	-1.39456000	4.91844500
H	-1.88945500	0.35055200	4.91359400
N	0.52037400	-2.11921600	0.21017200
C	1.19272200	-2.76180400	-0.81640500
C	0.12290900	-3.10863000	1.09032100
C	1.21999100	-4.14717500	-0.59549600
H	1.62665100	-2.20079700	-1.64396700
C	0.53078000	-4.37081500	0.63191100
H	-0.44032100	-2.86242900	1.99055800
H	1.69569400	-4.89394300	-1.23628700
H	0.36057500	-5.32712300	1.13331600

[Mn-H]$^{-1}$

	x	y	z
C	-0.54394900	-0.09224100	3.76632800
C	0.02426500	-0.21199900	2.49992900
C	-1.99653300	0.39350700	1.44836900
C	-2.62691800	0.51721500	2.70593900
C	-1.90992100	0.27153400	3.88098800
H	0.06836300	-0.29370500	4.65300100
H	-3.68071100	0.81501600	2.75411200
H	-2.39175300	0.36247800	4.86130900
C	2.24271200	-2.06560600	0.03090000
C	3.56542600	-2.46290900	0.19813200
C	2.88755800	0.19778600	-0.13888000
C	4.60571500	-1.49874500	0.18955500
H	3.78154600	-3.52977700	0.32766900
C	4.24300600	-0.16043800	0.01933300
H	5.65576000	-1.78902900	0.31066600
H	5.00906400	0.62372100	0.00157300
N	1.86034600	-0.75262000	-0.14790200
N	-0.66371100	0.00910000	1.33875300
C	2.39213200	1.54574600	-0.31040800
C	3.17093200	2.72185700	-0.30616700
C	0.43285800	2.82862200	-0.58979800
C	2.56848900	3.97544400	-0.44702600
H	4.25709500	2.64273200	-0.18076100
C	1.15697000	4.01611200	-0.58193900
H	-0.65472600	2.82673900	-0.70193600
H	3.16759000	4.89314000	-0.44354600
H	0.62176200	4.96771400	-0.68237600
C	-2.59842100	0.68305300	0.16091800
C	-3.94285700	1.05767900	-0.04515800

C	-2.17166500	0.87507300	-2.15070600
C	-4.40887300	1.35264800	-1.32901700
H	-4.62345600	1.10249600	0.81322400
C	-3.48527200	1.25890600	-2.40186600
H	-1.43462500	0.76502500	-2.95021100
H	-5.45405500	1.63497800	-1.49921700
H	-3.79052000	1.46770900	-3.43390700
N	1.00565400	1.58470600	-0.47580400
N	-1.68793700	0.58034600	-0.89776600
Mn	0.07037200	-0.09626300	-0.48942800
H	0.30015800	-0.16167400	-2.06139300
H	1.42384300	-2.78905300	0.02318800
H	1.06815200	-0.51562200	2.36851000
N	-0.58134300	-2.00660900	-0.73876600
C	-1.08392100	-2.84709100	0.23891100
C	-0.46655600	-2.76642300	-1.88413000
C	-1.28383800	-4.13963000	-0.27683200
H	-1.27274600	-2.47814400	1.24865800
C	-0.88371800	-4.08794000	-1.64685600
H	-0.07595300	-2.31258000	-2.79648300
H	-1.68443700	-5.00024600	0.26813700
H	-0.90644800	-4.90275600	-2.37721800

[Mn-CO]^0

	x	y	z
C	0.20648200	0.31270400	-3.79978800
C	-0.24034600	0.06667000	-2.49919300
C	1.87492700	0.51764200	-1.58694700
C	2.39179100	0.77306200	-2.87835400
C	1.55801800	0.66826500	-3.99273800
H	-0.48627500	0.21376800	-4.64128800
H	3.44281500	1.05065100	-3.00220800
H	1.95023600	0.85894800	-4.99761100
C	-2.32023200	-1.97461900	-0.10363000
C	-3.66219000	-2.26881100	-0.37356600
C	-2.79172500	0.32485200	0.01888400
C	-4.60015700	-1.22433700	-0.44749900
H	-3.95825000	-3.31254900	-0.51868900
C	-4.15391800	0.08775600	-0.24809500
H	-5.65679700	-1.42676700	-0.65291000
H	-4.85872200	0.92360100	-0.29430300
N	-1.87707400	-0.70114000	0.09313100
N	0.55973600	0.15650500	-1.40790400
C	-2.20530700	1.65231800	0.24441700
C	-2.91891900	2.86584000	0.19656800
C	-0.22068500	2.80434300	0.73297700
C	-2.25389400	4.07554700	0.42363200
H	-3.99125200	2.86005700	-0.02113500
C	-0.87315400	4.03869700	0.69563600
H	0.84859500	2.72868300	0.94822700
H	-2.79791700	5.02521800	0.38997600
H	-0.30302800	4.95482200	0.88050500
C	2.62470500	0.62448600	-0.34414500
C	3.99461300	0.95448400	-0.27322000
C	2.51715200	0.49056600	1.99910300
C	4.63611400	1.05214600	0.96111700
H	4.55520100	1.12267200	-1.19829200
C	3.86478600	0.81195100	2.12128500
H	1.91284000	0.29250100	2.88649700
H	5.70048800	1.29922300	1.02565500
H	4.30864700	0.86234300	3.12099000
N	-0.85663500	1.62097600	0.51922700
N	1.85924500	0.39135500	0.79697800
Mn	0.02108400	-0.17227100	0.54436000
H	-1.55882800	-2.75546100	-0.02962100
H	-1.27258400	-0.23870400	-2.30559700
C	-0.31907600	-0.31230300	2.27664500
O	-0.58026500	-0.32493200	3.43138800
N	0.57297800	-2.09936500	0.37498300
C	0.85552500	-2.78014700	-0.80017600
C	0.51565800	-3.05604700	1.37365500
C	0.96957400	-4.15579500	-0.55221700
H	0.96514000	-2.25477300	-1.74842200
C	0.75146800	-4.33373600	0.84529900
H	0.31046800	-2.77353000	2.40710100
H	1.20631300	-4.92635500	-1.29020100
H	0.78292100	-5.26964300	1.40842600

L = Pyrrole
M = Fe

[Fe-S]$^+$

	x	y	z
C	-0.61067600	-1.59279700	-3.58515200
C	-0.02011100	-0.96763100	-2.48135000
C	-2.00430500	-1.11523700	-1.24049500
C	-2.65682400	-1.74429500	-2.31740700
C	-1.95828100	-1.98198800	-3.50613400
H	-0.01994100	-1.75804000	-4.49112000
H	-3.70409900	-2.04471000	-2.22422800
H	-2.45460400	-2.46470300	-4.35401700
C	1.97824000	1.91349600	-1.14528100
C	3.25311300	2.24735800	-1.62500900
C	2.74634900	-0.18433100	-0.40369300
C	4.30252600	1.32520400	-1.48960100
H	3.40821600	3.22302700	-2.09548500
C	4.04505500	0.09592800	-0.86636300
H	5.30784600	1.55916300	-1.85497900
H	4.84905100	-0.63393100	-0.73467100
N	1.72132500	0.71572600	-0.56005800
N	-0.69285200	-0.72100700	-1.33046700
C	2.34966900	-1.42484300	0.28705200
C	3.20125300	-2.52170800	0.51853600
C	0.57808400	-2.52985500	1.35600700
C	2.71207600	-3.64798100	1.19176500
H	4.23817800	-2.49730200	0.17114300
C	1.37383900	-3.65084100	1.61935600
H	-0.46934700	-2.48768600	1.66742600
H	3.36298400	-4.50845200	1.37709100
H	0.94501000	-4.50745500	2.14821700
C	-2.60627900	-0.85993300	0.07300700
C	-3.94288000	-1.13735700	0.41310000
C	-2.16898000	-0.10306300	2.24745900
C	-4.39262900	-0.88409600	1.71370500
H	-4.62924000	-1.53697800	-0.33876100
C	-3.48291000	-0.36437800	2.64954300
H	-1.43652500	0.31721200	2.93943700
H	-5.43208900	-1.08565700	1.99100000
H	-3.78479100	-0.14765200	3.67870800
N	1.04308600	-1.43551100	0.70623100
N	-1.72257000	-0.34054900	0.98839400
Fe	-0.00107800	0.18586500	0.24608500

H	1.12309500	2.59090800	-1.22751500
H	1.01812900	-0.62966900	-2.51322900
N	-0.87850100	1.85305600	-0.34689100
C	-1.24258300	2.18679200	-1.64449300
C	-1.16313500	2.95269700	0.43987300
C	-1.74055200	3.49657500	-1.68048700
H	-1.12476400	1.48649400	-2.47111900
C	-1.69401300	3.98588700	-0.34574200
H	-0.98814500	2.93894100	1.51541300
H	-2.10520200	4.02034500	-2.56648000
H	-2.01879300	4.96477600	0.01272200
N	0.62172700	1.01679900	1.83611300
C	1.10379800	1.53566700	2.77195300
C	1.67599800	2.22443200	3.92370500
H	1.24718000	3.23909200	4.00831100
H	1.46461500	1.67172500	4.85604200
H	2.77071500	2.31625200	3.81147500

[Fe-H]^0

	x	y	z
C	-0.49507500	-0.00003400	3.79392900
C	0.06866900	-0.16612400	2.52547800
C	-1.92706600	0.46492000	1.46838400
C	-2.55703000	0.64700300	2.71608200
C	-1.83888500	0.41132900	3.89396000
H	0.10764800	-0.19923300	4.68602700
H	-3.60123100	0.97176000	2.76226800
H	-2.31495300	0.54535900	4.87109500
C	2.19080400	-2.08904800	0.04274400
C	3.51676600	-2.49480300	0.22069600
C	2.85295600	0.16020100	-0.15080500
C	4.54975000	-1.53835500	0.20387400
H	3.72934900	-3.55933000	0.36261100
C	4.20477200	-0.19540600	0.01666300
H	5.59675800	-1.83286500	0.33242200
H	4.97896800	0.57814300	-0.00386500
N	1.84251200	-0.78117400	-0.14549800
N	-0.61797100	0.05471700	1.37566300
C	2.35967200	1.52190800	-0.34494900
C	3.15805700	2.68107100	-0.37991900
C	0.42185800	2.81130300	-0.65025500
C	2.56330200	3.93486700	-0.55505500
H	4.24353800	2.59620200	-0.26748000
C	1.16157600	3.99428400	-0.68628700
H	-0.66582900	2.81794300	-0.75745100
H	3.17345000	4.84354000	-0.58564800
H	0.64149000	4.94880000	-0.81807100
C	-2.55103900	0.70183500	0.16275300
C	-3.89724800	1.06601500	-0.03265700
C	-2.17052600	0.73743500	-2.15722900
C	-4.38272600	1.26988400	-1.32856700
H	-4.56344000	1.17321900	0.82919600
C	-3.49527600	1.10143400	-2.40931600
H	-1.44796500	0.57456900	-2.95998500
H	-5.42979900	1.54385500	-1.49488400
H	-3.82612300	1.23609500	-3.44414400
N	0.98810500	1.58086800	-0.49508200
N	-1.68217300	0.54305600	-0.89937900
Fe	0.06815100	-0.11076700	-0.48673000
H	0.37710300	-0.18971800	-1.98113000
H	1.36191200	-2.80119800	0.04115200
H	1.10260000	-0.50311000	2.39953100
N	-0.60756500	-1.95136900	-0.69979900
C	-1.11928000	-2.77106400	0.29194300
C	-0.48664800	-2.72694800	-1.83501300
C	-1.31942600	-4.06802400	-0.20928700
H	-1.31421800	-2.39154700	1.29563500
C	-0.91293500	-4.03981500	-1.57447900
H	-0.08904800	-2.29547500	-2.75463400
H	-1.72530800	-4.91793200	0.34536100
H	-0.93731300	-4.86504200	-2.29050100

[Fe-CO]$^+$

	x	y	z
C	0.09664200	0.44615200	-3.78286500
C	-0.32471900	0.13263600	-2.48481900
C	1.76045700	0.70236700	-1.58141800
C	2.24938700	1.03344700	-2.86032100
C	1.41089500	0.90260300	-3.97327700
H	-0.59377600	0.32206300	-4.62232300
H	3.27580300	1.38844400	-2.98515700
H	1.77859200	1.15144300	-4.97399100
C	-2.10433900	-2.16973100	-0.11296400
C	-3.41838500	-2.58643200	-0.37637700
C	-2.79189200	0.08061300	0.03038400
C	-4.44322300	-1.63121600	-0.43665700
H	-3.62043500	-3.65150400	-0.52324000
C	-4.12572900	-0.28084900	-0.22890700
H	-5.47782900	-1.92950900	-0.63494600
H	-4.91025900	0.48025400	-0.26004200
N	-1.79669800	-0.86195100	0.07576100
N	0.48036900	0.25522300	-1.40526300
C	-2.32892100	1.45916100	0.28303200
C	-3.15738500	2.59681600	0.26637300
C	-0.46539300	2.78220800	0.81921500
C	-2.60773000	3.85642600	0.53574100
H	-4.22411700	2.49933000	0.04640300
C	-1.23575400	3.95135300	0.81834500
H	0.60533700	2.80286200	1.03999600
H	-3.24188600	4.74871600	0.52861500
H	-0.76248900	4.91260800	1.03956600
C	2.53548700	0.81164000	-0.33628500
C	3.88967000	1.18902600	-0.28017300
C	2.46610000	0.59331800	1.99618300
C	4.54042800	1.26428200	0.95633600
H	4.43633800	1.40792200	-1.20146000
C	3.81004500	0.96306200	2.11606800
H	1.87936500	0.34145100	2.88199100
H	5.59631300	1.54700000	1.01211400
H	4.26897700	0.99933900	3.10848300
N	-0.99009900	1.56294700	0.55658500
N	1.82296700	0.51847900	0.80176900
Fe	0.01235000	-0.17070600	0.51205200
H	-1.26923000	-2.87220300	-0.04726100
H	-1.33046700	-0.24979500	-2.29496100
C	-0.35218000	-0.41808100	2.21024000
O	-0.65780600	-0.51008000	3.33403700
N	0.80480300	-1.96252900	0.31295100
C	1.08278000	-2.61211200	-0.88220100
C	0.95963500	-2.89851100	1.32267400
C	1.41470300	-3.94988200	-0.62856100
H	1.03783600	-2.10114800	-1.84203700

C	1.33712900	-4.13337200	0.77937000
H	0.80911700	-2.63780400	2.37086200
H	1.70007000	-4.69087200	-1.37797400
H	1.54843000	-5.04433900	1.34229600

L = Pyrrole
M = Co

$[Co\text{-}S]^{2+}$

	x	y	z
C	0.41534900	2.14999200	-3.28981000
C	-0.11376100	1.28802200	-2.32096700
C	1.85104300	1.41842800	-1.03339800
C	2.43379600	2.28684300	-1.97314100
C	1.71277400	2.65495500	-3.11585400
H	-0.18766700	2.40766800	-4.16542600
H	3.44652800	2.66689700	-1.81475700
H	2.15784600	3.32481700	-3.85848300
C	-1.92851100	-1.78761400	-1.49972600
C	-3.18931100	-2.06485300	-2.04953900
C	-2.76660300	0.12209200	-0.38854400
C	-4.26382300	-1.21207600	-1.75425500
H	-3.31275300	-2.93922400	-2.69559200
C	-4.05098200	-0.10996200	-0.91171300
H	-5.25950300	-1.40280100	-2.16795600
H	-4.88060600	0.55700900	-0.66102300
N	-1.72655600	-0.71235100	-0.70411200
N	0.58481500	0.92892900	-1.21925600
C	-2.41187300	1.22863300	0.52353500
C	-3.30480200	2.22543000	0.95759800
C	-0.67722800	2.17591100	1.79656200
C	-2.85330300	3.21928100	1.83731300
H	-4.34224600	2.22998300	0.61196500
C	-1.51790200	3.19331300	2.26830100
H	0.37034900	2.11423100	2.10413700
H	-3.53702700	4.00184900	2.18180600
H	-1.12463700	3.94687800	2.95710500
C	2.49060600	0.97427000	0.21113600
C	3.79105500	1.31787600	0.61974900
C	2.16139900	-0.27742200	2.17468500
C	4.27666000	0.84149400	1.84444000
H	4.42248600	1.94377500	-0.01648400
C	3.44489100	0.03737900	2.63869600
H	1.48659100	-0.91025100	2.75315400
H	5.29012300	1.09386700	2.17221400
H	3.78097800	-0.35785000	3.60174200
N	-1.11027700	1.22029200	0.94580900
N	1.69115800	0.18412200	0.99281600
Co	-0.01338800	-0.27776300	0.16962200
H	-1.04800000	-2.41178600	-1.67909200
H	-1.11061100	0.85931100	-2.43447700
N	-0.62416400	-1.41065900	1.60743100
C	-1.09998100	-2.10922300	2.41547000
C	-1.66757800	-3.00045900	3.41753900
H	-2.75042300	-3.12772600	3.24183000
H	-1.18042600	-3.99028400	3.35844200
H	-1.51829500	-2.58621500	4.43020900
N	0.92016400	-1.75770300	-0.66844400
C	1.50709400	-1.73919800	-1.93365300
C	1.51360800	-2.79311100	0.04730300
C	2.38652300	-2.81555400	-2.04586000
H	1.21261800	-1.02161100	-2.69777800
C	2.39478100	-3.48333500	-0.78321700
H	1.23623200	-3.01070500	1.07667300
H	2.92120300	-3.11060100	-2.95042100
H	2.93920000	-4.39272000	-0.52319100

$[Co\text{-}H]^{+}$

	x	y	z
C	-0.44504700	0.23005300	3.82340200
C	0.10995600	-0.02963100	2.56262700
C	-1.87883000	0.55958000	1.47521600
C	-2.50077200	0.84153600	2.70655200
C	-1.77517100	0.67389700	3.89374800
H	0.15524700	0.08037300	4.72578100
H	-3.53805900	1.18598800	2.74187200
H	-2.24398000	0.88528900	4.86028300
C	2.20182100	-2.08595000	0.07127100
C	3.53823200	-2.46163800	0.27491700
C	2.82372400	0.17503600	-0.16586500
C	4.54187700	-1.48263000	0.24895600
H	3.77334700	-3.51685100	0.44230100
C	4.17821200	-0.14701400	0.02687800
H	5.59294000	-1.75112100	0.39663000
H	4.94132000	0.63574100	-0.00005600
N	1.85130800	-0.79202800	-0.14080400
N	-0.58355500	0.13045200	1.41628500
C	2.30773300	1.53167000	-0.41124600
C	3.09909100	2.69233700	-0.49127800
C	0.36337100	2.78581900	-0.79990800
C	2.48853800	3.92882000	-0.73369600
H	4.18394400	2.62916300	-0.36920900
C	1.09374500	3.97678200	-0.88803100
H	-0.72282800	2.77484700	-0.92130700
H	3.09223300	4.83939500	-0.80267400
H	0.57171300	4.91921500	-1.07832100
C	-2.53280100	0.68814900	0.15619900
C	-3.88488500	1.02829400	-0.03353600
C	-2.22177000	0.49530200	-2.16939200
C	-4.40479700	1.09939600	-1.33233700
H	-4.53087400	1.21931300	0.82773600
C	-3.55891400	0.82869200	-2.41874700
H	-1.52472600	0.25783500	-2.97583300
H	-5.45754300	1.35388300	-1.49176700
H	-3.92281500	0.86087500	-3.44982200
N	0.94786700	1.58731200	-0.56987100
N	-1.71468800	0.43612900	-0.91469600
Co	0.06224300	-0.16275000	-0.49072600
H	0.41333200	-0.29165100	-1.91496600
H	1.38529800	-2.81252900	0.07856500
H	1.13784600	-0.39071600	2.45511600
N	-0.60247700	-1.95289500	-0.62135000
C	-1.06355800	-2.73831600	0.42237400
C	-0.46973800	-2.76697300	-1.73269300
C	-1.23036000	-4.05544700	-0.02951500
H	-1.25877400	-2.32395700	1.41040200
C	-0.85325500	-4.07336300	-1.40195900
H	-0.10100800	-2.37045800	-2.67928300
H	-1.60507600	-4.89156200	0.56428800
H	-0.86970700	-4.92803000	-2.08071900

[Co-CO]$^{2+}$

	x	y	z
C	0.13995500	2.60711100	-3.25994900
C	-0.32345800	1.63625600	-2.36045500
C	1.73052500	1.56861000	-1.24344100
C	2.26503100	2.53925600	-2.11223400
C	1.45998700	3.06462700	-3.13284000
H	-0.52479300	2.99057300	-4.03980200
H	3.29531500	2.88759800	-2.00087300
H	1.85981000	3.82068900	-3.81619000
C	-2.26197000	-1.27425100	-1.70492500
C	-3.60287700	-1.36847500	-2.09661200
C	-2.81965000	0.12988100	0.10625700
C	-4.58056400	-0.69681400	-1.34409100
H	-3.86651500	-1.96265600	-2.97672800
C	-4.18190800	0.06454700	-0.23741400
H	-5.63852100	-0.75916900	-1.61842900
H	-4.92598100	0.60625700	0.35285400
N	-1.87785900	-0.55150200	-0.62168300
N	0.44577800	1.12436000	-1.37575100
C	-2.25896300	0.92166600	1.20994200
C	-3.01802200	1.69359600	2.10573900
C	-0.26921200	1.58036300	2.26843300
C	-2.37059700	2.42656200	3.10891400
H	-4.10797600	1.71978800	2.02335200
C	-0.97151800	2.37125700	3.18697800
H	0.81866400	1.50931800	2.30853700
H	-2.94980500	3.02827100	3.81636800
H	-0.41870300	2.92637800	3.95053400
C	2.48098900	0.95204800	-0.12864700
C	3.81656400	1.27145600	0.18446100
C	2.40874500	-0.56230800	1.67473900
C	4.45298600	0.65153300	1.26701100
H	4.35848400	2.00723300	-0.41538000
C	3.73314700	-0.28111300	2.02911000
H	1.81536900	-1.26816400	2.26044000
H	5.49114700	0.89741600	1.51272700
H	4.17892000	-0.78857000	2.88980400
N	-0.89005600	0.86720800	1.30015200
N	1.78839900	0.02971800	0.62004900
Co	-0.05395800	-0.34094300	0.02168900
H	-1.46055500	-1.77322000	-2.25586800
H	-1.34545000	1.25157200	-2.42780100
C	-0.28191700	-1.87080200	1.14166200
O	-0.60102700	-2.09760300	2.27740400
N	0.59804000	-1.95227300	-0.98031400
C	1.77314100	-2.40428000	-1.38587700
C	0.08668600	-2.91706600	0.00928700
C	2.10452500	-3.68285700	-0.78471900
H	2.39999100	-1.82721100	-2.07349300
C	1.11038500	-3.98870600	0.11363200
H	-0.90142200	-3.32908400	-0.28923600
H	2.98513300	-4.28239400	-1.02642600
H	1.01979300	-4.88349600	0.73383700

L = Pyridine
M = Mn

[Mn-S]$^{+}$

	x	y	z
C	-0.22311400	-1.36840000	-3.75409400
C	0.20799400	-0.71089600	-2.59988700
C	-1.62354100	-1.50807100	-1.36640300
C	-2.11227500	-2.18633100	-2.50044700
C	-1.41530900	-2.11656800	-3.71108000
H	0.36874500	-1.29043400	-4.67147500
H	-3.03070300	-2.77651700	-2.42941800
H	-1.78566300	-2.63987800	-4.59820300
C	-1.60199800	2.05496900	-1.36765000
C	-2.55226400	3.04319400	-1.64760600
C	-1.91842300	2.29826900	0.91750500
C	-3.21148600	3.68543400	-0.58698200
H	-2.76713100	3.29821900	-2.69050800
C	-2.87975100	3.29571700	0.72049600
H	-1.64783900	1.97438700	1.92483900
H	-3.96019100	4.46218200	-0.77355100
H	-3.36120100	3.75420800	1.59044200
C	1.68900400	2.37983300	-0.91347700
C	2.86124400	3.00585500	-1.34231400
C	2.83937400	0.39872800	-0.41352500
C	4.07413100	2.29143500	-1.31220700
H	2.81922400	4.04281100	-1.69046100
C	4.05234000	0.97652000	-0.83559500
H	5.01098700	2.75127900	-1.64188200
H	4.97943700	0.39808900	-0.78077200
N	1.64218300	1.09146500	-0.46413800
N	-0.47113300	-0.74977300	-1.41788000
C	2.70838000	-0.95812700	0.12281300
C	3.75967500	-1.89131200	0.22000400
C	1.20769800	-2.50983900	1.04369500
C	3.51646400	-3.16435400	0.74518300
H	4.76355200	-1.62217700	-0.12169600
C	2.20927400	-3.47558600	1.16505500
H	0.18264300	-2.71477900	1.36271100
H	4.32452600	-3.89852300	0.82329600
H	1.96321300	-4.45677900	1.58298600
C	-2.22413000	-1.56263100	-0.03287300
C	-3.41653600	-2.23905600	0.28435700
C	-1.93502400	-0.95817100	2.21638900
C	-3.87491000	-2.27180900	1.60633600
H	-3.98671200	-2.73713400	-0.50572000
C	-3.10600400	-1.62029000	2.59004200
H	-1.31417800	-0.44767400	2.95684500
H	-4.80184100	-2.79291200	1.86458600
H	-3.40911500	-1.62187800	3.64203100
N	1.42933000	-1.26630100	0.53431000
N	-1.26351100	1.66951700	-0.10172600
N	-1.47730500	-0.90198700	0.92966200
Mn	0.05941700	0.13489200	0.27227300
H	0.73763400	2.91594300	-0.91455800
H	-1.07628600	1.54347900	-2.17805100
H	1.12340500	-0.11297400	-2.60046300
N	0.50680800	0.85315600	1.97449700
C	0.90820700	1.28241900	2.99967300
C	1.41890700	1.80860000	4.26225100
H	2.22282700	2.54507800	4.08071300
H	0.61646300	2.30935100	4.83386600
H	1.83062300	0.99461800	4.88658400

[Mn-H]^{0}

	x	y	z
C	-0.19985900	0.62847800	3.81248600
C	0.21972100	0.17728300	2.56428000
C	-1.66138000	1.12068200	1.50890500
C	-2.13711900	1.59521300	2.74974500
C	-1.41230200	1.35189600	3.91903800
H	0.41321600	0.41537500	4.69492300
H	-3.06967200	2.16850200	2.78900400
H	-1.76927900	1.71906500	4.88684100
C	-1.96069900	-2.12949100	0.29962400
C	-2.71539200	-3.30351300	0.20126900
C	-0.61775400	-2.76532500	-1.49121400
C	-2.39927600	-4.25396200	-0.78457600
H	-3.54266200	-3.46272500	0.90187200
C	-1.31889800	-3.96445200	-1.63910300
H	0.21129600	-2.49501800	-2.14979200
H	-2.97352100	-5.18098200	-0.88673100
H	-1.01920200	-4.65887600	-2.43177700
C	1.89485200	-2.29600400	0.49001400
C	3.12527400	-2.89966300	0.72680800
C	2.93089800	-0.28592200	-0.15936200
C	4.32023800	-2.17846800	0.49299600
H	3.14735200	-3.93023200	1.09811600
C	4.20025100	-0.85589300	0.05818700
H	5.30243800	-2.63108100	0.66210700
H	5.09605100	-0.24948200	-0.11382700
N	1.74829000	-1.01071300	0.01435000
N	-0.48054700	0.39199100	1.40617900
C	2.68673100	1.08593000	-0.55927200
C	3.67693200	2.06120400	-0.80276800
C	0.99738300	2.65791700	-1.01552700
C	3.31062400	3.35985800	-1.16133300
H	4.73399500	1.79283700	-0.70599500
C	1.93346000	3.66000000	-1.26103200
H	-0.07459400	2.85524500	-1.09057400
H	4.07170600	4.12361200	-1.35306800
H	1.58681000	4.66414000	-1.52718700
C	-2.29463700	1.34872800	0.21822800
C	-3.53478100	1.99443600	0.02231900
C	-2.04742900	1.07157100	-2.10816100
C	-4.03255100	2.18322000	-1.26848500
H	-4.10505100	2.34149700	0.89055200
C	-3.26134600	1.70927700	-2.35527700
H	-1.42026100	0.68114500	-2.91369900
H	-4.99462600	2.68065000	-1.43070500
H	-3.60250400	1.82852600	-3.38910000
N	1.33632800	1.37667100	-0.68025500
N	-0.91114000	-1.83183500	-0.52846300
N	-1.53979700	0.87301000	-0.85010700
Mn	0.10053500	-0.11051000	-0.41671200
H	0.27824100	-0.31858500	-1.98544900
H	0.96339800	-2.83549600	0.67542200
H	-2.18405900	-1.38677200	1.06975200
H	1.14795300	-0.39045800	2.44844800

[Mn-CO]$^{+}$

	x	y	z
C	-0.37156500	0.70998100	-3.76099400
C	-0.58178200	0.19474800	-2.47810200
C	1.27150400	1.34068300	-1.61543500
C	1.54185800	1.89223800	-2.88554800
C	0.71844600	1.57430700	-3.97007800
H	-1.04936700	0.43610700	-4.57525000
H	2.38622300	2.57374400	-3.02123200
H	0.91834900	1.99737100	-4.95986200
C	1.55470200	-2.22210700	-1.00808400
C	2.50361600	-3.22076700	-1.25469900
C	2.11010800	-2.15094100	1.24320700
C	3.28998800	-3.70005300	-0.19526100
H	2.61717200	-3.61064400	-2.27146800
C	3.07998700	-3.14580300	1.07701800
H	1.92986100	-1.71351400	2.22759800
H	4.04058600	-4.48075900	-0.35581800
H	3.65682400	-3.47713100	1.94634000
C	-1.64694500	-2.64621300	-0.05711600
C	-2.81310600	-3.37835600	-0.29846900
C	-2.84513800	-0.63835000	0.09231100
C	-4.04494300	-2.70375000	-0.35279900
H	-2.74941300	-4.46258600	-0.43239200
C	-4.05358100	-1.31932400	-0.14867600
H	-4.97827400	-3.24520900	-0.53631500
H	-4.99880500	-0.76928000	-0.16305300
N	-1.63577900	-1.29784800	0.12470700
N	0.21696200	0.48423500	-1.41918200
C	-2.74571600	0.80173600	0.37051600
C	-3.83521100	1.69388600	0.37380000
C	-1.28484700	2.53065800	0.98030500
C	-3.62904900	3.04062700	0.69270200
H	-4.83949800	1.33607400	0.12951400
C	-2.32614100	3.46337800	1.00697700
H	-0.25861800	2.81812500	1.22215300
H	-4.46751200	3.74416300	0.70138300
H	-2.11163400	4.50302700	1.27263800
C	2.03394300	1.63514500	-0.39790200
C	3.18975900	2.43878700	-0.36982900
C	2.14860700	1.34575000	1.92665500
C	3.83913500	2.69667300	0.84133500
H	3.58384600	2.85771000	-1.30044100
C	3.29370400	2.13866200	2.01277000
H	1.70572400	0.91195700	2.82607900
H	4.74031400	3.31645200	0.87353800
H	3.74892100	2.31179200	2.99299900
N	-1.47182000	1.22293400	0.66575300
N	1.33539300	-1.67437900	0.22384700
N	1.50383300	1.06845500	0.75065800
Mn	-0.02685700	-0.15415300	0.53162200
H	-0.67613000	-3.14211300	0.00776100
H	0.93223100	-1.83871000	-1.81978500
H	-1.41357900	-0.48723500	-2.27676100
C	-0.19799000	-0.50244000	2.25819000
O	-0.36024700	-0.69552700	3.41307200

L = Pyridine
M = Fe

[Fe-S]$^{2+}$

	x	y	z
C	-0.15984400	-1.26803800	-3.77387500
C	0.25087500	-0.62205800	-2.60181100
C	-1.56863100	-1.48226800	-1.39796500
C	-2.03743800	-2.14994600	-2.54447100
C	-1.33087500	-2.04215800	-3.74868400

H	0.43365500	-1.15883100	-4.68658600
H	-2.94478700	-2.75822100	-2.49528900
H	-1.68433500	-2.55750400	-4.64731500
C	-1.53844400	2.09739600	-1.36324400
C	-2.46989300	3.10377600	-1.64453400
C	-1.94992500	2.25684500	0.91714700
C	-3.16865900	3.70648400	-0.58698200
H	-2.63893500	3.40273500	-2.68377600
C	-2.89685200	3.26854500	0.71815100
H	-1.72196700	1.89830400	1.92274900
H	-3.90411100	4.49540100	-0.77505400
H	-3.41056800	3.70052100	1.58263700
C	1.70993400	2.39374500	-0.81685200
C	2.89885500	3.00036700	-1.24114300
C	2.80506800	0.36243900	-0.39925300
C	4.08115500	2.24378200	-1.25299900
H	2.88839000	4.04920200	-1.55267300
C	4.03025600	0.91256300	-0.81974500
H	5.02788700	2.68371900	-1.58224700
H	4.94204500	0.30939000	-0.79923500
N	1.64386300	1.09726900	-0.42156800
N	-0.43484300	-0.70821400	-1.43315300
C	2.64246900	-1.00642100	0.12008300
C	3.67790100	-1.95584300	0.20377300
C	1.12721300	-2.52340900	1.07012900
C	3.41509800	-3.22292200	0.73965100
H	4.68327000	-1.70994200	-0.14893200
C	2.11549900	-3.50896200	1.18553700
H	0.10466100	-2.71005100	1.40554100
H	4.21109600	-3.97108200	0.80876700
H	1.86036200	-4.48183500	1.61625100
C	-2.19537500	-1.56443900	-0.06945200
C	-3.37315500	-2.27778100	0.21817900
C	-1.96978700	-0.95692800	2.18430400
C	-3.85324500	-2.32251600	1.53356100
H	-3.91428000	-2.79447500	-0.57928800
C	-3.13122700	-1.65542000	2.53618600
H	-1.37688700	-0.43044900	2.93597600
H	-4.76925500	-2.87252500	1.77111200
H	-3.45750000	-1.67076300	3.58050600
N	1.37183900	-1.29753000	0.54692500
N	-1.26341800	1.66633000	-0.09983900
N	-1.50220800	-0.89733300	0.91128200
Fe	0.04494900	0.14241600	0.27355000
H	0.77411100	2.95509300	-0.78450900
H	-0.98248000	1.61620100	-2.17127500
H	1.15098700	-0.00293000	-2.58865000
N	0.50540700	0.85771600	1.98646000
C	0.91409100	1.28748100	3.00013600
C	1.41810500	1.82290900	4.25992700
H	2.25058400	2.52369200	4.07072700
H	0.62009100	2.36503800	4.79764100
H	1.78579400	1.00550100	4.90537800

[Fe-H]$^{+}$

	x	y	z
C	0.07173800	-0.80095900	3.80951900
C	-0.28972900	-0.23623600	2.58183000
C	1.50909300	-1.26737900	1.48877300
C	1.92742700	-1.86697400	2.69264900
C	1.20382700	-1.63385500	3.86862500
H	-0.52678500	-0.59001300	4.70125400
H	2.80627100	-2.51856700	2.70919800
H	1.51421900	-2.09479700	4.81173800
C	1.93973700	2.10763100	0.50881800
C	2.74999400	3.24927500	0.48011700
C	0.86864500	2.73261000	-1.45927400
C	2.60370600	4.16909000	-0.56940600
H	3.48258100	3.40595000	1.27841200
C	1.63837100	3.89774000	-1.55245600
H	0.11244500	2.48140500	-2.20674900
H	3.22366600	5.07019600	-0.62081300
H	1.47761000	4.57660600	-2.39605300
C	-1.84210300	2.37640000	0.45735200
C	-3.07306900	2.99813000	0.68322200
C	-2.88857800	0.38675600	-0.20814800
C	-4.25998400	2.28601700	0.43468700
H	-3.09274000	4.03002100	1.04751200
C	-4.15621800	0.96251300	-0.00752500
H	-5.24005700	2.74727900	0.59080200
H	-5.05869600	0.37349200	-0.19545100
N	-1.72361900	1.09765800	-0.00191400
N	0.40339900	-0.45045500	1.43396800
C	-2.65225700	-0.99700800	-0.62161600
C	-3.65906400	-1.94161400	-0.89431700
C	-0.99019700	-2.59147300	-1.05697600
C	-3.30914200	-3.24597200	-1.25857000
H	-4.71202900	-1.65450800	-0.82171300
C	-1.94448300	-3.57442300	-1.33348800
H	0.07711700	-2.81300200	-1.11855400
H	-4.08178000	-3.98994600	-1.47639800
H	-1.61439400	-4.58131700	-1.60700100
C	2.17605500	-1.43955400	0.18833800
C	3.37853800	-2.14522100	-0.00940700
C	2.05375300	-0.94228200	-2.10727000
C	3.92222700	-2.24647500	-1.29509000
H	3.88890700	-2.60682900	0.84110200
C	3.24332400	-1.63137700	-2.36245000
H	1.48621100	-0.44968000	-2.90039700
H	4.85703500	-2.79128100	-1.46111200
H	3.62738000	-1.68068100	-3.38597000
N	-1.31627300	-1.31646700	-0.71462700
N	1.00213700	1.83102700	-0.44132200
N	1.51307200	-0.83923200	-0.86143100
Fe	-0.08995900	0.15593300	-0.42269600
H	-0.33006100	0.43634100	-1.90504400
H	-0.90455700	2.90502300	0.64121400
H	2.02798500	1.38148000	1.32004900
H	-1.16206800	0.41868000	2.49374900

[Fe-CO]$^{2+}$

	x	y	z
C	-0.32365500	-0.54715800	3.78424800
C	-0.56507200	-0.08390600	2.48467500
C	1.27783900	-1.25804600	1.63479600
C	1.57941800	-1.75399600	2.91742400
C	0.77333000	-1.39472800	4.00534200
H	-0.98605500	-0.24371600	4.60050600
H	2.43144300	-2.42239100	3.06730600
H	0.99595900	-1.77502000	5.00746300
C	1.50886300	2.27612300	0.93877000
C	2.45943100	3.28321300	1.14341800

C	2.13953000	2.04103400	-1.28733400
C	3.28139400	3.67888800	0.07691100
H	2.54515800	3.74508700	2.13199300
C	3.11109000	3.04021000	-1.16074400
H	1.99424800	1.53662200	-2.24528600
H	4.03205900	4.46532900	0.20611600
H	3.71740600	3.30908600	-2.03120600
C	-1.66121400	2.63132500	0.00535100
C	-2.84373500	3.35094900	0.21755600
C	-2.82862700	0.59490100	-0.09334600
C	-4.05970200	2.65362500	0.28464000
H	-2.80187000	4.43970200	0.31643800
C	-4.04852300	1.26190900	0.12179400
H	-5.00371900	3.18391700	0.44558300
H	-4.98697100	0.70164800	0.14615400
N	-1.63946600	1.28165100	-0.12806300
N	0.21586300	-0.41807400	1.42900200
C	-2.70371300	-0.85315400	-0.33816300
C	-3.78067000	-1.75849900	-0.33147700
C	-1.21352000	-2.57791300	-0.90404700
C	-3.55218200	-3.10939200	-0.62403400
H	-4.79255900	-1.41235200	-0.10423500
C	-2.24535300	-3.52474700	-0.92126100
H	-0.18392500	-2.86046700	-1.13382500
H	-4.38254800	-3.82259500	-0.62678200
H	-2.01789300	-4.56612100	-1.16762600
C	2.02737500	-1.60068900	0.41266500
C	3.17273800	-2.41742500	0.39107300
C	2.10670800	-1.37968000	-1.92295500
C	3.79498300	-2.71433200	-0.82855600
H	3.58048600	-2.81909500	1.32264000
C	3.24465100	-2.19082000	-2.00835500
H	1.65180000	-0.96736500	-2.82712900
H	4.68805600	-3.34664200	-0.85609800
H	3.68284100	-2.40117400	-2.98864100
N	-1.42806900	-1.27286200	-0.61366500
N	1.33377500	1.64947500	-0.25931900
N	1.50219700	-1.07564800	-0.74389000
Fe	-0.03578200	0.14632800	-0.50264100
H	-0.69870400	3.14090800	-0.07076300
H	0.85745500	1.95748000	1.75527500
H	-1.40420200	0.58505300	2.27512300
C	-0.28059800	0.50111100	-2.21195900
O	-0.51287000	0.71537000	-3.33267400

L = p-NO_2-Pyridine
M = Fe

[Fe-S]$^{2+}$

	x	y	z
C	-0.85239500	0.92331500	-3.86666600
C	-0.87621900	0.24158500	-2.64410200
C	-0.10611200	2.13162900	-1.48724500
C	-0.05217800	2.86792400	-2.68524100
C	-0.42492800	2.26053900	-3.89102100
H	-1.16355700	0.40613800	-4.77932500
H	0.27152700	3.91231800	-2.67599400
H	-0.38842300	2.82254900	-4.82958900
C	2.09497300	-0.64483500	-1.06070700
C	3.47200900	-0.84379400	-1.20791700
C	2.29733100	-0.36242700	1.24051400
C	4.26221700	-0.79047900	-0.05668500
H	3.91927000	-1.03332400	-2.18773900
C	3.68302100	-0.54871400	1.19140800
H	1.80189400	-0.17037500	2.19359600
H	4.29694300	-0.50597800	2.09538800
C	-0.29515000	-2.89666200	-0.59511400
C	-0.80262900	-4.14077400	-0.99005700
C	-2.44789200	-1.98177900	-0.43129900
C	-2.19152000	-4.29678500	-1.12009600
H	-0.11323500	-4.96723700	-1.18730900
C	-3.01948700	-3.20472200	-0.82917600
H	-2.62406100	-5.25319000	-1.43064500
H	-4.10558000	-3.30843400	-0.90156100
N	-1.08572100	-1.82403600	-0.33882000
N	-0.50230500	0.81676800	-1.47237200
C	-3.22123300	-0.78587000	-0.05500000
C	-4.62491400	-0.69749600	-0.10189400
C	-3.07642700	1.40256600	0.77619500
H	-5.22045200	-1.54296500	-0.45706200
C	-4.46909800	1.55002600	0.75955400
H	-2.42884400	2.21278800	1.11778200
H	-4.91655400	2.49061400	1.09446100
C	0.20547000	2.67798500	-0.15658100
C	0.64780900	3.99086200	0.08799900
C	0.19262300	2.21557200	2.14344000
C	0.86618600	4.41485100	1.40559300
H	0.82213400	4.67912500	-0.74368000
C	0.62451800	3.51125300	2.45277300
H	-0.01394700	1.48497900	2.92935300
H	1.21230700	5.43293000	1.61003400
H	0.76747900	3.79850100	3.49889300
N	-2.45507000	0.26629000	0.37780300
N	1.49353500	-0.40802300	0.14061800
N	-0.00961800	1.79104900	0.87003300
Fe	-0.50008600	-0.03287100	0.30067800
H	0.77911400	-2.74513200	-0.47331400
H	1.44024800	-0.67908700	-1.93401000
H	-1.19317200	-0.80273200	-2.58899100
N	-0.57119300	-0.76838000	2.06774000
C	-0.71498000	-1.29560100	3.10730500
C	-0.89172700	-1.94933000	4.39917400
H	-1.08370800	-3.02781200	4.25758400
H	0.01482500	-1.83398400	5.01950400
H	-1.74751800	-1.50698600	4.93947500
C	-5.25956000	0.48199700	0.30808900
H	-6.35058400	0.56383100	0.27616800
N	5.74255900	-0.99450400	-0.16161000
O	6.19293400	-1.20352700	-1.29638300
O	6.38488800	-0.93535800	0.89590100

[Fe-H]$^{+}$

	x	y	z
C	1.38842800	0.73677600	3.77745100
C	1.08435400	0.06509400	2.58826500
C	0.72082400	2.08114400	1.45036800
C	1.01905200	2.81887600	2.61250200
C	1.35691500	2.14259700	3.79131400
H	1.64630100	0.16441600	4.67390100
H	0.99533500	3.91247800	2.59496000
H	1.59285800	2.70116900	4.70265800
C	-2.25289500	0.36060300	0.61031300

C	-3.64605700	0.27302800	0.66191200
C	-2.14081800	-1.16023500	-1.15567200
H	-4.22404300	0.84806200	1.38983900
C	-3.52806200	-1.31417100	-1.17053900
H	-1.50295700	-1.69295200	-1.86416000
H	-4.01429000	-1.98117100	-1.88700600
C	-0.03827600	-2.85054400	0.64426400
C	0.29370800	-4.18318100	0.90501800
C	2.10118900	-2.40732800	-0.20590800
C	1.57927000	-4.64705800	0.57709100
H	-0.45271000	-4.84242000	1.35885800
C	2.48997400	-3.73913900	0.02523100
H	1.86851200	-5.68687900	0.75806300
H	3.50490000	-4.06287100	-0.22287500
N	0.82660000	-1.96211100	0.07711100
N	0.75321800	0.70667300	1.43934500
C	2.97896800	-1.35765100	-0.72771600
C	4.32301600	-1.54338000	-1.10047000
C	3.11150800	0.92130400	-1.25350300
C	5.07269900	-0.45675800	-1.56324300
H	4.77854900	-2.53519300	-1.03029800
C	4.45171800	0.80151200	-1.63323600
H	2.59895100	1.88345500	-1.30747100
H	6.11789800	-0.58785900	-1.86036300
H	4.99283700	1.68672000	-1.98107800
C	0.35067700	2.67171300	0.15304800
C	0.15182600	4.04743000	-0.07471400
C	-0.15081700	2.19521400	-2.09626500
C	-0.20808100	4.49520600	-1.35106900
H	0.27032100	4.76226200	0.74492800
C	-0.36302800	3.54820200	-2.37912500
H	-0.25133100	1.41933800	-2.85907200
H	-0.36865200	5.56145300	-1.53970000
H	-0.64636300	3.84830000	-3.39254800
N	2.36860200	-0.12813900	-0.81439400
N	-1.48349300	-0.33786000	-0.27782500
N	0.20216100	1.74861100	-0.85957600
Fe	0.46433700	-0.11624200	-0.38560900
H	0.31251300	-0.53136000	-1.84594300
H	-1.02864500	-2.46295400	0.89161000
H	-1.71548400	1.00662100	1.30715900
H	1.10024700	-1.02806900	2.53469900
C	-4.28367400	-0.58030200	-0.24617400
N	-5.75928500	-0.70755100	-0.22831000
O	-6.37629200	-0.02980900	0.61334700
O	-6.27078600	-1.48410700	-1.05455500

$[Fe\text{-}CO]^{2+}$

	x	y	z
C	1.02349900	0.21433800	3.79611500
C	0.97849900	-0.26095800	2.47923800
C	0.25588100	1.81746700	1.66595700
C	0.27374200	2.35513400	2.96696400
C	0.65778400	1.54670700	4.04483100
H	1.33815100	-0.45308000	4.60401400
H	-0.00192100	3.39881000	3.14018700
H	0.67621800	1.95329400	5.06112400
C	-2.06205100	-0.79384000	0.83157800
C	-3.43608500	-0.99432900	1.00389500

C	-2.35004100	-0.17024000	-1.39405800
C	-4.26945300	-0.75817800	-0.09207900
H	-3.84800900	-1.32379000	1.96209300
H	-1.89816500	0.14863400	-2.33552100
C	0.24858400	-3.01199800	-0.08945800
C	0.73511200	-4.31031300	0.10745200
C	2.42126700	-2.11795200	-0.10619600
C	2.12085900	-4.50702800	0.20925200
H	0.03201300	-5.14632300	0.16836200
C	2.96941400	-3.39783100	0.09557300
H	2.53671400	-5.50837700	0.35982200
H	4.05314200	-3.53216400	0.14841200
N	1.06200000	-1.92975900	-0.17538400
N	0.59505100	0.51092600	1.43325300
C	3.21712800	-0.89221000	-0.29619900
C	4.62226800	-0.83866300	-0.25099500
C	3.11320300	1.40053300	-0.79851900
C	5.27785400	0.37609900	-0.49026500
H	5.20284400	-1.73971400	-0.03592100
C	4.50877800	1.51476600	-0.77412600
H	2.48010900	2.26226300	-1.02034500
H	6.37084700	0.42913100	-0.46272900
H	4.97441600	2.48329300	-0.97918900
C	-0.08543600	2.58257200	0.45302600
C	-0.48864100	3.93053400	0.45683700
C	-0.19851300	2.52620000	-1.89135300
C	-0.75259500	4.58323900	-0.75464100
H	-0.59710000	4.47037300	1.40137000
C	-0.59525100	3.86766600	-1.95152200
H	-0.05948500	1.94920300	-2.80924700
H	-1.06894400	5.63113000	-0.76261800
H	-0.77521600	4.33217900	-2.92575600
N	2.47507600	0.23031000	-0.55885400
N	-1.50517400	-0.38540900	-0.34459200
N	0.04837600	1.88002300	-0.72076300
Fe	0.50532600	-0.03745500	-0.51685900
H	-0.82211600	-2.82395300	-0.19067100
H	-1.37620200	-0.96825900	1.66246600
H	1.24792100	-1.29468300	2.24549500
C	0.54074800	-0.41784700	-2.23936700
O	0.63332500	-0.69448900	-3.36610200
C	-3.73613300	-0.34373200	-1.31449100
H	-4.38160200	-0.16507900	-2.17929000
N	-5.75114800	-0.95532200	0.04571200
O	-6.43439600	-0.71046700	-0.95634800
O	-6.15316200	-1.34320100	1.15069800

L = m-NO_2-Pyridine
M = Fe

$[Fe\text{-}S]^{2+}$

	x	y	z
C	1.97721200	1.88208400	3.29362300
C	1.64882700	0.91480900	2.33653100
C	0.38301200	2.45688700	1.10072700
C	0.67244800	3.47102400	2.03248800
C	1.47506600	3.18472400	3.14380600
H	2.61645800	1.60988900	4.13892100
H	0.28088600	4.48152100	1.88674600
H	1.70936500	3.96529500	3.87451900
C	-1.62149600	-0.35591600	2.11270300

C	-2.86614300	-0.52972000	2.73192800
C	-2.53544300	-0.67770000	0.00373800
C	-4.00144200	-0.79042100	1.94888200
H	-2.93968100	-0.45954000	3.82150100
C	-3.80029700	-0.86092000	0.56736400
H	-2.41384500	-0.73473300	-1.07733500
C	0.73024900	-2.63189100	1.39629700
C	1.45215600	-3.73972600	1.85722600
C	2.60197500	-1.78245800	0.26798300
C	2.80745800	-3.85850200	1.51147200
H	0.95097700	-4.49323600	2.47224200
C	3.38256100	-2.87003100	0.70233400
H	3.40365100	-4.70962500	1.85551100
H	4.43112500	-2.95113800	0.40313000
N	1.28329700	-1.65520800	0.63353700
N	0.86035400	1.17868800	1.26259800
C	3.08835700	-0.71036700	-0.61713700
C	4.40738200	-0.60885400	-1.09682100
C	2.47220500	1.21861800	-1.80073300
H	5.16049000	-1.34513100	-0.80316600
C	3.76589800	1.37168100	-2.31468400
H	1.67768600	1.92129500	-2.06075900
H	3.98505700	2.20869400	-2.98435600
C	-0.40405400	2.65141100	-0.12766400
C	-1.01646200	3.86040800	-0.50427300
C	-1.13756800	1.62253100	-2.10425500
C	-1.70926800	3.93794200	-1.72002500
H	-0.95591900	4.73663100	0.14714800
C	-1.76421100	2.79784900	-2.53727500
H	-1.15289500	0.71723500	-2.71553800
H	-2.19277400	4.87170600	-2.02393500
H	-2.28645500	2.80842000	-3.49872800
N	2.12962500	0.20561800	-0.96846500
N	-1.43732600	-0.42970100	0.76150600
N	-0.47253700	1.53396900	-0.92395200
Fe	0.35446400	-0.08454400	-0.15408600
H	-0.32874700	-2.51670700	1.63610700
H	-0.73009600	-0.15214300	2.71075400
H	2.01619900	-0.11049600	2.42484000
N	-0.10588300	-1.23248100	-1.61796600
C	-0.30250200	-1.99238100	-2.49138400
C	-0.56382700	-2.93139500	-3.57588500
H	-0.17662200	-3.93250400	-3.31568000
H	-1.65030200	-3.01133300	-3.75942900
H	-0.07147000	-2.59326800	-4.50493200
C	4.75437300	0.44334200	-1.95395000
H	5.77699000	0.53419200	-2.33361300
H	-4.99786200	-0.93375900	2.37766500
N	-4.94397800	-1.13578200	-0.35245400
O	-6.05834900	-1.26348700	0.16496000
O	-4.67939200	-1.21238900	-1.56584800

[Fe-H]$^{+}$

	x	y	z
C	-2.26404500	-0.54063100	3.55475300
C	-1.49513500	0.01919500	2.52889700
C	-1.68117700	-1.91930900	1.22500400
C	-2.46300900	-2.54093100	2.21814700
C	-2.76079000	-1.84704900	3.39740100
H	-2.46997500	0.04154500	4.45834400
H	-2.84306700	-3.55584100	2.06800400
H	-3.36986200	-2.31480300	4.17741900
C	1.84416400	-1.47354400	1.12358800
C	3.13473600	-1.94168100	1.40205700
C	2.65158900	0.10214500	-0.38792800
H	3.26777100	-2.75571300	2.12127000
C	3.95875200	-0.32377300	-0.13924500
H	2.44355300	0.90206300	-1.10025000
C	0.98532700	2.36163800	1.21261600
C	1.12855200	3.70515800	1.57017100
C	-0.91534400	2.83103200	-0.07618400
C	0.21916300	4.65077000	1.06527200
H	1.94729700	3.99889800	2.23431800
C	-0.81811500	4.19756200	0.24256900
H	0.31012500	5.71222700	1.31540900
H	-1.55479900	4.90368700	-0.15126300
N	0.00223000	1.90883800	0.38301700
N	-1.19824900	-0.64081900	1.38027300
C	-1.98225100	2.23314600	-0.88061600
C	-3.04326300	2.94611800	-1.46854100
C	-2.83270200	0.21425000	-1.71092300
C	-4.02175500	2.26206100	-2.19743500
H	-3.10001000	4.03262700	-1.35706300
C	-3.91297700	0.86592300	-2.31298000
H	-2.71543600	-0.86781000	-1.79455700
H	-4.85032300	2.80462000	-2.66321100
H	-4.65270600	0.27966700	-2.86666000
C	-1.29806600	-2.53955100	-0.05408900
C	-1.59041000	-3.86746700	-0.42177300
C	-0.19622900	-2.18182700	-2.10294500
C	-1.17014900	-4.35260000	-1.66553800
H	-2.13903000	-4.51852700	0.26527200
C	-0.45745000	-3.49089700	-2.51904400
H	0.34559400	-1.46974200	-2.73025600
H	-1.39073000	-5.38282200	-1.96259700
H	-0.10526000	-3.82381100	-3.50015600
N	-1.86917200	0.86784100	-1.00952100
N	1.58199200	-0.46570400	0.23922300
N	-0.60510600	-1.69639000	-0.89755200
Fe	-0.25682300	0.09544000	-0.24397400
H	0.35358700	0.48317800	-1.58802900
H	1.67507900	1.60533500	1.59376300
H	0.97356500	-1.90740700	1.62053300
H	-1.09330300	1.03400700	2.61096200
C	4.23730300	-1.36237700	0.75887500
H	5.26647800	-1.68747900	0.93231900
N	5.07256100	0.34969500	-0.86197000
O	4.77309400	1.27329300	-1.63719200
O	6.22049900	-0.06125900	-0.63120600

[Fe-CO]$^{2+}$

	x	y	z
C	1.83432300	0.82666800	3.61131100
C	1.55872400	0.16744600	2.40650100
C	0.26611700	1.96204200	1.62376200
C	0.50018600	2.67548000	2.81456500
C	1.28984700	2.10357000	3.82060800
H	2.46331300	0.34213800	4.36413500
H	0.07858100	3.67440100	2.95504400
H	1.48103600	2.64966800	4.74998500
C	-1.61122800	-1.15376700	1.68981200
C	-2.83953900	-1.54961000	2.23407900

C	-2.57062800	-0.75038600	-0.38839900
C	-3.98894200	-1.54466600	1.42854200
H	-2.88922200	-1.85715900	3.28312800
H	-2.48097800	-0.43826800	-1.42997000
C	0.80075200	-3.00267100	0.36937100
C	1.57137000	-4.16028900	0.53222300
C	2.67312700	-1.75835600	-0.31166400
C	2.94888800	-4.10000800	0.27054800
H	1.08875000	-5.08993300	0.84812400
C	3.50040300	-2.88665100	-0.16139700
H	3.58169200	-4.98599700	0.38403400
H	4.56637200	-2.82470800	-0.39606400
N	1.33156300	-1.81678400	-0.02144900
N	0.78762700	0.70951800	1.43284000
C	3.13042500	-0.45308500	-0.81957400
C	4.46095500	-0.15516600	-1.16624400
C	2.44343100	1.69528200	-1.47563900
C	4.77647300	1.10782100	-1.68384400
H	5.24640000	-0.90484700	-1.03881700
C	3.74751800	2.04778900	-1.84532000
H	1.61558700	2.39805100	-1.59034000
H	5.80708900	1.34951400	-1.96242600
H	3.94112400	3.04351100	-2.25513600
C	-0.50771500	2.48005000	0.48082800
C	-1.15595500	3.72861100	0.46514200
C	-1.18218200	2.07166900	-1.73159000
C	-1.83584700	4.14752700	-0.68647100
H	-1.13319300	4.37235800	1.34865300
C	-1.84147200	3.30470300	-1.80814700
H	-1.16529300	1.40014900	-2.59389100
H	-2.34670900	5.11541900	-0.70731900
H	-2.34889100	3.58585500	-2.73587400
N	2.13484200	0.47794600	-0.96832700
N	-1.45837200	-0.76091700	0.39129700
N	-0.53079600	1.64987400	-0.61474000
Fe	0.33604400	-0.11398400	-0.36240700
H	-0.27683300	-3.01816200	0.54499500
H	-0.70920700	-1.14826600	2.30566900
H	1.95836800	-0.83077300	2.20763500
C	-0.00017300	-0.73134800	-1.98279300
O	-0.15570100	-1.15652900	-3.05432300
C	-3.82154800	-1.12890900	0.10500700
H	-4.97434200	-1.84492700	1.79822000
N	-4.99209600	-1.07243400	-0.82525100
O	-6.07288900	-1.46977900	-0.37862700
O	-4.77566100	-0.62335800	-1.96268400

C	-1.61573900	2.13110100	-1.43505600
C	-2.52554600	3.14967100	-1.74054700
C	-1.98044600	2.39681900	0.84869300
C	-3.18656000	3.81467000	-0.69558800
H	-2.70888900	3.40934400	-2.78787300
C	-2.90352800	3.42436200	0.62256300
H	-1.73863100	2.07062500	1.86280000
H	-3.90360500	4.61541300	-0.90345700
H	-3.39041600	3.90633600	1.47604100
C	1.83337300	2.32517800	-1.03224200
C	3.02194900	2.88295400	-1.51678700
C	2.91117500	0.30772100	-0.47982400
C	4.19521600	2.11213500	-1.48378800
H	3.02007100	3.90511600	-1.90715500
C	4.13477800	0.81610600	-0.95620300
H	5.14304400	2.51439500	-1.85514800
H	5.04078700	0.20595500	-0.90969000
N	1.76143900	1.06335200	-0.53511600
N	-0.54743400	-0.82817100	-1.46363900
C	2.75147700	-1.03209700	0.11917400
C	3.79487800	-1.96868600	0.24667300
C	1.25538100	-2.52786500	1.15016500
C	3.54890800	-3.21229900	0.84195300
H	4.79740500	-1.73023400	-0.11830800
C	2.25401200	-3.49585800	1.30438800
H	0.23358300	-2.70302500	1.49640800
H	4.35501700	-3.94583400	0.94260100
H	2.01245700	-4.45183100	1.77854000
C	-2.29020500	-1.63117000	-0.01309300
C	-3.46050400	-2.34234800	0.31195000
C	-2.06581300	-0.93412900	2.22333900
C	-3.93550600	-2.34141200	1.62976500
H	-4.00220300	-2.89512600	-0.46032400
C	-3.21916600	-1.62838500	2.60488700
H	-1.47379600	-0.36766500	2.94715800
H	-4.84557300	-2.89078000	1.89022200
H	-3.54467200	-1.60460600	3.64928800
N	1.48523800	-1.32340400	0.56944200
N	-1.32979400	1.74935900	-0.15795000
N	-1.60217400	-0.92176400	0.94616100
Ru	0.04568600	0.15204300	0.26041400
H	0.89947800	2.89293100	-1.02869600
H	-1.09005300	1.59332600	-2.22851800
H	1.00668000	-0.17042400	-2.68553600
N	0.56637900	0.99988500	2.03932500
C	0.96342400	1.47143700	3.04037300
C	1.45763800	2.05472000	4.28293800
H	2.28781800	2.75254500	4.07362900
H	0.65387700	2.61160900	4.79648100
H	1.82507500	1.26294800	4.95983000

L = pyridine
M = Ru

$[Ru\text{-}S]^{2+}$

	x	y	z
C	-0.34542800	-1.47195400	-3.78979300
C	0.10263900	-0.78482200	-2.65703100
C	-1.68580900	-1.59661000	-1.35771700
C	-2.18557500	-2.30300300	-2.46806500
C	-1.51662600	-2.24140900	-3.69672100
H	0.21858900	-1.39923500	-4.72454300
H	-3.09226400	-2.90628100	-2.37266900
H	-1.89995700	-2.78913500	-4.56330200

$[Ru\text{-}H]^{+}$

	x	y	z
C	0.31802700	-0.94485900	3.88412400
C	-0.10196600	-0.35633600	2.68653900
C	1.65216600	-1.34570700	1.48085300
C	2.13025400	-1.96485100	2.65472400
C	1.46021200	-1.76530100	3.86767700
H	-0.24009200	-0.76186700	4.80768600
H	3.01381900	-2.60871800	2.62237600
H	1.82088500	-2.24439100	4.78358300

C	2.00787000	2.18296200	0.56260100
C	2.81885500	3.32357400	0.58728000
C	0.99372900	2.86388300	-1.42075600
C	2.70174800	4.27320200	-0.43963700
H	3.52948900	3.45665300	1.40936800
C	1.76601700	4.03024300	-1.45781400
H	0.26256400	2.63208800	-2.19870100
H	3.32239700	5.17520800	-0.44721600
H	1.63117600	4.73188100	-2.28719200
C	-1.98097000	2.42131400	0.46341300
C	-3.20880400	3.02019000	0.75229300
C	-3.01925900	0.38827200	-0.10259500
C	-4.39025200	2.27276900	0.59601600
H	-3.23198600	4.06042500	1.09175600
C	-4.28342200	0.94451800	0.17044600
H	-5.36975500	2.71310200	0.80643000
H	-5.18403800	0.33551900	0.05097400
N	-1.86299000	1.13417700	0.02886200
N	0.53949900	-0.54049200	1.50551800
C	-2.79746500	-1.00092400	-0.52202000
C	-3.82293200	-1.94160800	-0.73775400
C	-1.17540000	-2.61824900	-1.05802400
C	-3.50653200	-3.24857500	-1.12218200
H	-4.86844100	-1.64844700	-0.60776500
C	-2.15190600	-3.59158700	-1.27943300
H	-0.11226300	-2.83902200	-1.18095500
H	-4.29838300	-3.98394000	-1.29551800
H	-1.84897500	-4.60048400	-1.57601800
C	2.27328000	-1.50872600	0.15020000
C	3.46542700	-2.22734300	-0.07072600
C	2.11231200	-1.04248600	-2.15461600
C	3.98378600	-2.35105400	-1.36409900
H	3.99128800	-2.68498100	0.77188700
C	3.28785000	-1.74673000	-2.42619300
H	1.53352200	-0.55420800	-2.94205400
H	4.91060000	-2.90626600	-1.53972400
H	3.64837000	-1.81412000	-3.45724900
N	-1.47137500	-1.34121600	-0.69222600
N	1.09550300	1.93563700	-0.42169200
N	1.59830300	-0.91122300	-0.89723700
Ru	-0.10561500	0.18099600	-0.45961600
H	-0.39849900	0.54537400	-2.02145100
H	-1.04163200	2.96963800	0.56710900
H	2.07199900	1.43195200	1.35385500
H	-0.98329600	0.29304700	2.64746400

$[Ru\text{-}CO]^{2+}$

	x	y	z
C	-0.15452400	-0.76735000	3.87886500
C	-0.43268600	-0.24809300	2.60928000
C	1.38556300	-1.38819300	1.64537600
C	1.71828500	-1.94014600	2.89830400
C	0.94612900	-1.62694500	4.02456800
H	-0.78948000	-0.50048500	4.72894200
H	2.57079800	-2.61654300	2.99857800
H	1.19894300	-2.05295100	5.00083300
C	1.58399400	2.31494300	1.01474900
C	2.50905100	3.33820400	1.25041000
C	2.17061000	2.21302200	-1.23794400
C	3.29354200	3.81249700	0.18716000
H	2.60575000	3.75117700	2.25934800
C	3.11579500	3.23247900	-1.07822800
H	2.01305300	1.75190400	-2.21568800
H	4.02297100	4.61432900	0.34070500
H	3.69555000	3.56363100	-1.94529700
C	-1.80431300	2.63134200	0.18858700
C	-2.99032900	3.32042900	0.46797600
C	-2.94623800	0.57907200	-0.00192300
C	-4.19380900	2.60020500	0.51844900
H	-2.96223000	4.40176600	0.63160700
C	-4.16780600	1.21996100	0.27823800
H	-5.14175900	3.10507100	0.73000600
H	-5.09951400	0.64878400	0.29601400
N	-1.77103600	1.29289800	-0.02814900
N	0.31653800	-0.53938100	1.51795500
C	-2.82246800	-0.86076600	-0.31351700
C	-3.90618100	-1.75902900	-0.32744600
C	-1.35667900	-2.58772000	-0.95880600
C	-3.69557100	-3.10119500	-0.66993000
H	-4.91305900	-1.41471800	-0.07746500
C	-2.39751900	-3.52303600	-0.99557900
H	-0.32962900	-2.86669400	-1.20730300
H	-4.53471500	-3.80385900	-0.68704300
H	-2.18546300	-4.55828400	-1.27863400
C	2.11866800	-1.68201200	0.39474300
C	3.26207100	-2.50159600	0.34116600
C	2.21438400	-1.37444900	-1.93728100
C	3.88970700	-2.75542700	-0.88509000
H	3.66748300	-2.94065500	1.25638500
C	3.34976300	-2.18470100	-2.04740400
H	1.76515000	-0.91913400	-2.82332200
H	4.78058000	-3.38969400	-0.93020500
H	3.79418200	-2.35721300	-3.03225800
N	-1.55523300	-1.29094600	-0.62141400
N	1.40083400	1.74891800	-0.21218500
N	1.60338300	-1.11387800	-0.74984400
Ru	-0.04207700	0.16760600	-0.49702700
H	-0.84551200	3.15158500	0.12306200
H	0.96228900	1.92650100	1.82523500
H	-1.27761700	0.42782500	2.44513200
C	-0.28151700	0.59956300	-2.30845600
O	-0.44441400	0.83167200	-3.44144900

L = pyridine
M = Co

$[Co\text{-}S]^{3+}$

	x	y	z
C	-0.15522900	-1.20172400	-3.78218300
C	0.26239900	-0.57300200	-2.60186800
C	-1.57127200	-1.45006300	-1.40537700
C	-2.03957900	-2.09600600	-2.56385900
C	-1.33001200	-1.97044000	-3.76619400
H	0.43877200	-1.08084400	-4.69351300
H	-2.94981600	-2.70122200	-2.52564100
H	-1.68551500	-2.47051700	-4.67331800
C	-1.54721800	2.09097000	-1.34207300
C	-2.46748100	3.11556100	-1.59266900
C	-1.91101300	2.24340800	0.96059000
C	-3.13345700	3.72132200	-0.51499500
H	-2.65239900	3.42543200	-2.62624800
C	-2.84342200	3.27235400	0.78305100

H	-1.67124600	1.87752600	1.95981700
H	-3.85988300	4.52359300	-0.68300800
H	-3.33096400	3.70866200	1.66068000
C	1.68492900	2.39783500	-0.79946600
C	2.87861200	2.99990700	-1.22104200
C	2.77754600	0.34422100	-0.40849700
C	4.05486000	2.23413700	-1.24502100
H	2.87349500	4.05266600	-1.52031400
C	4.00186800	0.89610900	-0.82767400
H	5.00323800	2.67318600	-1.57260700
H	4.91226600	0.29022900	-0.81950300
N	1.62808100	1.09626300	-0.42486900
N	-0.43262200	-0.68392600	-1.44238300
C	2.60616100	-1.02792700	0.09621600
C	3.63380200	-1.98482200	0.18239300
C	1.06797900	-2.55226000	1.02790300
C	3.35776500	-3.25534400	0.70766800
H	4.64441800	-1.74274400	-0.15829000
C	2.05477800	-3.54123800	1.14297100
H	0.04386900	-2.73934100	1.35596000
H	4.15022500	-4.00791300	0.77786900
H	1.79247700	-4.51597500	1.56588600
C	-2.19546800	-1.54593100	-0.07873400
C	-3.36989300	-2.26184600	0.21435100
C	-1.95229500	-0.94872700	2.18808300
C	-3.83850500	-2.31222100	1.53543900
H	-3.91689200	-2.77681500	-0.58050100
C	-3.11221700	-1.65251700	2.54013800
H	-1.35494600	-0.42876000	2.93991200
H	-4.75300800	-2.86450600	1.77632500
H	-3.43109400	-1.67706500	3.58688300
N	1.33552000	-1.32848200	0.51500200
N	-1.26144000	1.65544500	-0.08202800
N	-1.50673100	-0.89042100	0.90957700
Co	0.03754900	0.13733100	0.25805600
H	0.75325800	2.96438200	-0.75635900
H	-1.01611800	1.61096900	-2.16620900
H	1.16428900	0.04165800	-2.58207000
N	0.54592300	0.83270800	1.99222000
C	0.99351000	1.24455500	2.99325200
C	1.53346700	1.75913700	4.24221700
H	2.33456300	2.49187000	4.03460600
H	0.73992500	2.26037700	4.82602600
H	1.95245400	0.93340800	4.84574000

[Co-H]$^{2+}$

	x	y	z
C	0.01999600	0.90064500	3.81958700
C	0.35413500	0.28436500	2.60510900
C	-1.42299500	1.33365500	1.49162600
C	-1.81250400	1.98798200	2.67510900
C	-1.08186700	1.76957100	3.85240100
H	0.61568800	0.70066900	4.71528600
H	-2.67063800	2.66587800	2.68376700
H	-1.37069100	2.27216000	4.78113600
C	-1.91313800	-2.08641600	0.61648600
C	-2.74885600	-3.20989700	0.62193100
C	-1.01889700	-2.68178000	-1.45807900
C	-2.71067600	-4.09820200	-0.46395500
H	-3.41648200	-3.37644000	1.47295300
C	-1.82633600	-3.82312800	-1.51894500
H	-0.31567400	-2.44186700	-2.25897100
H	-3.35425300	-4.98372000	-0.48772300
H	-1.75423300	-4.48155300	-2.39019500
C	1.80049600	-2.43776300	0.42012400
C	3.03006400	-3.06744400	0.65013100
C	2.86285900	-0.44388300	-0.22821400
C	4.21525200	-2.35376300	0.41369300
H	3.04605700	-4.10274600	1.00343600
C	4.12641500	-1.02593700	-0.02369100
H	5.19292200	-2.81994300	0.57189400
H	5.03571100	-0.44621100	-0.20407500
N	1.70939200	-1.15769900	-0.01888400
N	-0.34562100	0.48875500	1.46709700
C	2.63663800	0.94479600	-0.65233900
C	3.65389800	1.87432400	-0.93168700
C	0.98959700	2.55102900	-1.12417900
C	3.31458800	3.17714100	-1.31805200
H	4.70438100	1.58201400	-0.85202700
C	1.95774800	3.52141900	-1.41090800
H	-0.07449000	2.78147700	-1.19865600
H	4.09664200	3.90904100	-1.54346400
H	1.64134400	4.52580500	-1.70739300
C	-2.11301700	1.47696700	0.19098300
C	-3.29759100	2.21058700	-0.00641300
C	-2.06836500	0.88861600	-2.09414000
C	-3.87013000	2.27715500	-1.28416900
H	-3.77520900	2.72154600	0.83407800
C	-3.24497200	1.60515500	-2.34641500
H	-1.54146300	0.35160300	-2.88650300
H	-4.79242700	2.84445200	-1.44568500
H	-3.65540800	1.62905600	-3.36024900
N	1.31305400	1.28954400	-0.75353400
N	-1.05322500	-1.81545100	-0.40354600
N	-1.51136800	0.82641500	-0.85875100
Co	0.07854000	-0.18420400	-0.42448000
H	0.34244300	-0.48622200	-1.83976500
H	0.85816000	-2.96183800	0.58893600
H	-1.91665500	-1.38028500	1.44905000
H	1.20401900	-0.40146300	2.53248600

[Co-CO]$^{3+}$

	x	y	z
C	-0.28151400	-0.49431900	3.78799300
C	-0.55105600	-0.05757100	2.48376600
C	1.31582200	-1.22311000	1.63346700
C	1.63752900	-1.68374200	2.92318400
C	0.83486000	-1.31594400	4.01286700
H	-0.94172100	-0.18734400	4.60536900
H	2.50445100	-2.33259100	3.07722700
H	1.07523100	-1.67123500	5.02064700
C	1.51650900	2.26745400	0.92522500
C	2.45177800	3.29822800	1.07853000
C	2.08313600	2.01474800	-1.33179700
C	3.22648600	3.69774200	-0.02257900
H	2.56207500	3.77400500	2.05850200
C	3.03172500	3.03994700	-1.24771700
H	1.93131100	1.49027200	-2.27799300
H	3.96321700	4.50293200	0.07127300
H	3.60211800	3.31138000	-2.14203900
C	-1.64576400	2.61830200	0.02506100

C	-2.83539800	3.33169800	0.22219700
C	-2.81578600	0.56502000	-0.08181500
C	-4.04976600	2.62870200	0.27580400
H	-2.79757800	4.42136900	0.31890300
C	-4.03655700	1.23459200	0.11796300
H	-4.99742300	3.15794900	0.42291500
H	-4.97573400	0.67445800	0.13451800
N	-1.63216900	1.26626600	-0.09908700
N	0.23306300	-0.40395000	1.43340000
C	-2.68101600	-0.88124600	-0.31772000
C	-3.74944300	-1.79616400	-0.32812600
C	-1.16012000	-2.61205100	-0.84447000
C	-3.50506800	-3.14873100	-0.60799600
H	-4.76882800	-1.45603800	-0.12462500
C	-2.19035700	-3.56189600	-0.87433500
H	-0.12610800	-2.89445000	-1.05047600
H	-4.33199000	-3.86673700	-0.62413200
H	-1.95202200	-4.60432000	-1.10808100
C	2.04722300	-1.57531900	0.40758300
C	3.19378700	-2.38909200	0.35868900
C	2.06543100	-1.36874200	-1.94538400
C	3.78358500	-2.68991600	-0.87781800
H	3.62635900	-2.78655700	1.28134400
C	3.20366800	-2.17826800	-2.05006100
H	1.58880800	-0.96853300	-2.84419200
H	4.67830600	-3.31999600	-0.92474100
H	3.61707700	-2.39720500	-3.03990100
N	-1.40051600	-1.30791500	-0.56812100
N	1.32452800	1.62893400	-0.26461900
N	1.50146800	-1.06754400	-0.74614800
Co	-0.03074000	0.12825600	-0.44594100
H	-0.68499800	3.13200400	-0.03994800
H	0.90253200	1.94711200	1.76877700
H	-1.40473400	0.59121600	2.27461900
C	-0.37593700	0.50804300	-2.19919200
O	-0.68843800	0.72983600	-3.28068100

[Fe(tpy)(bpy)(S)]$^{2+}$

	x	y	z
C	0.73488200	-0.00248900	-3.90971900
C	0.11010200	-0.00146600	-2.65719600
C	2.17360400	0.00047600	-1.54200400
C	2.85975400	-0.00063500	-2.77033500
C	2.13709200	-0.00216700	-3.96959300
H	0.12389700	-0.00352800	-4.81728500
H	3.95296100	-0.00028100	-2.79037800
H	2.65931400	-0.00305100	-4.93152200
C	-2.40458800	-1.19522900	-0.41989700
C	-3.75850100	-1.22391400	-0.79996600
C	-2.40798500	1.18856300	-0.42150600
C	-4.43380400	-0.00660400	-0.99321000
H	-4.28197300	-2.17391100	-0.94023100
C	-3.76194200	1.21289500	-0.80162700
H	-5.48748100	-0.00827800	-1.28938800
H	-4.28807600	2.16121600	-0.94327300
N	-1.76741800	-0.00232600	-0.25203000
N	0.80127500	-0.00009700	-1.49018700
C	-1.51758700	2.33471700	-0.16217500
C	-1.88745000	3.68435700	-0.28308100
C	0.65534800	2.94142300	0.48862100
C	-0.94477400	4.68507500	-0.00585500
H	-2.90237800	3.95118200	-0.59253500
C	0.34802800	4.30577700	0.38701300
H	1.64988100	2.60536700	0.79416900
H	-1.21649700	5.74160900	-0.09557000
H	1.11630300	5.05100400	0.61401900
C	2.82261700	0.00262900	-0.22249700
C	4.21457900	0.00412900	-0.01518600
C	2.44250900	0.00607400	2.09239700
C	4.72189000	0.00665900	1.29030800
H	4.89926300	0.00338600	-0.86783700
C	3.81543200	0.00771700	2.36350600
H	1.70712400	0.00682000	2.89931800
H	5.80230400	0.00783900	1.46566000
H	4.16068500	0.00977300	3.40180200
N	-0.24638200	1.96835100	0.22323100
N	1.93700700	0.00347000	0.83031400
Fe	0.02605600	0.00060300	0.31842600
H	-0.97904600	-0.00176000	-2.57614300
C	-1.51105200	-2.33854800	-0.15889400
C	-1.87720600	-3.68936100	-0.27802900
C	0.66329200	-2.93839800	0.49342000
C	-0.93188200	-4.68711000	0.00082600
H	-2.89131000	-3.95935600	-0.58743200
C	0.35974500	-4.30371800	0.39361700
H	1.65673700	-2.59915700	0.79893900
H	-1.20067200	-5.74450800	-0.08752300
H	1.12997500	-5.04652400	0.62192800
N	-0.24097000	-1.96814900	0.22630300
N	-0.59032900	0.00114400	2.13337400
C	-1.05222800	0.00142900	3.21160700
C	-1.61298000	0.00176100	4.55787100
H	-2.24212500	-0.89289800	4.71066000
H	-0.80633300	-0.00339500	5.31226100
H	-2.23366400	0.90177700	4.71375100

[Fe(tpy)(bpy)(H)]$^{+}$

	x	y	z
C	-0.58386400	-0.00069100	3.77262700
C	0.01559200	-0.00041100	2.50819000
C	-2.06758600	-0.00096200	1.43266300
C	-2.73459900	-0.00126000	2.67307900
C	-1.98734100	-0.00111600	3.85720000
H	0.04107200	-0.00057900	4.67096500
H	-3.82780900	-0.00164100	2.71398500
H	-2.49081800	-0.00135800	4.82925400
C	2.55265800	-1.19066600	-0.15468300
C	3.94651800	-1.21287400	0.01668600
C	2.55128000	1.19335400	-0.15466300
C	4.64553100	0.00255700	0.10988100
H	4.48353000	-2.16487700	0.06541100
C	3.94510500	1.21719100	0.01671900
H	5.73192000	0.00319000	0.24047600
H	4.48101600	2.16980800	0.06549500
N	1.87490800	0.00096500	-0.21911600
N	-0.69820800	-0.00051500	1.35632100
C	1.63887400	2.32515700	-0.32019700
C	2.00820200	3.68177000	-0.29527500
C	-0.60336200	2.90146700	-0.71040400
C	1.03435600	4.66818900	-0.48613100
H	3.05386000	3.95759200	-0.12758400
C	-0.29667400	4.26559800	-0.69679500

H	-1.62583000	2.55369100	-0.87774400
H	1.30597700	5.72839800	-0.47214900
H	-1.09486900	4.99792900	-0.85213600
C	-2.72670000	-0.00118800	0.11699200
C	-4.12015000	-0.00147100	-0.08298300
C	-2.36045500	-0.00141900	-2.20639100
C	-4.63643500	-0.00172700	-1.38437100
H	-4.79697200	-0.00147000	0.77663300
C	-3.73469900	-0.00172500	-2.46408800
H	-1.62040800	-0.00140800	-3.01022700
H	-5.71796600	-0.00192600	-1.55308500
H	-4.08617600	-0.00194500	-3.50045300
N	0.33062000	1.93144600	-0.53185000
N	-1.84341100	-0.00111200	-0.94410700
Fe	0.05239900	-0.00010300	-0.50619300
H	1.10414100	-0.00008500	2.39283000
C	1.64154100	-2.32348800	-0.32025700
C	2.01234000	-3.67969700	-0.29532300
C	-0.60007700	-2.90228200	-0.71061500
C	1.03960500	-4.66718000	-0.48624800
H	3.05829900	-3.95435300	-0.12757600
C	-0.29185900	-4.26605400	-0.69700100
H	-1.62290400	-2.55561800	-0.87809200
H	1.31239500	-5.72708900	-0.47228800
H	-1.08922800	-4.99927900	-0.85239400
N	0.33283100	-1.93119700	-0.53198600
H	0.33440400	0.00017000	-2.00683400

[Fe(tpy)(bpy)(CO)]$^{2+}$

	x	y	z
C	-0.22578900	0.00059700	3.81658900
C	0.24665600	0.00019200	2.49841100
C	-1.93068400	0.00067800	1.63045600
C	-2.47052200	0.00108400	2.93057100
C	-1.61167900	0.00104500	4.03707200
H	0.48595400	0.00054500	4.64746800
H	-3.55334600	0.00149200	3.08083800
H	-2.01884100	0.00137600	5.05320600
C	2.48044900	-1.19402900	-0.01307700
C	3.86493200	-1.22066200	0.23488500
C	2.48134900	1.19207900	-0.01308500
C	4.55385500	-0.00175800	0.35477600
H	4.40124000	-2.16920500	0.32686700
C	3.86585000	1.21764400	0.23495300
H	5.63255000	-0.00219000	0.54065000
H	4.40280600	2.16580600	0.32713500
N	1.83672700	-0.00075100	-0.12928400
N	-0.57638200	0.00023100	1.42330400
C	1.56825300	2.33884800	-0.17910700
C	1.95145700	3.68739700	-0.10134600
C	-0.65941500	2.95078300	-0.61230000
C	0.98794600	4.68999500	-0.28696500
H	2.99364300	3.95279200	0.09892700
C	-0.33851100	4.31450100	-0.54638600
H	-1.67883200	2.61730800	-0.82270400
H	1.27161200	5.74587000	-0.23402700
H	-1.12270400	5.06127000	-0.70254900
C	-2.73450900	0.00081500	0.39634900
C	-4.14177900	0.00093400	0.37111300
C	-2.66524700	0.00110000	-1.95136900
C	-4.81771300	0.00119400	-0.85560500
H	-4.70901400	0.00080100	1.30577600
C	-4.06194200	0.00132500	-2.03852400
H	-2.05585400	0.00109400	-2.85812100
H	-5.91190300	0.00130000	-0.88622700
H	-4.53827300	0.00158400	-3.02356700
N	0.26271900	1.97835900	-0.43236700
N	-1.99572600	0.00082900	-0.76506900
Fe	-0.02169900	0.00005900	-0.52206100
H	1.31942600	-0.00023100	2.29030700
C	0.32860800	-0.00007800	-2.25598600
O	0.58428200	-0.00007300	-3.38985200
C	1.56636900	-2.34008100	-0.17892400
C	1.94850000	-3.68889800	-0.10097000
C	-0.66169200	-2.95024300	-0.61233600
C	0.98417400	-4.69078000	-0.28651400
H	2.99046800	-3.95510000	0.09933200
C	-0.34195700	-4.31421700	-0.54614900
H	-1.68079000	-2.61596100	-0.82301900
H	1.26697500	-5.74687100	-0.23336500
H	-1.12676900	-5.06031900	-0.70233200
N	0.26120600	-1.97853900	-0.43249500

[Ru(tpy)(bpy)(S)]$^{2+}$

	x	y	z
Ru	-0.04617700	0.00000400	0.32922300
N	1.84435600	-0.00002100	-0.30835900
C	-0.52983800	3.08503800	0.46598100
H	-1.53109700	2.79231900	0.79407100
C	-0.16583300	4.43238000	0.34070600
C	1.13485700	4.74669300	-0.08155000
H	1.45366700	5.78815800	-0.18987800
C	2.02549400	3.70129700	-0.36474100
H	3.04385700	3.92452600	-0.69571800
C	1.60701700	2.36699400	-0.22310400
C	2.47327800	1.19910600	-0.49880100
C	3.81549800	1.21873400	-0.91946000
H	4.34003900	2.16486000	-1.07875600
C	4.48198800	-0.00005400	-1.13011400
H	5.52643200	-0.00006700	-1.45705000
C	3.81546700	-1.21882500	-0.91946100
H	4.33998400	-2.16496400	-1.07875700
C	2.47324700	-1.19916400	-0.49880300
N	-2.08422800	0.00002400	0.84702500
C	-0.29013100	0.00001300	-2.72206400
H	0.80096200	0.00000700	-2.66262300
C	-0.94764100	0.00002000	-3.95587600
H	-0.36236500	0.00001900	-4.88019400
C	-2.35156600	0.00002600	-3.97537500
H	-2.90086800	0.00003000	-4.92202700
C	-3.04133300	0.00002800	-2.75735400
H	-4.13443000	0.00003400	-2.75202800
C	-2.32911700	0.00002200	-1.54292000
C	-2.96463300	0.00002700	-0.21306000
C	-4.35701200	0.00003400	-0.00162400
H	-5.04394900	0.00003600	-0.85215500
C	-4.86568900	0.00003900	1.30283500
H	-5.94640600	0.00004400	1.47563400
C	-3.96204100	0.00003700	2.37886300
H	-4.30956800	0.00004000	3.41640100
C	-2.58977100	0.00002900	2.10982700
H	-1.85029900	0.00002700	2.91429200

N	-0.95118800	0.00001300	-1.53507900
C	1.60695700	-2.36702900	-0.22310500
C	2.02539900	-3.70134300	-0.36474500
C	-0.52991100	-3.08501900	0.46599500
C	1.13473900	-4.74671700	-0.08154700
H	3.04375500	-3.92459700	-0.69572800
C	-0.16594000	-4.43236900	0.34071900
H	-1.53115800	-2.79227300	0.79409500
H	1.45352200	-5.78818900	-0.18987600
H	-0.89565300	-5.21386600	0.57289800
N	0.32251500	-2.06759700	0.19501500
H	-0.89552800	5.21389400	0.57287600
N	0.32256500	2.06759400	0.19501100
N	0.64830800	0.00000800	2.25125800
C	1.63399400	-0.00001900	4.69237900
H	1.29818100	0.89776100	5.24108900
H	1.29805800	-0.89771900	5.24114700
H	2.73804700	-0.00009600	4.66119900
C	1.09257000	-0.00002800	3.33826400

[Ru(tpy)(bpy)(H)]$^{+}$

	x	y	z
Ru	-0.52048500	-0.04798900	0.00000000
H	-2.10752300	-0.41759700	0.00000000
N	-0.14460000	-1.97969400	0.00000000
C	-0.69048100	0.44021700	3.05923600
H	-0.89747100	1.47082200	2.75800700
C	-0.64321700	0.06829900	4.40555900
C	-0.38114900	-1.27350200	4.73488800
H	-0.33961600	-1.59883300	5.77910800
C	-0.17581400	-2.19003200	3.69766100
H	0.02964200	-3.24058900	3.92360600
C	-0.23454600	-1.76330800	2.35800200
C	-0.04898200	-2.64808000	1.19938500
C	0.17799900	-4.03446800	1.21539900
H	0.25180000	-4.57284300	2.16472300
C	0.29742500	-4.72851100	0.00000000
H	0.47136500	-5.80873300	0.00000000
C	0.17799900	-4.03446800	-1.21539900
H	0.25180000	-4.57284300	-2.16472300
C	-0.04898200	-2.64808000	-1.19938500
N	-0.97794800	1.99133000	0.00000000
C	2.64006100	0.29912500	0.00000000
H	2.59998600	-0.79527500	0.00000000
C	3.86033300	0.98282800	0.00000000
H	4.80036900	0.42250000	0.00000000
C	3.84361700	2.38879200	0.00000000
H	4.77686400	2.96114700	0.00000000
C	2.61032100	3.04993600	0.00000000
H	2.57994400	4.14306300	0.00000000
C	1.41473800	2.30108900	0.00000000
C	0.06487000	2.89816100	0.00000000
C	-0.17650200	4.28705700	0.00000000
H	0.66317000	4.98775400	0.00000000
C	-1.48790600	4.77322100	0.00000000
H	-1.68121200	5.85054100	0.00000000
C	-2.54592100	3.84589200	0.00000000
H	-3.59080300	4.17128100	0.00000000
C	-2.25379000	2.48042100	0.00000000
H	-3.03950100	1.72113500	0.00000000
N	1.44062000	0.93000000	0.00000000
C	-0.23454600	-1.76330800	-2.35800200
C	-0.17581400	-2.19003200	-3.69766100
C	-0.69048100	0.44021700	-3.05923600
C	-0.38114900	-1.27350200	-4.73488800
H	0.02964200	-3.24058900	-3.92360600
C	-0.64321700	0.06829900	-4.40555900
H	-0.89747100	1.47082200	-2.75800700
H	-0.33961600	-1.59883300	-5.77910800
H	-0.81352500	0.82269900	-5.17980200
N	-0.49348000	-0.43827500	-2.04053200
H	-0.81352500	0.82269900	5.17980200
N	-0.49348000	-0.43827500	2.04053200

[Ru(tpy)(bpy)(CO)]$^{2+}$

	x	y	z
Ru	-0.03407400	0.00001100	-0.53205500
N	1.92331700	-0.00004000	-0.05999300
C	-0.51748000	-3.10069100	-0.60668800
H	-1.54489000	-2.81285100	-0.84610600
C	-0.13383000	-4.44686000	-0.52433600
C	1.20099600	-4.75288800	-0.22291200
H	1.53658700	-5.79256700	-0.15522700
C	2.10755300	-3.70286900	-0.01307300
H	3.15358900	-3.92196500	0.21896000
C	1.66922000	-2.37150200	-0.10890800
C	2.55577900	-1.19968400	0.08624400
C	3.92902900	-1.21988700	0.39273700
H	4.46585700	-2.16497900	0.51036600
C	4.61023500	-0.00011200	0.54015600
H	5.68000400	-0.00014100	0.77183300
C	3.92908900	1.21969800	0.39276400
H	4.46596200	2.16476200	0.51041500
C	2.55583800	1.19956900	0.08627200
N	-2.14104000	0.00005700	-0.76105000
C	0.05285800	-0.00006000	2.61410100
H	1.13235400	-0.00008500	2.43791400
C	-0.46562900	-0.00006600	3.91357000
H	0.21351900	-0.00009700	4.77125100
C	-1.85959800	-0.00003400	4.07908800
H	-2.30561300	-0.00004000	5.07879400
C	-2.67778000	0.00000600	2.94245200
H	-3.76433200	0.00003100	3.05958000
C	-2.09716700	0.00001200	1.65854900
C	-2.87825500	0.00005000	0.40402200
C	-4.28645900	0.00007600	0.36961400
H	-4.85944800	0.00006900	1.30034000
C	-4.96037800	0.00010900	-0.85766900
H	-6.05445700	0.00012800	-0.88881500
C	-4.20339100	0.00011500	-2.03970500
H	-4.67804800	0.00014000	-3.02555300
C	-2.80779000	0.00008800	-1.94933700
H	-2.19120800	0.00009100	-2.85164300
N	-0.73300900	-0.00002000	1.51015000
C	1.66933400	2.37143500	-0.10885200
C	2.10773200	3.70277800	-0.01299100
C	-0.51733300	3.10074100	-0.60660600
C	1.20122600	4.75284500	-0.22280300
H	3.15378000	3.92181800	0.21904300
C	-0.13361700	4.44688900	-0.52422700
H	-1.54475800	2.81295600	-0.84602700
H	1.53686700	5.79250700	-0.15509800
H	-0.87471600	5.23220800	-0.70035000
N	0.35052600	2.08289900	-0.40483800

H	-0.87496600	-5.23214000	-0.70047800
N	0.35042700	-2.08289600	-0.40489200
C	0.33823900	-0.00000600	-2.38215800
O	0.53389000	0.00014300	-3.53127800

4. Other Intermediates

Ferrocene (Cp_2Fe)

	x	y	z
C	0.00000000	1.64888800	-1.22637400
C	-1.16597000	1.64997300	-0.37879000
C	-0.72037400	1.64876700	0.99226000
C	0.72130300	1.64850500	0.99200900
C	1.16649400	1.64864900	-0.37922000
H	-0.00041300	1.62024200	-2.31773500
H	-2.20414100	1.62271900	-0.71571500
H	-1.36174700	1.61929400	1.87524700
H	1.36293300	1.61931100	1.87473000
H	2.20441600	1.62004600	-0.71654600
Fe	0.00000000	0.00000000	0.00005500
C	0.72037400	-1.64876700	0.99226000
C	-0.72130300	-1.64850500	0.99200900
C	1.16597000	-1.64997300	-0.37879000
H	1.36174700	-1.61929400	1.87524700
C	-1.16649400	-1.64864900	-0.37922000
H	-1.36293300	-1.61931100	1.87473000
C	0.00000000	-1.64888800	-1.22637400
H	2.20414100	-1.62271900	-0.71571500
H	-2.20441600	-1.62004600	-0.71654600
H	0.00041300	-1.62024200	-2.31773500

Ferrocenium (Cp_2Fe^+)

	x	y	z
C	1.74968900	-1.16349600	-0.37505200
C	1.79843700	-0.00754200	-1.22003100
C	1.75101700	1.15878000	-0.38952100
C	1.67844000	0.72901500	0.98359400
C	1.67766700	-0.71647700	0.99273300
H	1.73788200	-2.20164300	-0.71294000
H	1.80969400	-0.01426800	-2.31209700
H	1.73936300	2.19267700	-0.74022700
H	1.64251300	1.37967700	1.85926400
H	1.64165800	-1.35600900	1.87656800
Fe	0.00000000	-0.00016200	0.00608400
C	-1.67844100	0.72901100	0.98359600
C	-1.75101800	1.15878100	-0.38951700
C	-1.67766700	-0.71648200	0.99273000
H	-1.64251300	1.37967000	1.85926900
C	-1.79843700	-0.00753800	-1.22003100
H	-1.73936500	2.19267900	-0.74021900
C	-1.74968800	-1.16349500	-0.37505600
H	-1.64165700	-1.35601700	1.87656300
H	-1.80969400	-0.01426000	-2.31209700
H	-1.73787900	-2.20164100	-0.71294800

Phenol

	x	y	z
C	1.14079400	-1.22718500	-0.00006900
C	-0.26450300	-1.20893800	0.00014900
C	-0.94593500	0.02337000	0.00046400
C	-0.22405300	1.23206200	0.00001700
C	1.17811200	1.19936200	-0.00001800
C	1.87010800	-0.02635100	-0.00003800
H	1.66405900	-2.19042600	-0.00019500
H	-0.82954000	-2.15088200	0.00012300
H	-0.77337000	2.17932100	-0.00028400
H	1.73530300	2.14341100	-0.00018100
H	2.96523100	-0.04434800	-0.00037800
O	-2.32341400	0.11259400	-0.00004500
H	-2.70151300	-0.79174900	-0.00174700

Phenoxide Anion

	x	y	z
C	-1.11067700	-1.21050300	-0.00000800
C	0.28969600	-1.22057900	-0.00006800
C	1.08584100	-0.00001500	0.00008500
C	0.28968500	1.22057900	-0.00006100
C	-1.11067400	1.21051300	-0.00000600
C	-1.84356600	-0.00000400	0.00005100
H	-1.65425400	-2.16986200	-0.00002100
H	0.84030100	-2.17273800	-0.00006100
H	0.84033800	2.17270900	-0.00008600
H	-1.65427200	2.16986000	-0.00002400
H	-2.94178000	0.00000600	0.00006500
O	2.37098000	0.00001000	0.00002100

Formate

	x	y	z
C	0.00000000	0.31989600	0.00000000
H	0.00086900	1.47020200	0.00000000
O	1.15288400	-0.21227100	0.00000000
O	-1.15299300	-0.21142700	0.00000000

CO_2

	x	y	z
C	0.00000000	0.00000000	0.00000000
O	0.00000000	0.00000000	1.18139400
O	0.00000000	0.00000000	-1.18139400

CO

	x	y	z
C	0.00000000	0.00000000	-0.65663200
O	0.00000000	0.00000000	0.49247400

Bicarbonate

	x	y	z
C	-0.16308000	0.06914700	0.00010300
O	0.09264800	1.31202000	-0.00003000
O	1.04383800	-0.77308300	-0.00002300
O	-1.23326000	-0.57916500	-0.00002800
H	1.75266500	-0.09305900	0.00002500

Acetonitrile

	x	y	z
C	0.00000000	0.00000000	0.27757800
N	0.00000000	0.00000000	1.45163700
C	0.00000000	0.00000000	-1.18619500
H	0.00000000	1.03488800	-1.56992100
H	0.89624000	-0.51744400	-1.56992100
H	-0.89624000	-0.51744400	-1.56992100

[Fe(bpy2PYMe)S]$^{2+}$

	x	y	z
C	-2.36442900	-1.30338200	-2.66749100
C	-1.83319300	-0.65637500	-1.53892600
C	0.10960600	-2.01672200	-1.64974000
C	-0.37494600	-2.68927200	-2.78309900
C	-1.60262800	-2.28576100	-3.32574900
H	-3.36768900	-1.06591100	-3.02762100
H	0.17503600	-3.52965400	-3.21566100
H	-1.99852000	-2.77786900	-4.21967000
C	-2.64543000	0.27551700	-0.59077700
C	-2.57130600	-0.38622200	0.81051600
C	-3.71509600	-0.76034700	1.53689600
C	-1.19643700	-1.23260000	2.51739100
C	-3.58150500	-1.38505000	2.78577700
H	-4.71214500	-0.57248400	1.13352200
C	-2.29437100	-1.63275100	3.28271400
H	-0.17346600	-1.40774700	2.85826200
H	-4.47052100	-1.67899400	3.35306200
H	-2.13486800	-2.13219000	4.24296300
C	-1.94491800	1.64675300	-0.53730600
C	-2.55674000	2.87700300	-0.83126500
C	0.18553100	2.66503900	-0.53916300
C	-1.76548000	4.03595700	-0.91090800
H	-3.63163100	2.93868200	-1.01374000
C	-0.37209600	3.92393000	-0.81606100
H	-2.23000200	5.00636600	-1.11235900
H	0.26733000	4.79280900	-0.99514600
N	-0.61769700	1.58817600	-0.27580100
N	-0.56526500	-0.94080400	-1.14051400
C	1.60075600	2.29190800	-0.68083100
C	2.63429000	3.18879900	-1.00228500
C	3.05783500	0.46951500	-0.87824700
C	3.91548700	2.69364300	-1.27899700
H	2.43607400	4.26362600	-1.04841000
C	4.12419300	1.30668500	-1.23353500
H	3.19882100	-0.61163800	-0.81746100
H	4.73076800	3.37693800	-1.53716600
H	5.09994700	0.86870400	-1.46397500
C	1.22606900	-2.43120600	-0.78474300
C	2.07255600	-3.52668700	-1.03000200
C	2.23861700	-2.06404000	1.30387800
C	3.01724400	-3.89938400	-0.06337300
H	1.98778700	-4.08879200	-1.96475600
C	3.09162300	-3.16165700	1.13025200
H	2.28929000	-1.44240400	2.20242200
H	3.67912300	-4.75376000	-0.23686600
H	3.80521900	-3.42292700	1.91750100
N	1.81848300	0.93519700	-0.59548800
N	-1.32300900	-0.61321300	1.31523600
N	1.32307500	-1.69006000	0.37562300
Fe	0.26490500	-0.02169200	0.29540400
C	-4.09839900	0.41903600	-1.07842500
H	-4.59756800	-0.56129800	-1.14278900
H	-4.68804700	1.05127900	-0.39487000
H	-4.13337900	0.88468500	-2.07721500
N	0.96140600	0.78758400	1.90353400
C	1.32568100	1.33913500	2.87308900
C	1.77307400	2.02312900	4.08108200
H	1.20782000	2.96195400	4.21909900
H	1.61228300	1.38446300	4.96763600
H	2.84833800	2.26444700	4.01058100

[Fe(bpy2PYMe)S]$^{+}$

	x	y	z
C	-2.28496900	-1.25131900	-2.74195600
C	-1.79403300	-0.65085600	-1.57618700
C	0.19872500	-1.95660800	-1.69168300
C	-0.23542000	-2.55703200	-2.89696000
C	-1.45769100	-2.16769500	-3.44252100
H	-3.29430600	-1.04320500	-3.10188900
H	0.36412700	-3.33787600	-3.37488300
H	-1.81093100	-2.61501000	-4.37718100
C	-2.64356800	0.21707800	-0.60429600
C	-2.55886600	-0.47939500	0.77817800
C	-3.69696000	-0.91125700	1.48296800
C	-1.17903400	-1.32851900	2.48197300
C	-3.56022100	-1.56538800	2.71578200
H	-4.69385200	-0.74451700	1.06992000
C	-2.27150800	-1.78460900	3.22261400
H	-0.15308900	-1.48135600	2.82531700
H	-4.44658100	-1.90363300	3.26250100
H	-2.10614000	-2.30570900	4.17050900
C	-1.98797200	1.60845800	-0.50278900
C	-2.63221000	2.82213200	-0.77160700
C	0.12321900	2.68799100	-0.43835400
C	-1.87070300	4.01820400	-0.77274300
H	-3.70094500	2.85483700	-0.99203100
C	-0.48307500	3.94569500	-0.65315400
H	-2.36344100	4.98174300	-0.93698000
H	0.13236500	4.84315100	-0.76998200
N	-0.66631500	1.57481300	-0.20530000
N	-0.52623200	-0.90444100	-1.16220300
C	1.52204500	2.35192600	-0.62939800
C	2.53783500	3.26850000	-0.98654200
C	3.01891900	0.55430600	-0.89057200
C	3.81306100	2.80037800	-1.30633500
H	2.31342300	4.33880900	-1.03001500
C	4.05697100	1.41108100	-1.27567200
H	3.17797300	-0.52539800	-0.83658000
H	4.60465200	3.50166400	-1.59040900
H	5.03492300	0.99479400	-1.53408300
C	1.26638700	-2.38972300	-0.81612100
C	2.13071300	-3.48641900	-1.05365200
C	2.27858200	-2.03729200	1.29155000
C	3.06835100	-3.85880200	-0.09270900
H	2.04810900	-4.04438400	-1.99185500
C	3.14018300	-3.12130400	1.11624200
H	2.32322000	-1.42165400	2.19597200
H	3.73352200	-4.71034400	-0.26830100
H	3.85797600	-3.37836900	1.90065700
N	1.78458800	0.99245600	-0.55613300
N	-1.30919100	-0.67968600	1.29559300
N	1.35738800	-1.65637500	0.36953800
Fe	0.26428500	-0.01400100	0.33243000
C	-4.09870300	0.32594200	-1.09236300
H	-4.56430500	-0.66878400	-1.18471500
H	-4.70829400	0.92648000	-0.39683400
H	-4.14004200	0.81459600	-2.07943900
N	0.93483300	0.76163100	1.93030300
C	1.30631100	1.28832700	2.91293700
C	1.76580600	1.95683700	4.12606800

H	1.19464300	2.88801100	4.29024800
H	1.63030400	1.30457700	5.00701800
H	2.83673100	2.21299300	4.04109100

[Fe(bpy2PYMe)]$^{+}$

	x	y	z
C	-2.42221400	-1.87050600	-2.15289800
C	-1.81298500	-1.04246800	-1.19996000
C	0.24682400	-2.18809400	-1.48958500
C	-0.31466100	-3.03016900	-2.46238900
C	-1.65193600	-2.83691000	-2.83242300
H	-3.49054700	-1.78607800	-2.36539700
H	0.27438400	-3.83974600	-2.90388300
H	-2.11255700	-3.46841700	-3.59821800
C	-2.55409500	-0.04533700	-0.28051700
C	-2.20426800	-0.41326400	1.19617900
C	-3.18220100	-0.79312600	2.13434800
C	-0.53454100	-0.63598400	2.83545000
C	-2.81167300	-1.09776900	3.45231800
H	-4.23392200	-0.85445900	1.84666700
C	-1.45732900	-1.01542200	3.81211600
H	0.53327800	-0.55172700	3.06284800
H	-3.57107400	-1.39177400	4.18430600
H	-1.11750300	-1.23605500	4.82862400
C	-2.01378200	1.37412400	-0.52267000
C	-2.82319100	2.46721300	-0.85721300
C	-0.10326000	2.77772000	-0.48110500
C	-2.25408800	3.74592000	-1.00933500
H	-3.89797000	2.33647200	-0.99839000
C	-0.88131000	3.90109600	-0.80799800
H	-2.88131100	4.60361200	-1.27096500
H	-0.41442400	4.88569100	-0.90158200
N	-0.66323700	1.52149500	-0.35576600
N	-0.47208900	-1.12941400	-0.97339200
C	1.33148500	2.77839600	-0.25205900
C	2.16660100	3.90595200	-0.38419000
C	3.18100500	1.43154500	0.25944000
C	3.54169200	3.78131600	-0.17991400
H	1.73518100	4.87389800	-0.65540800
C	4.05699000	2.50877900	0.14102600
H	3.56504200	0.43485800	0.48436900
H	4.20200200	4.64841000	-0.28024000
H	5.12918400	2.34927700	0.29089100
C	1.51861200	-2.36306200	-0.79397300
C	2.42515100	-3.42062900	-0.99910900
C	2.71416900	-1.66780600	1.10913800
C	3.49205200	-3.60245300	-0.11330400
H	2.27435700	-4.11023300	-1.83526500
C	3.61420900	-2.72811700	0.98524300
H	2.78932600	-0.96114000	1.94255600
H	4.20078100	-4.42384400	-0.25797100
H	4.40241100	-2.85883100	1.73300200
N	1.82987200	1.53275400	0.09025900
N	-0.88818100	-0.34301100	1.55737900
N	1.70414100	-1.43814800	0.22262600
Fe	0.47156000	0.07385300	0.12306700
C	-4.07270200	-0.11591800	-0.52602600
H	-4.45135500	-1.13840400	-0.36371300
H	-4.62194900	0.56037400	0.14907600
H	-4.31475000	0.17204800	-1.56232400

Fe(bpy2PYMe)]^{0}

	x	y	z
C	-2.31440300	-2.20407500	-2.00474500
C	-1.74132600	-1.26262300	-1.14424400
C	0.38935400	-2.28799200	-1.38651000
C	-0.14649600	-3.25999900	-2.25079800
C	-1.49409900	-3.18356000	-2.61678100
H	-3.39161700	-2.20895000	-2.19025400
H	0.48290600	-4.08202900	-2.60813600
H	-1.92808700	-3.91493500	-3.30642300
C	-2.53241200	-0.29676300	-0.23576900
C	-2.05185900	-0.55108400	1.22778700
C	-2.91962200	-0.99587900	2.24129800
C	-0.26493800	-0.54920800	2.76869600
C	-2.44373800	-1.22450300	3.54110000
H	-3.97535300	-1.17155700	2.02098000
C	-1.08167600	-0.99787800	3.80595300
H	0.79809200	-0.34157600	2.92287700
H	-3.12355100	-1.56947700	4.32758700
H	-0.65481800	-1.15492100	4.80200800
C	-2.17109400	1.16181700	-0.55127300
C	-3.10954500	2.15513800	-0.84625600
C	-0.41656000	2.76701400	-0.50209800
C	-2.68123200	3.49869100	-0.97721600
H	-4.16751800	1.90784500	-0.95938900
C	-1.33655100	3.80256400	-0.77512800
H	-3.40413200	4.28923100	-1.20549700
H	-0.98871900	4.83941700	-0.82298300
N	-0.83915600	1.44278500	-0.43833300
N	-0.38298200	-1.20647400	-0.97484400
C	0.99457400	2.90318000	-0.28573600
C	1.71460800	4.11648900	-0.40225600
C	3.00266300	1.72918400	0.05690400
C	3.09821900	4.13517000	-0.27838600
H	1.16533700	5.04191600	-0.60790100
C	3.75151300	2.88734100	-0.07042000
H	3.50939500	0.77265100	0.19415600
H	3.66440900	5.06825000	-0.36351900
H	4.84357100	2.82084000	-0.01412600
C	1.68221800	-2.28971600	-0.74130500
C	2.67163400	-3.28279000	-0.88410000
C	2.92102000	-1.27647800	0.98681600
C	3.81253800	-3.26483800	-0.07880600
H	2.52504400	-4.08099700	-1.62012000
C	3.91319900	-2.24869100	0.90452800
H	2.96467000	-0.49698000	1.75442900
H	4.58607300	-4.03268400	-0.18095400
H	4.75136200	-2.22003900	1.60913800
N	1.62394200	1.67701000	0.00845800
N	-0.72095900	-0.32653600	1.50334600
N	1.83424800	-1.21596700	0.14854200
Fe	0.45515200	0.12031400	0.03903100
C	-4.04560800	-0.54298600	-0.37561300
H	-4.30043000	-1.59117600	-0.14737200
H	-4.62166100	0.10790900	0.30275100
H	-4.37685500	-0.33178700	-1.40610600
